# Decitabine priming increases anti–PD-1 antitumor efficacy by promoting CD8^+^ progenitor exhausted T cell expansion in tumor models

**DOI:** 10.1172/JCI165673

**Published:** 2023-04-03

**Authors:** Xiang Li, Yaru Li, Liang Dong, Yixin Chang, Xingying Zhang, Chunmeng Wang, Meixia Chen, Xiaochen Bo, Hebing Chen, Weidong Han, Jing Nie

**Affiliations:** 1 Department of Bio-therapeutic, the First Medical Centre, Chinese PLA General Hospital, Beijing, China.; 2 Institute of Health Service and Transfusion Medicine, Beijing, China.; 3 State Key Laboratory of Stem Cell and Reproductive Biology, Institute of Zoology, Chinese Academy of Sciences, Beijing, China.; 4 Changping Laboratory, Beijing, China.

**Keywords:** Immunology, Oncology, Cancer immunotherapy, T cells

## Abstract

CD8^+^ exhausted T cells (T_ex_) are heterogeneous. PD-1 inhibitors reinvigorate progenitor T_ex_, which subsequently differentiate into irresponsive terminal T_ex_. The ability to maintain a capacity for durable proliferation of progenitor T_ex_ is important, but the mechanism remains unclear. Here, we showed CD8^+^ progenitor T_ex_ pretreated with decitabine, a low-dose DNA demethylating agent, had enhanced proliferation and effector function against tumors after anti–PD-1 treatment in vitro. Treatment with decitabine plus anti–PD-1 promoted the activation and expansion of tumor-infiltrated CD8^+^ progenitor T_ex_ and efficiently suppressed tumor growth in multiple tumor models. Transcriptional and epigenetic profiling of tumor-infiltrated T cells demonstrated that the combination of decitabine plus anti–PD-1 markedly elevated the clonal expansion and cytolytic activity of progenitor T_ex_ compared with anti–PD-1 monotherapy and restrained CD8^+^ T cell terminal differentiation. Strikingly, decitabine plus anti–PD-1 sustained the expression and activity of the AP-1 transcription factor JunD, which was reduced following PD-1 blockade therapy. Downregulation of JunD repressed T cell proliferation, and activation of JNK/AP-1 signaling in CD8^+^ T cells enhanced the antitumor capacity of PD-1 inhibitors. Together, epigenetic agents remodel CD8^+^ progenitor T_ex_ populations and improve responsiveness to anti–PD-1 therapy.

## Graphical abstract

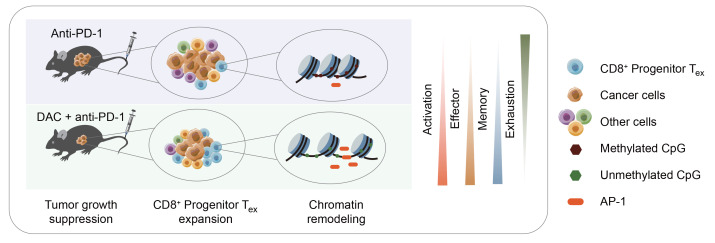

## Introduction

Persistent antigen stimulation causes CD8^+^ T cells to become functionally exhausted, with upregulation of programmed death-1 (PD-1) and other inhibitory receptors, perturbed proliferation and cytokine secretion, impaired immune memory, and altered metabolism (1). PD-1 immune checkpoint blockade (ICB) can reinvigorate CD8^+^ exhausted T cells (T_ex_), potentiate effector function, and enhance tumor control, but most patients fail to achieve long-lasting clinical response (2). Completely understanding the mechanisms underlying anti–PD-1 action and how to induce a high response to PD-1 inhibitors is crucial.

T cell exhaustion is a progressive developmental process and T_ex_ are heterogeneous (3–5). When encountering sustained tumor antigens, naive T cells undergo dynamic epigenetic remodeling, differentiate into a plastic reprogrammable chromatin state, and finally transform into a fixed dysfunctional chromatin state (6). Distinct epigenetic remodeling in T_ex_ subsets alters the TF binding profile and transcriptional network, and controls the formation and transition of T_ex_ subsets. TFs such as NR4A, TOX, and IRF4 play critical roles in the activation of inhibitory receptors and negative regulators and favor terminal differentiation, while TCF-1 promotes the generation and persistence of progenitor T_ex_ (7–11). The PD-1^+^TCF-1^+^TIM-3^–^ progenitor T_ex_ population has greater reprogrammability and retains its proliferative capacity, expands after PD-1/PD-L1 blockade, and gives rise to terminally exhausted cells (12). Terminal T_ex_ possess a high level of PD-1 and coexpress other inhibitory receptors such as TIM-3, CD38, CD101, TIGIT, which retain some cytotoxic potential, but are nonreprogrammable and unable to proliferate following treatment with PD-1/PD-L1 inhibitors (6, 12, 13). Approaches for more effective activation and maintenance of progenitor T_ex_ during anti–PD-1/PD-L1 therapy are crucial.

Blocking PD-1/PD-L1 signaling induced rejuvenation of T_ex_ but could not reverse the exhaustion-related epigenetic signature. Some open chromatin regions became locked after treatment, and the reinvigorated T_ex_ may be short-lived and lack long-term antitumor immune responses (14). DNA methylation acts as a crucial epigenetic mechanism for gene expression regulation and promotes terminal differentiation of T_ex_ (15). It has been revealed that low-dose decitabine, a DNA hypomethylating agent, could enhance the activation and cytolytic activity of CD8^+^ and CD4^+^ T cells, both in vitro and in vivo (16, 17). We previously reported that among relapsed/refractory patients with classical Hodgkin lymphoma (cHL), 71% of anti–PD-1–naive patients were evaluated as having achieved complete remission (CR) after decitabine-plus-anti–PD-1 combination, versus 32% of patients having achieved CR with anti–PD-1 single-agent camrelizumab (18). Moreover, we observed that all 3 patients with advanced metastatic non–small lung cancer who were considered unfavorable factors for PD-1 inhibitors, acquired partial responses after combination therapy with decitabine plus anti–PD-1 (DP therapy) (19). However, the underlying mechanisms for the improved antitumor capacity of DP therapy compared with anti–PD-1 monotherapy was elusive, which hampered the clinical application of the combination of decitabine plus anti–PD-1 in solid tumors. We hypothesized that low-dose decitabine priming could modulate the fate and revival of T_ex_ treated by PD-1 blockade therapy, which would favor and boost the clinical efficacy of anti–PD-1 therapy.

Here, we tested the antitumor capacity of the combination of DNA decitabine and PD-1 inhibitor in vitro in a tumor cell and T cell coculture system and in mice bearing mouse MC38 solid tumors or EG7 lymphomas, and confirmed that DP therapy elicited superior antitumor immunity compared with anti–PD-1 single-agent. By using flow cytometry analysis, single cell RNA-Seq, T cell receptor–Seq, mass cytometry, and assay of transposase-accessible chromatin using sequencing, we identified the substantially expanding T subset after treatment with DP therapy and investigated the differentially expressed genes, chromatin alterations, and T cell clonality remodeling. We compared these with anti–PD-1 monotherapy to clarify the molecular programs underlying epi-immunotherapy–mediated potent T cell reactivation and investigated the differentially expressed genes, chromatin alterations and T-cell clonality remodeling compared with anti-PD-1 monotherapy, to clarify the molecular programs underlying epi-immunotherapy–mediated potent T cell reactivation.

## Results

### Low-dose decitabine-pretreated CD8^+^ T cells have increased cytotoxicity against tumors following anti–PD-1 treatment both in vitro and in vivo.

**Figure 1:**
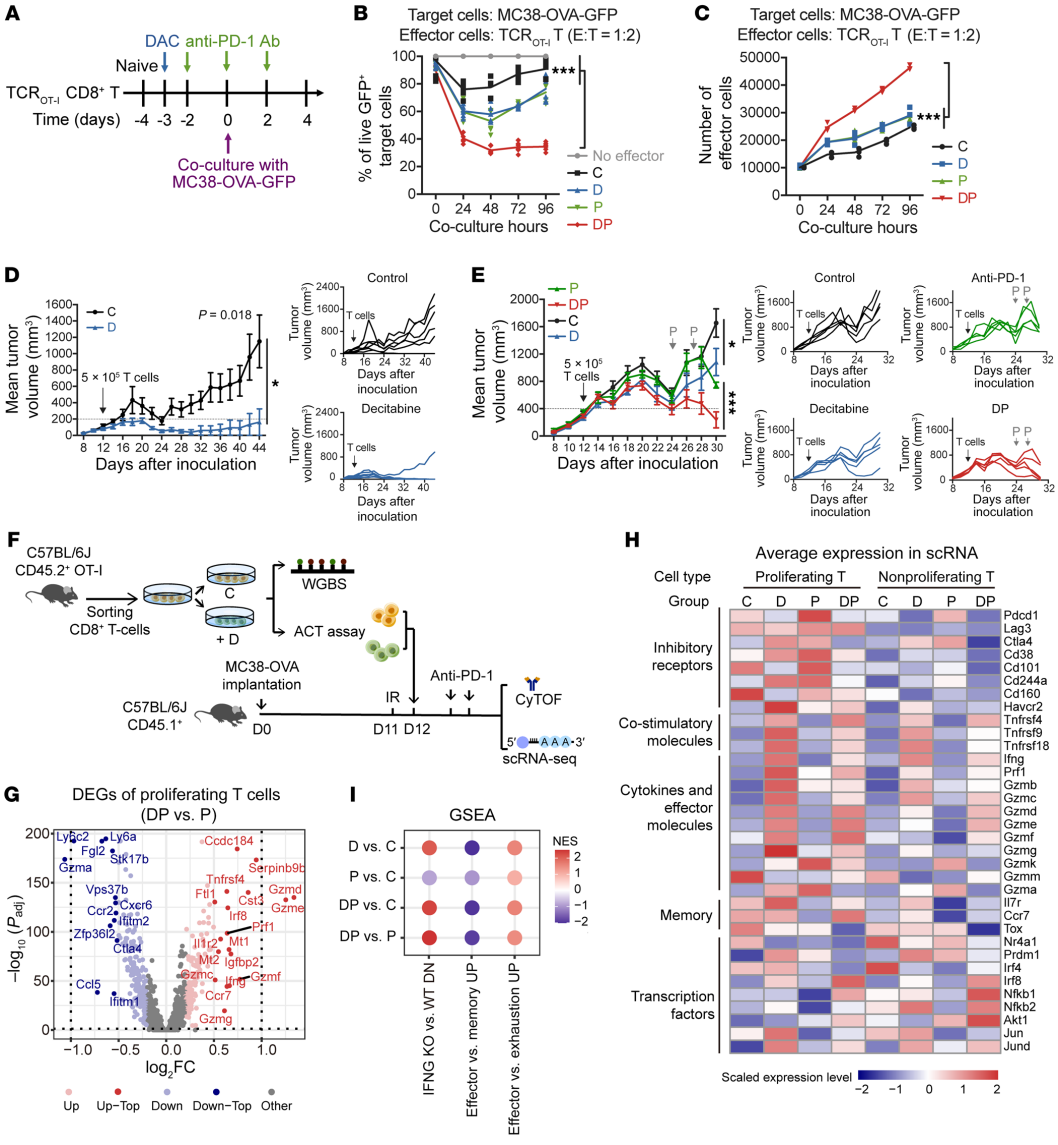
Low-dose decitabine-pretreated CD8^+^ T cells have increased cytotoxicity against tumors following anti–PD-1 treatment. (**A**) Experimental timeline. Purified naive CD8^+^ T cells from TCR_OT-I_ mice were activated, treated with PBS (C), 10 nM decitabine (D), anti–PD-1 antibody (P) or the combination (DP), and cocultured with MC38-OVA-GFP cells at E: T ratio of 1:2 (**A**–**C**). (**B**) Frequency of live GFP^+^ MC38-OVA cells. Results are pooled from 2 experiments with *n* = 6 per group. 2-way ANOVA analysis. (**C**) Absolute number of CD8^+^ T cells (*n* = 3). 2-way ANOVA analysis. (**D**) PBS or decitabine-treated CD45.2^+^CD8^+^ TCR_OT-1_ cells were transferred into MC38-OVA-bearing CD45.1^+^ C57BL/6J mice on day 12 when tumor size was below 200 mm^3^ (*n* = 6). Shown are average and individual tumor growth curves. Data are represented as mean ± SEM. 2-way ANOVA analysis. (**E**) PBS or decitabine-treated CD45.2^+^CD8^+^ TCR_OT-1_ cells were transferred into MC38-OVA-bearing CD45.1^+^ C57BL/6J mice on day 12 when tumor size was between 200 and 400 mm^3^, followed by anti–PD-1 treatment as indicated (*n* = 5 per group). Shown are average and individual tumor growth curves. Data are represented as mean ± SEM. 2-way ANOVA analysis. (**F**) Experimental design. (**G**) Volcano plot showing DEGs of proliferating T cells between the DP group and the P group. Genes with *P*_adj_ < 0.05 (2-sided unpaired Wilcoxon test, Bonferroni correction) and absolute log_2_ fold change (FC) ≥ 0.2 are identified as DEGs. Genes with *P*_adj_ < 0.05 and absolute log_2_ FC ≥ 0.5 are labeled. (**H**) Heatmap showing scaled expression values of the indicated genes. Colors represent averaged z-scores of expression level. (**I**) GSEA of proliferating T cells generated by the immunologic signature gene sets of MSigDB. The 3 terms were from GSE369 and GSE41867. Colors of circles represent the normalized enrichment score (NES) calculated by GSEA for each signature. *P < 0.05; ***P < 0.001.

To investigate whether low-dose decitabine pretreatment enhanced anti–PD-1-induced activation of CD8^+^ T cells, an in vitro tumor cell and T cell coculture model was used. CD8^+^ naive T cells from OT-I transgenic mice were purified, anti-CD3/CD28 activated, and treated with PBS (C group), 10 nM low-dose decitabine (D group), anti–PD-1 (P group) or together (DP group) in vitro. These OVA-specific CD8^+^ T cells were cocultured with MC38-OVA-GFP colon cancer cells for 4 days, at an effect and target ratio of 1-to-2, and anti–PD-1 antibody was added as indicated (Figure 1A). Strikingly, decitabine-pretreated CD8^+^ T cells acquired increased cytolysis activity against tumor cells with anti–PD-1 treatment at different time points and with distinct effector and target ratios (1:1, 1:2, 1:4) (Figure 1B and Supplemental Figure 1, A and B). Decitabine and anti–PD-1 treatment synergistically promoted CD8^+^ T cell expansion with high Ki67 levels and acquired increased capacity to coproduce IFN-γ and TNF-α (Figure 1C and Supplemental Figure 1, C and D). Moreover, similar results were observed upon OVA_257–264_ peptide–stimulated TCR_OT-I_ T cells (Supplemental Figure 1, E–G).

As expected, decitabine treatment of activated CD8^+^ T cells resulted in a decrease of DNA methylation at the promoter region, which was enriched in genes associated with inositol phosphate metabolism, MAPK signaling pathway, and T cell differentiation, as detected by whole genome bisulfite sequencing (WGBS; Supplemental Figure 1, H–K and Supplemental Table 2). To evaluate whether decitabine-pretreated CD8^+^ T cells contributed to improved antitumor response in vivo, we detected the capacity of decitabine-primed CD45.2^+^CD8^+^ T cell therapy in congenetic CD45.1^+^ C57BL/6J mice bearing MC38-OVA tumors. When tumors were small (volume under 200 mm^3^), adoptive cell therapy (ACT) of low numbers (5 × 10^5^) of decitabine-pretreated CD45.2^+^OVA-specific CD8^+^ TCR_OT-I_ cells could eliminate tumors, while control T cells initially suppressed tumor growth but tumors began growing after 12 days (Figure 1D). For larger tumors (200–400 mm^3^), 5 × 10^5^ CD8^+^ TCR_OT-I_ cells were transferred, followed by anti–PD-1 infusion 12 days later, when tumors began to regrow, we observed that adoptive transfer of decitabine-primed T cells plus anti–PD-1 significantly restrained tumor growth compared with control T cells plus anti–PD-1 (Figure 1, E and F).

In this model, a higher number of transferred CD45.2^+^TCR_OT-I_ cells was detected in tumors in the DP group than in the P group, as analyzed by CyTOF (Supplemental Figure 2A). Furthermore, the expression levels of inhibitory receptors TIM-3, LAG-3, and CD38 in transferred CD45.2^+^TCR_OT-1_ cells were downregulated in the DP group compared with the P group, while T-bet expression was upregulated in DP-treated cells (Supplemental Figure 2, B and C). We next sorted the transferred CD45.2^+^TCR_OT-I_ cells in tumors from the 4 groups and conducted the 5′ single cell RNA-Sequencing (scRNA-Seq) and paired T cell receptor sequencing (scTCR-Seq). A total of 37,447 CD8^+^ T cells were collected, and similar expression patterns were detected, since most cells shared the same TCR clonotype (Supplemental Figure 3, A and B). We divided these cells into proliferating and nonproliferating subgroups based on the results of unsupervised clustering and cell cycle stage (Supplemental Figure 3, C–E). A slightly elevated frequency of proliferating cells was observed in the DP group compared with the other groups (Supplemental Figure 3F). Gene Ontology (GO) analysis revealed that the upregulated genes of proliferating cells in the P group were enriched in T cell activation, cell killing, and immune effector processes compared with the C group (Supplemental Figure 3, G and H). The upregulated genes in proliferating cells in the DP group compared with the P group were enriched in ribonucleoprotein complex biogenesis, protein folding, T cell activation, proliferation, and immune effector process, among which the cytotoxicity-related genes were the most dramatically increased in the DP group, such as *Gzmd/e/f/g* (*Gzmh* in humans), *Prf1*, and *Ifng* (IFNγ) (Figure 1G, Supplemental Figure 3I, and Supplemental Table 3). We noticed that, compared with untreated cells, both D group and DP group T cells showed increased costimulatory molecules *Tnfrsf4* (OX40), *Tnfrsf9* (4-1BB), *Tnfrsf18*, and a series of cytokines and effector molecules such as *Ifng*, *Prf1*, *Gzmb/c/d/e/f/g/k*, and TFs *Nfkb1/Nfkb2/Irf8/JunD* (Figure 1H). Gene-set enrichment analysis (GSEA) suggested that CD8^+^ T cells from decitabine therapy and combination therapy of decitabine plus anti–PD-1 displayed enrichment for effector and memory signatures (Figure 1I). In addition, lower expression of immune inhibitory receptors (*Pdcd1*, *Cd38*, *Ctla4*, and *Cd244*) was detected in CD8^+^ T cells after combination therapy compared with anti–PD-1 monotherapy. Similar effects were detected in the nonproliferating subgroup (Supplemental Figure 3, J and K). These results indicated that decitabine treatment can directly regulate the antitumor activity of CD8^+^ T cells, and decitabine-pretreated CD8^+^ T cells had high cytotoxicity after PD-1 blockade both in vitro — against tumor cells — and in mouse tumor models.

### Progenitor T_ex_ rather than terminal T_ex_ gain improved activation and function following treatment with decitabine plus anti–PD-1.

**Figure 2:**
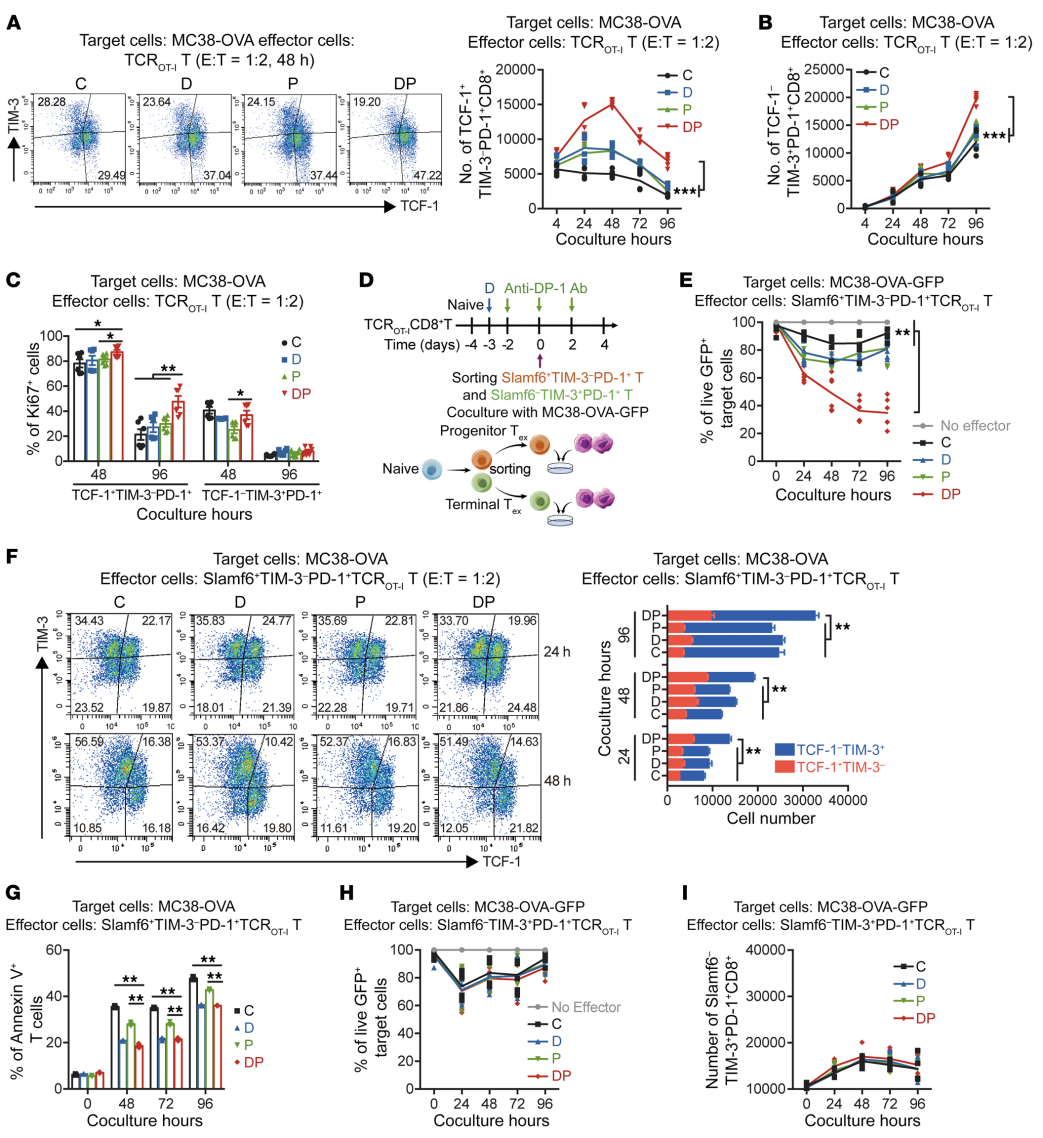
In vitro DP treatment significantly increases the effector function of CD8^+^ progenitor T_ex_ rather than terminal T_ex_. (**A** and **B**) Absolute numbers of TCF-1^+^TIM-3^–^PD-1^+^ progenitor T_ex_ (**A**) and TCF-1^–^TIM-3^+^PD-1^+^ terminal T_ex_ (**B**) at the indicated times of coculture as in Figure 1A. Results are pooled from 2 experiments with *n* = 6 per group. The representative FACS plots for TCF-1^+^TIM-3^–^PD-1^+^ cells and their frequencies are shown. 2-way ANOVA analysis. (**C**) Frequency of Ki67^+^ cells in TCF-1^+^TIM-3^–^PD-1^+^ progenitor T_ex_ and TCF-1^–^TIM-3^+^PD-1^+^ terminal T_ex_ at the indicated times of coculture (*n* = 6), by 1-way ANOVA analysis. (**D**) Experimental design. Slamf6^+^TIM-3^–^PD-1^+^ (surrogate for TCF-1^+^TIM-3^–^) progenitor T_ex_ and Slamf6^–^TIM-3^+^PD-1^+^ terminal T_ex_ were isolated and cocultured with MC38-OVA-GFP (or MC38-OVA) cells at E-to-T ratio of 1:2. (**E**) Frequency of live GFP^+^ target cells during the coincubation of MC38-OVA-GFP and progenitor T_ex_ at the indicated times (*n* = 6). 2-way ANOVA analysis. (**F**) Absolute numbers of progenitor and terminal T_ex_ during the coincubation of MC38-OVA and progenitor T_ex_ at the indicated times (*n* = 6). The representative FACS plots for TCF-1^+^TIM-3^–^PD-1^+^ progenitor T_ex_ and TCF-1^+^TIM-3^–^PD-1^+^ terminal T_ex_ cells and their frequencies are shown. Data are represented as mean ± SEM, by 1-way ANOVA analysis. (**G**) Frequency of Annexin V^+^ apoptotic T cells during the coincubation of MC38-OVA and progenitor T_ex_ at the indicated times (*n* = 3), by 1-way ANOVA analysis. (**H**) Frequency of live GFP^+^ target cells during the coincubation of MC38-OVA-GFP and terminal T_ex_ at the indicated times by flow cytometry analysis. (**I**) Absolute number of terminal T_ex_ during coincubation at the indicated times. *P < 0.05; **P < 0.01; ***P < 0.001.

In the in vitro coculture system, the total number of CD8^+^ T cells was significantly increased in the DP group compared with other groups, with a high Ki67 level (Supplemental Figure 4, A and B). The TCF-1^+^TIM-3^–^PD-1^+^CD8^+^ progenitor T_ex_ were reported to expand after PD-1 blockade therapy (12). Consistently, as shown in Figure 2A, TCF-1^+^TIM-3^–^PD-1^+^CD8^+^ progenitor T_ex_ expanded after anti–PD-1 treatment early on, when encountering tumor cells, and then progressively differentiated into TCF-1^–^TIM-3^+^PD-1^+^CD8^+^ terminal T_ex_. Notably, both proportion and absolute number of progenitor T_ex_ were markedly increased in the DP group compared with those in the single-agent group, and the amount of progenitor T_ex_ was maintained at a high level for more than 2 days (Figure 2A and Supplemental Figure 4C). Later on, we also detected incremental numbers of terminal T_ex_ after DP treatment. The frequencies among distinct groups were comparable, which could be due to the differentiation of progenitor T_ex_ since all these terminal T_ex_ had nearly low proliferative potential (Figure 2B and Supplemental Figure 4D). Contrastingly, progenitor T_ex_ in the DP group showed higher Ki67 level even after 4 days of tumor antigen stimulation (Figure 2C). However, the enhanced effect of the DP combination treatment compared with anti–PD-1 treatment of CD8^+^ T cells was no longer observed if decitabine was administered 5 days after TCR stimulation, when T cells were in a differentiated state (Supplemental Figure 4E). These data suggested that decitabine priming resulted in durable expansion of progenitor T_ex_ following PD-1 blockade.

To further validate the specific T_ex_ subset that responded to decitabine plus anti–PD-1, we used the combination of Slamf6 and TIM-3 to differentiate the progenitor T_ex_ and terminal T_ex_ (12). After in vitro decitabine, anti–PD-1, or the combined pretreatment, Slamf6^+^TIM-3^–^PD-1^+^CD8^+^ progenitor T_ex_ and Slamf6^–^TIM-3^+^PD-1^+^CD8^+^ terminal T_ex_ were sorted and cocultured with MC38-OVA-GFP cells (Figure 2D). We observed that decitabine-pretreated progenitor T_ex_ responded better to anti–PD-1 and were present in higher numbers after anti–PD-1 treatment (Figure 2, E and F and Supplemental Figure 4F). Besides the elevated proliferation potential, DP treatment showed decreased apoptosis ratio in progenitor T_ex_ compared with anti–PD-1 alone (Figure 2G). However, DP therapy had a minimal effect on terminal T_ex_ (Figure 2, H and I and Supplemental Figure 4G). These results demonstrated that decitabine priming directly enhanced the proliferative capacity of less-differentiated progenitor T_ex_ in response to PD-1 blockade, while it could not reprogram terminal T_ex_ into progenitor T_ex_ cells.

### Administration of decitabine plus anti–PD-1 treatment dramatically inhibits tumor growth in vivo and reshapes the tumor microenvironment.

**Figure 3:**
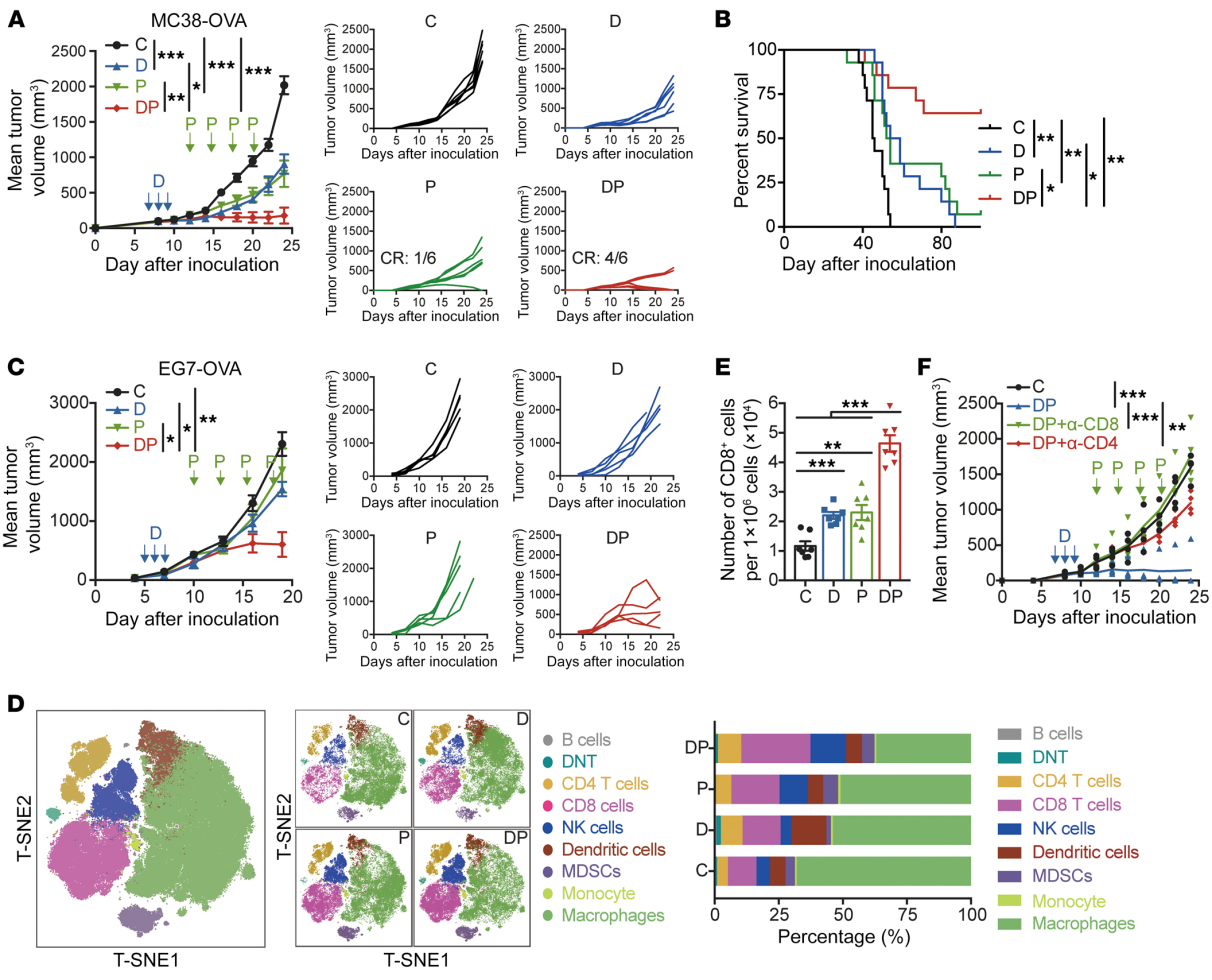
Administration of DP combination treatment inhibits tumor growth in vivo and reshapes tumor microenvironment. (**A** and **B**) C57BL/6J mice were transplanted with 1.5 × 10^5^ MC38-OVA cells, treated with PBS (C group; black), or decitabine alone (0.2 mg/kg per mouse, days 7–9; D group, blue), or anti–PD-1 antibody alone (200 μg per mouse on days 12, 15, 18, and 21; P group, green) or decitabine plus anti–PD-1 (DP group, red) as indicated. Tumor sizes were examined every other day. (**A**) Shown are average and individual tumor growth curves (*n* = 6 per group). Data are represented as mean ± SEM, by 2-way ANOVA analysis. The number of mice in P and DP groups that acquired CR was shown. (**B**) Survival curves of each treatment group, by log-rank test. (**C**) C57BL/6J mice were implanted with 1 × 10^6^ EG7-OVA cells, treatment scheme as in **A**. Tumor sizes were measured every 3 days. The average and individual tumor curves (*n* = 5 per group) are shown. Data are represented as mean ± SEM, by 2-way ANOVA analysis. (**D**) MC38 tumor samples as in **A** were collected on day 18, followed by CyTOF assay. T-SNE plot shows all CD45^+^ cells, colored by distinct immunocytes. (**E**) Absolute number of CD8^+^ TILs per 1 × 10^6^ total cells in each group of MC38-OVA xenografts model on day 18 as in **A**. 1-way ANOVA analysis. (**F**) MC38-OVA-bearing mice were treated with decitabine (days 7–9) plus anti–PD-1 (days 12, 15, 18, and 21). Anti-CD8 or anti-CD4 antibody (200 μg per mouse) was administered twice a week starting on day 6. Shown are average tumor curves (*n* = 4 per group). 2-way ANOVA analysis. *P < 0.05; **P < 0.01; ***P < 0.001.

We investigated the in vivo antitumor effects of decitabine, anti–PD-1, or their combination in C57BL/6J mice bearing mouse MC38-OVA colon cancer cells. We noticed that DP therapy significantly suppressed tumor development and prolonged survival, while either decitabine or anti–PD-1 monotherapy only delayed tumor growth (Figure 3, A and B). Mice that achieved CR after DP treatment received reinoculation with 2 times the original number of MC38-OVA cells and all mice remained tumor-free, indicative of efficient antitumor memory response (Supplemental Figure 5A). Similar significant antitumor response was observed in mice bearing EG7-OVA lymphomas after DP treatment (Figure 3C). In cold tumor CT26 colon cancer-bearing model, DP therapy also partly impeded tumor growth (Supplemental Figure 5B).

We next profiled and compared the tumor infiltrating CD45^+^ immune cell subsets in 4 groups by CyTOF assay (Supplemental Figure 5, C–E). We detected that treatment with DP therapy resulted in a remarkable increase in the ratios of CD8^+^ T cells, CD4^+^ T cells, and NK cells, while the proportion of macrophages was decreased (Figure 3D). Consistent with our initial results, the number of tumor-infiltrated CD8^+^ T cells was significantly increased in the DP group compared with the anti–PD-1 monotherapy group (Figure 3E). It may not be due to the activation of dendritic cells via decitabine, since cytokine secretion was not increased in response to OVA presentation to CD4^+^ and CD8^+^ T cells by decitabine-pretreated bone marrow-derived dendritic cells (BMDCs) (Supplemental Figure 5, F and G). To estimate whether the response to DP was dependent on CD8^+^ T cells, we depleted either CD8^+^ or CD4^+^ T cells before and during DP treatment. CD8^+^ T cell depletion completely abrogated the antitumor activity of DP therapy, indicating the requirement of CD8^+^ T cells (Figure 3F). We also noted a potential function of CD4^+^ T cells during DP treatment; another study was under investigation. Here, we mainly analyzed the effect of DP combination on CD8^+^ T cells.

### Combination therapy with decitabine plus anti–PD-1 prominently reactivates tumor-infiltrated CD8^+^ progenitor T_ex_.

**Figure 4:**
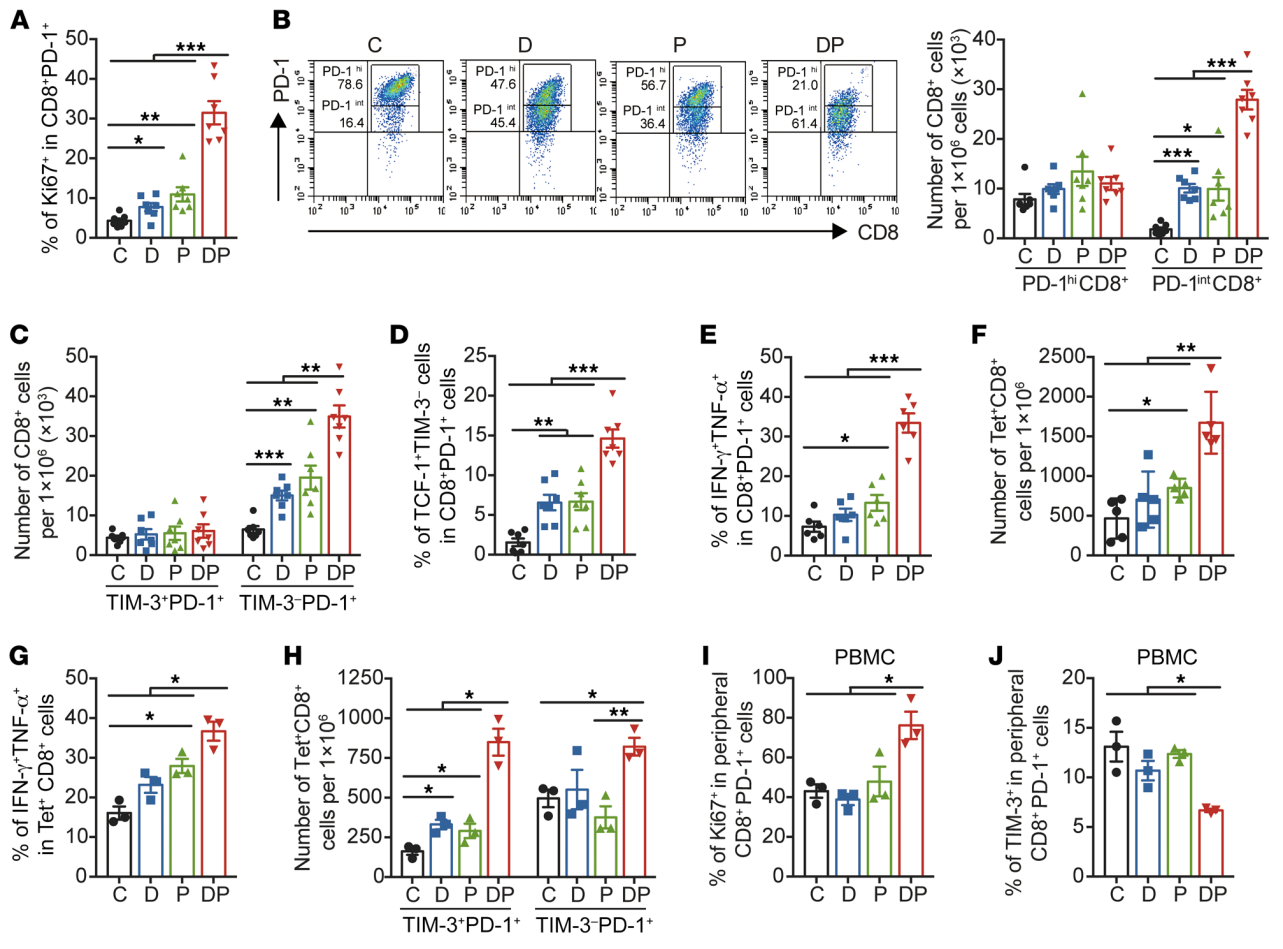
DP combination therapy prominently reactivates tumor-infiltrated CD8^+^ progenitor T_ex_. On day 18 after 2 doses of anti–PD-1, as in Figure 3A, phenotype and function of TILs from MC38-OVA tumors were detected by flow cytometry analysis (**A**–**H**). (**A**) Frequency of Ki67^+^ cells in the endogenous CD8^+^PD-1^+^ T cells, gated on CD8^+^PD-1^+^ cells. Results are pooled from 2 experiments with *n* = 7 per group. (**B**) Absolute numbers of CD8^+^ cells with PD-1 high (PD-1^hi^) and intermediate (PD-1^int^) expression in CD8^+^ TILs, gated on CD8^+^ cells (*n* = 7). The representative FACS plots for PD-1^hi^ and PD-1^int^ CD8^+^ T cells and their frequencies are shown. (**C**) Absolute numbers of PD-1^+^TIM-3^+^ and PD-1^+^TIM-3^–^ CD8^+^ TILs per 10^6^ cells (*n* = 7). (**D**) Frequency of progenitor T_ex_ (TCF^+^TIM-3^–^) in CD8^+^PD-1^+^ TILs (*n* = 7). (**E**) Frequency of IFN-γ^+^TNF-α^+^ cells in PD-1^+^CD8^+^ TILs after treated with 5-hour cell stimulation cocktail plus protein transport inhibitors (*n* = 6). (**F**) Absolute number of tetramer^+^CD8^+^ T cells per 10^6^ total cells (*n* = 5). (**G**) Frequency of IFN-γ^+^TNF-α^+^ cells in tetramer^+^CD8^+^ TILs (*n* = 3). (**H**) Absolute numbers of PD-1^+^TIM-3^+^ and PD-1^+^TIM-3^–^ tetramer^+^CD8^+^ TILs per 10^6^ total cells (*n* = 3). (**I** and **J**) Frequency of Ki67^+^ (**I**) and TIM-3^+^ (**J**) cells in CD8^+^PD-1^+^ T cells from PBMCs of MC38-OVA-bearing mice (*n* = 3). Bar plots represent the mean ± SEM. *P < 0.05; **P < 0.01; ***P < 0.001, by 1-way ANOVA analysis.

We next intended to determine the CD8^+^ subset that expanded in response to DP therapy in vivo. Flow cytometry analysis showed that the Ki67 level in the polyclonal tumor-infiltrated T_ex_ was markedly elevated after DP treatment compared with either agent alone (Figure 4A and Supplemental Figure 6A). Interestingly, both frequency and absolute number of CD8^+^ T cells with an intermediate PD-1 level were prominently increased with DP treatment (Figure 4B and Supplemental Figure 6B). Consistently, CD8^+^ T_ex_ with negative expression of other inhibitory receptors, such as TIM-3, LAG-3, or their coexpression, expanded in the DP group (Figure 4C and Supplemental Figure 6, C and D). Moreover, a higher proportion of TCF-1^+^TIM-3^–^PD-1^+^ progenitor T_ex_ was observed after DP therapy (Figure 4D and Supplemental Figure 6E). Importantly, DP-treated CD8^+^ T_ex_ could secrete more IFN-γ and TNF-α, compared with either single-agent (Figure 4E). Thus, DP treatment enhanced the proliferation and activity of CD8^+^ progenitor T_ex_ subset.

The response to DP was tumor antigen–specific, as the number of OVA-specific CD8^+^ tumor-infiltrated lymphocytes (TILs) was augmented identified by H2-K^b^ OVA_257-264_ tetramer staining (Figure 4F and Supplemental Figure 6F). The coproduction of IFN-γ and TNF-α in these tetramer^+^ (Tet^+^) CD8^+^ T cells was improved after both anti–PD-1 and DP treatment (Figure 4G). Strikingly, the count of PD-1^+^TIM-3^–^ T_ex_ was notably raised after DP combination, while the count was slightly decreased after anti–PD-1 monotherapy (Figure 4H). Moreover, the proliferation capacity of circulating CD8^+^PD-1^+^ T cells was reinforced in the DP group, with lower frequency of TIM-3^+^ cells as well (Figure 4, I and J). Additionally, significant expansion in both OVA-specific and total antigen–experienced CD8^+^ TILs was observed in mice bearing EG7-OVA lymphomas after DP treatment, with significant increase of both TIM-3^–^PD-1^+^ and TIM-3^+^PD-1^+^ cells (Supplemental Figure 7, A–D). DP treatment also potentiated CD8^+^ T cell response in EG7-OVA mice, and the frequency of IFN-γ^+^TNF-α^+^CD8^+^ T cells was approximately 7-fold higher in the DP combination group compared with anti–PD-1 group (Supplemental Figure 7, E and F). In CT26-bearing mice, DP treatment had a similar effect (Supplemental Figure 7, G–J). Collectively, these results suggested that decitabine had a synergistic effect with PD-1 inhibitor to reactivate functional progenitor CD8^+^ T_ex_.

### scRNA-Seq of TILs after treatment with decitabine plus anti–PD-1.

**Figure 5:**
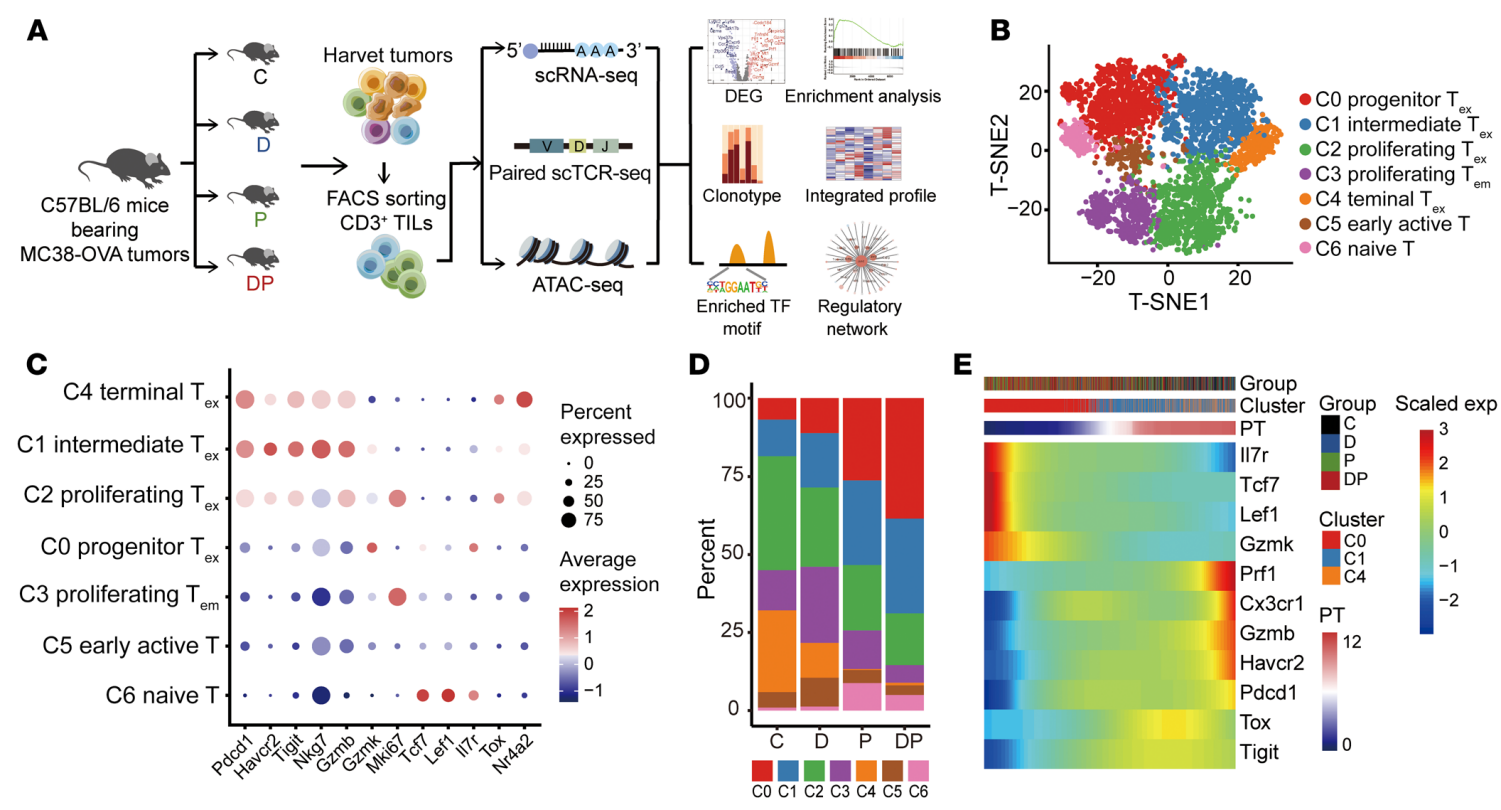
scRNA-Seq of tumor infiltrated T cells. (**A**) Graphical overview of the experimental setting. The scRNA-Seq, paired scTCR-Seq, and bulk ATAC-Seq were applied to sorted tumor infiltrated CD3^+^ T cells in C, D, P, and DP groups. Downstream analysis includes DEG, clonotype, enriched TF, regulatory network, GO, and GSEA enrichment analysis. (**B**) t-distributed stochastic neighbor embedding (t-SNE) plot showing CD8^+^ T cells. Each dot for a single cell, colored by unsupervised cluster. (**C**) Dot plot showing expression of selected marker genes per cell type. The size of the dot encodes the ratio of cells that expressed the genes, and its color encodes the average expression level. (**D**) Bar plot showing the subtype proportion of CD8^+^ T cells per group. (**E**) Gene expression dynamics along the CD8^+^ T_ex_ trajectory of cells in clusters 0, 1, and 4. PT, pseudotime; exp, expression.

To investigate how decitabine reprograms CD8^+^ T_ex_ and contributes to anti–PD-1–mediated rejuvenation in vivo at single-cell level, we isolated CD3^+^ TILs from MC38-OVA-bearing mice in the C group, D group, P group, or the DP group, as in Figure 3A, for droplet-based 5′ scRNA-Seq and paired scTCR-Seq (Figure 5A). According to our analysis, after quality control, 3,950 CD8^+^ T cells were collected and grouped into 7 clusters, among which, clusters 0, 1, 2, and 4 were identified as T_ex_ based on positive PD-1 expression; cluster 6 and 5 were identified as naive T cells and early active T cells; and cluster 2 and 3 were proliferating cells (Figure 5B, Supplemental Figure 8, and Supplemental Figure 9, A–D, see Methods). In addition, Cluster 0 was identified as progenitor T_ex_ for lower levels of *Pdcd1* (PD-1), *Havcr2* (TIM-3), *Tigit*, and *Nr4a2* and higher expression of *Gzmk* and *Tcf7* (TCF-1) (Figure 5C and Supplemental Figure 9E). The abundance of progenitor T_ex_ (cluster 0) was increased in the P group compared with the C or D group, reflecting their expansion after anti–PD-1 (Figure 5D, Supplemental Figure 9D, and Supplemental Figure 10A). Notably, consistent with our observation, the progenitor T_ex_ subset was more highly enriched in the DP group than in the P group. Contrastingly, the proportion of terminal T_ex_ (cluster 4) that had higher expression of known inhibitory molecules was most enriched in the C group (Figure 5, C and D and Supplemental Figure 10A). The trajectory of CD8^+^ T_ex_ (clusters 0, 1, 2, 4) suggested a possible path for CD8^+^ T cell exhaustion by Monocle method, with progenitor T_ex_ positioned at the root site and followed by intermediate T_ex_ (cluster 1) and terminal T_ex_ (cluster 4), while proliferative T_ex_ (cluster 2) were placed in another branch because of the impact of cell cycle stage (Supplemental Figure 10B), which was corroborated by the diffusion map and RNA velocity (Supplemental Figure 10, C–G). As expected, the memory markers (*Il7r*, *Tcf7*, and *Lef1*) decreased along pseudotime, while cytotoxicity genes (*Prf1*, *Gzmb*, and *Cx3cr1*) and exhaustion-related genes (*Pdcd1*, *Havcr2*, *Tigit*, and *Tox*) increased along the trajectory (Figure 5E). These results suggested decitabine pretreatment enhanced the expansion of anti–PD-1-responsive CD8^+^ progenitor T_ex_.

### Combination treatment of decitabine plus anti–PD-1 augments clonal expansion of progenitor T_ex_.

**Figure 6:**
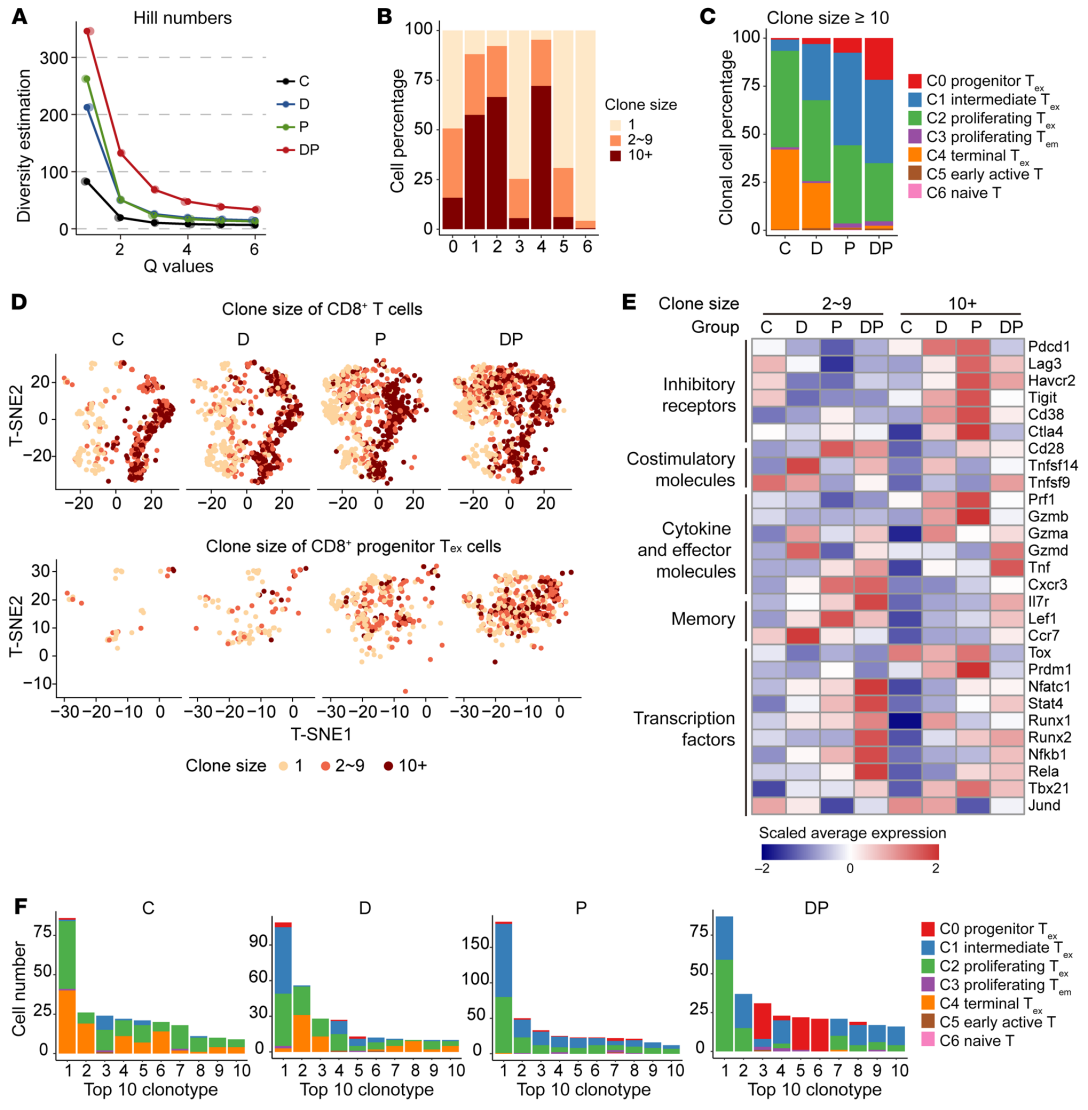
DP combination treatment augments clonal expansion of progenitor T_ex_. (**A**) Line chart showing sample diversity estimation using hill numbers, which were mathematically unified family of diversity indices. (**B**) Bar plot showing the cell percentage of each cluster stratified by clone size. The clone size were categorized as unique (*n* = 1), medium clonally expanded (2 ≤ *n* ≤ 9) and highly clonally expanded (*n* ≥ 10) based on the number of CD3^+^ T cells sharing the same TCRs. (**C**) Bar plot showing the subtype proportion of highly clonally expanded cells (cells with clone size ≥ 10) per group. (**D**) t-SNE plot of CD8^+^ T cells colored by clone size (top) and t-SNE plot of progenitor T_ex_ colored by clone size (bottom). (**E**) Heatmap showing the average expression levels of important marker genes of inhibitory receptors, costimulatory molecules, cytokines and effector molecules, memory and TFs for clonal cells in each group. (**F**) The cell numbers of top 10 clonotypes in each group.

We next analyzed clonotypes of TCR by using paired scRNA-Seq and scTCR-Seq data. A total of 1,857 unique TCR clonotypes were identified in the CD8^+^ T cells of the 4 groups, and 490 as expanding clonotypes that were represented by 2 cells or more, resulting in 2,583 clonal T cells. Firstly, we noticed that the DP group displayed the highest TCR diversity calculated using hill number and D50 diversity index, suggesting an active T cell anti-tumor immunity after DP treatment (Figure 6A and Supplemental Figure 11A). Moreover, the PD-1^+^ T_ex_ clusters (clusters 0, 1, 2, 4) had higher ratios and higher absolute numbers of clonal T cells and expanding clonotypes compared with PD-1^–^ non-T_ex_ clusters (Figure 6B, Supplemental Figure 11, B–D, and Supplemental Table 4), reflecting that T_ex_ clusters in our study were the main antitumor T cell population. Despite that the highest ratio of clonal T cells was detected in the C group, there was a significantly larger proportion of terminal T_ex_ (cluster 4) among the clonal T cells compared with other groups, while the clonal T cells in anti–PD-1 and DP groups were concentrated in the progenitor and intermediate T_ex_ subsets (Supplemental Figure 11, E–G). Remarkably, although decitabine reprogramed and enhanced the cytolytic capacity of TCR_OT-I_ T cells, after decitabine treatment in vivo,a considerable percentage of CD8^+^ TILs were not clonal cells, which implied that decitabine might activate some nonantitumor T cells, while the combination of decitabine plus anti–PD-1 precisely promoted the expansion of tumor-specific T cells.

We further investigated the distribution of medium clonally expanded (with 2 to 9 cells) and highly clonally expanded (with 10 and more cells) T subsets in these groups. Strikingly, DP combination yielded more highly clonally expanded cluster 0 progenitor T_ex_, while anti–PD-1 monotherapy caused abundant expansion of cluster 1 intermediate T_ex_, suggesting the preference for sustained progenitor T_ex_ expansion rather than further differentiation in DP group (Figure 6, C and D). In addition, the highly clonally expanded cells in the P group had higher expressions of inhibitory receptors (*Pdcd1*, *Lag3*, *Havcr2*, *Tigit*, and *Cd38*) and cytolytic molecules (*Prf1*, *Gzmb*) but lower levels of memory genes (*Il7r*, *Lef1*, and *Ccr7*) and effector gene *Tnf*, compared with those in the control group or the medium clonally expanded cells in the P group (Figure 6E). Importantly, we noticed that DP combination upregulated the expression of the functional genes (*Tnf*, *Gzma*, and *Gzmd*), memory genes (*Il7r*, *Lef1*, and *Ccr7*), and multiple crucial TFs (*Stat4*, *Runx1*, *Runx2*, *Nfkb1*, and *Jund*), but decreased the levels of inhibitory genes and exhaustion TFs (*Tox* and *Prdm1*) in highly clonally expanded cells, compared with those after anti–PD-1 monotherapy (Figure 6E). By analyzing differentially expressed genes (DEGs) between the highly clonally expanded T cells in the DP group and those in the P group, GO analysis revealed that the upregulated DEGs were associated with T cell differentiation, activation, and cell-cell adhesion, demonstrating the improved and durable T cell functionality among the highly clonally expanded cells (Supplemental Figure 11H and Supplemental Table 5).

Finally, we analyzed the frequency and phenotype of the most expanded TCR clonotypes. The total clone size of the top 10 TCR clonotypes was nearly 40% of the control group, and most of top 10 TCR clonotypes were terminal and proliferative T_ex_ (Figure 6F and Supplemental Figure 11I). Similarly, the terminal T_ex_ also were also a major component of decitabine-induced clonally expanding T cells. In contrast, among the top 10 TCR clonotypes, in both DP and P groups, few belonged to terminal T_ex_ (Figure 6F). Moreover, among the top 50 TCR clonotypes of all progenitor T_ex_, 58% of these clonotypes were from the DP group, including the top 5 clonotypes (Supplemental Figure 11J). Since the most frequent or highly expanding TCR clones might be tumor-recognizing T cells (20), DP treatment displayed superior ability to mediate the expansion of potential cancer-specific clonal progenitor T_ex_ compared with anti–PD-1 or decitabine single-agent therapy.

### Decitabine plus anti–PD-1 treatment reprograms the transcriptional and epigenetic profile of CD8^+^ progenitor T_ex_ with sustained activity of AP-1 family member JunD.

**Figure 7:**
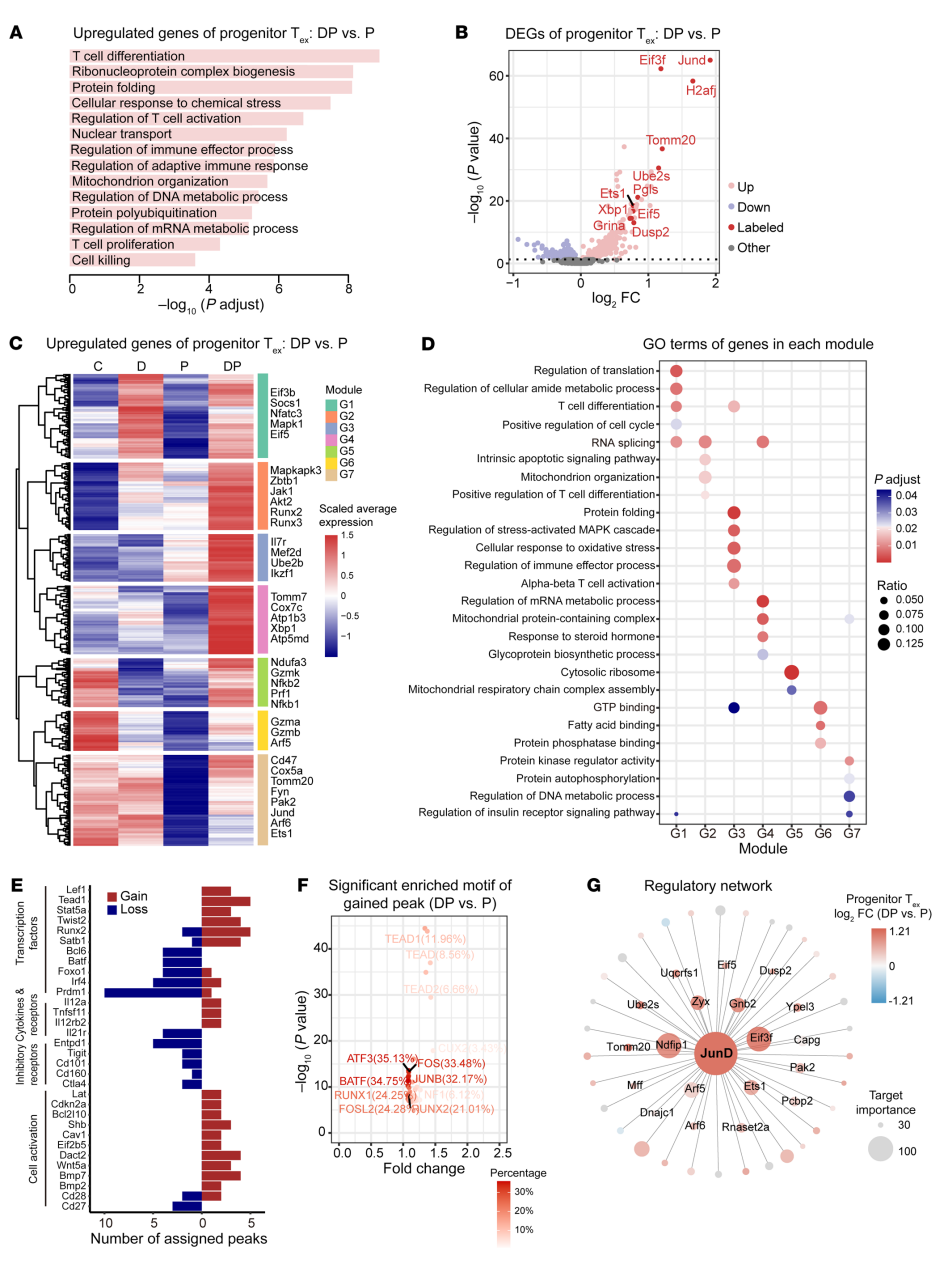
DP treatment reprograms transcriptional and epigenetic profile of CD8^+^ progenitor T_ex_ with sustained activity of AP-1 family member. (**A**) GO analysis of upregulated genes (688 genes, *P* < 0.05, 2-sided unpaired Wilcoxon test) for progenitor T_ex_ (C0) in DP group versus P group. Selected GO terms with Benjamini-Hochberg *P*_adj_ < 0.05. (**B**) Volcano plot showing DEGs of progenitor T_ex_ (C0) in DP group versus P group. Genes with *P* < 0.05 (2-sided unpaired Wilcoxon test) are colored and some important upregulated genes are labeled. (**C**) Heatmap showing the expression level of upregulated genes of DP group (versus P group) in progenitor T_ex_ cells for each group (same genes used in Figure 7A). The genes are clustered into 7 groups by hierarchical cluster analysis. (**D**) Dot plot showing GO terms of upregulated genes of each module as shown in Figure 7C. The size of the dot encodes the ratio of genes in each GO term, and its color encodes the Benjamini-Hochberg *P*_adj_ values. (**E**) Gene annotations of changed peaks between the DP versus P groups in CD3^+^ T cells. The numbers of differentially open gene regulatory regions for the indicated genes after DP combination versus P monotherapy are shown. (**F**) The significant enriched motifs of gained peaks between DP and P groups. Motifs of TFs with Benjamini *P*_adj_ < 0.05 (calculated by HOMER) are shown and important TFs are labeled. FC represents the ratio of the percentage of gained peaks with motif and the percentage of background peaks with motif. (**G**) Integrated transcriptional regulatory network inferred by SCENIC showing target genes of TF JunD whose importance are more than 30. Dot size represents the importance of target genes. Colors represent the log2 FC of averaged expression between the DP and P groups.

We next intended to define features of progenitor T_ex_ associated with DP treatment compared with those after anti–PD-1 monotherapy. We computed DEGs between cluster 0 progenitor T_ex_ from the DP group and P group. We identified 950 DEGs, including 688 upregulated genes and 262 downregulated genes. GO analysis showed that upregulated genes after DP treatment were enriched in T cell differentiation, regulation of T cell activation, regulation of immune effector processes, ribonucleoprotein complex biogenesis, and DNA/mRNA metabolic processes; additionally, *Jund* was most substantially increased with DP treatment compared with anti–PD-1 monotherapy (Figure 7, A and B and Supplemental Table 6). These 688 upregulated genes were clustered into 7 gene modules by hierarchical clustering. Genes in module 1 mainly induced by decitabine, such as *Socs1*, *Nfatc3*, *Mapk1*, and translation initiation factors *Eif5*, and *Eif3b*, which assisted the rapid biosynthesis and proliferation. Gene modules 2 and 3, consisted of *Mapkapk3*, *Jak1*, *Akt2*, *Runx2*, *Runx3*, *Mef2d*, and *Ube2d*, increased in the anti–PD-1 group compared with the control group, and further upregulated in the DP group, which contributed to enhanced T cell effector function. A third set of genes (gene modules 5, 6, and 7), included TFs *Jund*, *Ets1*, *Nfkb1*, and *Nfkb2*, effector genes *Prf1*, *Gzma*, and *Gzmk*, and mitochondrial metabolism related genes *Ndufa3*, *Cox5a*, and *Tomm20*, decreased after treated with anti–PD-1 but recovered when combined with decitabine (Figure 7C and Supplemental Table 6). GO and KEGG pathway analyses showed that decitabine treatment regulated T cell differentiation, while combination with anti–PD-1 dramatically increased T cell activation, TCR signaling, and MAPK signaling, and also regulated mitochondrial complex and metabolic processes (Figure 7D, Supplemental Figure 12A, and Supplemental Table 6). To explore the crucial TF for DP treatment, TF enriched analysis by matascape was applied and revealed that the upregulated genes in the combination group might be regulated by TP53, HDAC1, NFKB1, RELA, JUN, and MYCN (Supplemental Figure 12B).

We next examined mechanism underlying decitabine-mediated T cell reprogramming. An assay for transposase-accessible chromatin using sequencing (ATAC-Seq) of CD3^+^ TILs in C, P, and DP group showed that DP-treated T cells gained 6,730 peaks and lost 11,032 peaks compared with P group T cells (Supplemental Figure 12, C and D, see Methods). Whereas the absolute number of peaks was reduced after DP treatment, the width of peaks and overall ATAC-Seq signal within peak regions were increased (Supplemental Figure 12, D–F). To explore whether these changed peaks were related to genes of T cell function, we assigned these peaks to coding genes and found that genes involved in cytokine response, cell activation, and TFs such as *Lef1*, *Tead1*, *Stat5a*, and *Runx2* were more accessible after DP combination, whereas genes for inhibitory receptors (*Entpd1*, *Tigit*, *Cd101*, *Cd160*, and *Ctla4*),as well as *Bcl6, Prdm1, Irf4 and Batf* were more open after anti–PD-1 monotherapy (Figure 7E and Supplemental Table 7). The HOMER motif enrichment analysis revealed enrichment on DP-lost peaks (compared with the P group) for TF ELF4 (Supplemental Figure 12G), which induces cell cycle arrest in naive CD8^+^ T cells (21). Furthermore, DP-gained peaks (compared with the P group) were enriched for motifs of the Activating Protein 1 (AP-1) family (JunB, FOS, ATF3, FOSL2 and BATF), TEAD, RUNX1, and RUNX2), most of which were closed after anti–PD-1 monotherapy (compared with the C group) (Figure 7F and Supplemental Figure 12, H and I). Interestingly, *Jund* levels were significantly augmented after DP treatment compared with anti–PD-1, while other genes of these TFs showed minimal alteration (Supplemental Figure 12J).

Using the published JunD chromatin immunoprecipitation-Seq (ChIP-Seq) data in CD8^+^ T cells (22), we further demonstrated that open chromatin regions with JunD binding showed significantly increased chromatin accessibility in the DP group versus the P group, while other regions had little change (Supplemental Figure 13, A and B and Supplemental Table 8). We next identified the genes assigned to DP-gained peaks (compared with the P group) with JunD binding and found that these genes were enriched in biological processes such as lymphocyte differentiation, leukocyte cell-cell adhesion, and α-β T cell activation (Supplemental Figure 13C and Supplemental Table 8, see Methods). The transcriptional regulatory network inferred by SCENIC using scRNA-Seq data confirmed the increased expression of target genes and TF activity of JunD upon DP treatment versus anti–PD-1 in progenitor T_ex_ (Figure 7G, Supplemental Figure 13D, and Supplemental Table 9). JunD target levels and activity were decreased in the P group compared with the C group (Supplemental Figure 13, D and E). Since Jun family members regulate cell growth and survival, DP treatment resulted in higher proliferative capacity of progenitor T_ex_ than anti–PD-1 monotherapy via maintenance of JunD activity.

### Decitabine plus anti–PD-1 suppresses the terminal differentiation of T_ex_.

**Figure 8:**
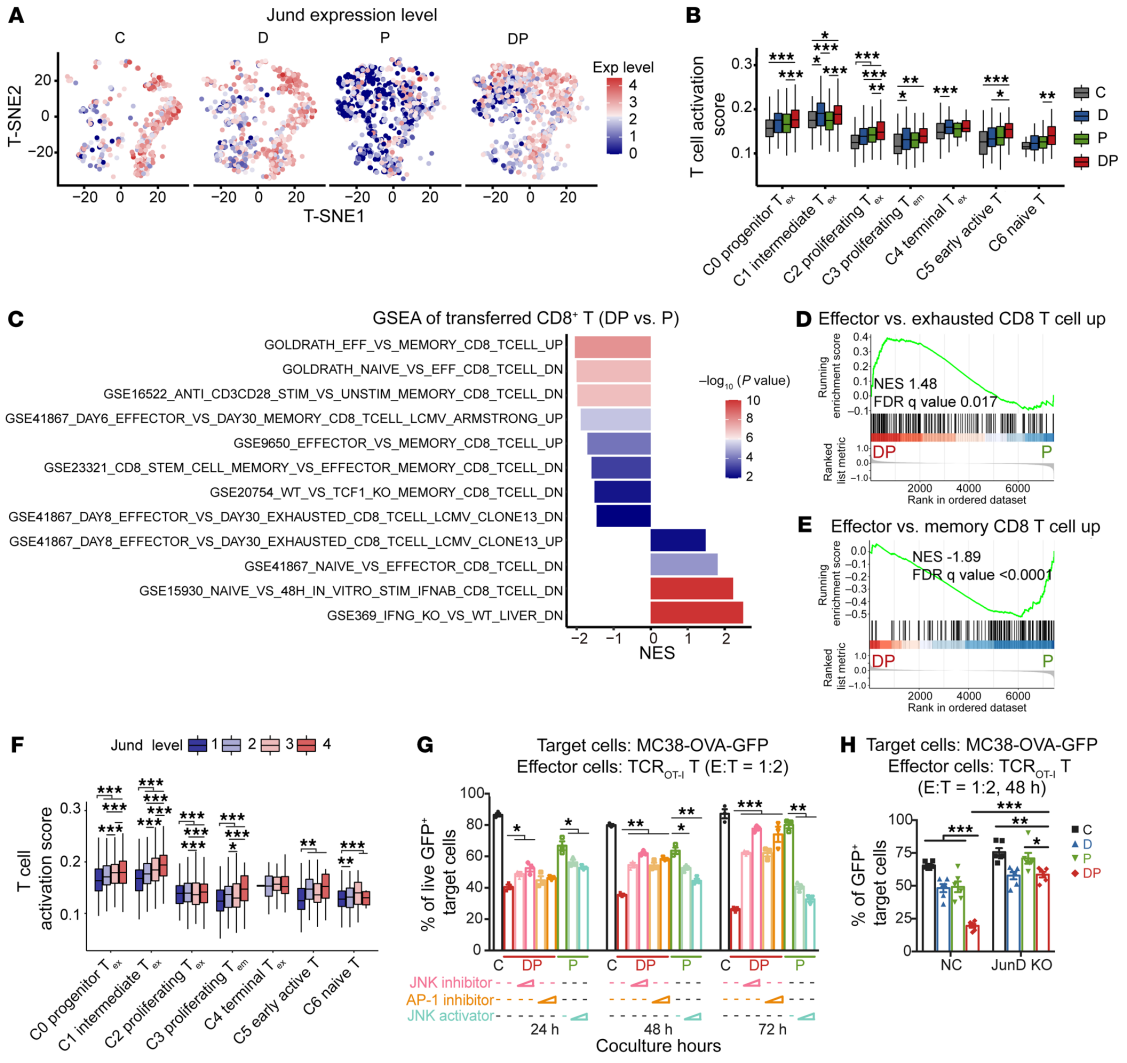
DP treatment suppresses the terminal differentiation of exhausted T cells. (**A**) t-SNE plot of CD8^+^ T cells colored by normalized expression of *Jund*. Exp, expression. (**B**) Boxplot showing T cell activation score calculated using the T cell activation (GO:0042110) gene set, 1-way ANOVA analysis. (**C**) Bar plot showing the NES from ranked list of genes expressed in proliferating T cells from ACT model in DP group and P group, calculated using GSEA. Gene signatures are from immunologic signature gene sets of MSigDB. (**D** and **E**) GSEA of indicated signatures (from GSE41867) from the ranked list of genes in proliferating T cells from ACT model in DP group versus P group. FDR, false discovery rate. (**F**) Boxplot showing T cell activation score from 4 groups of CD8^+^ TILs in cells with different JunD levels. Cells are divided into 4 groups, and groups 1, 2, 3, and 4 represent a quarter of cells with JunD expression levels from low to high. 1-way ANOVA analysis. (**G**) Purified naive CD8^+^ T cells from TCR_OT-I_ mice were activated, treated with PBS (C), anti–PD-1 (P), or decitabine plus anti–PD-1 (DP) as shown. Before coculture with MC38-OVA-GFP cells, the indicated T cells were incubated with JNK inhibitor JNK-IN-8 (50 nM, 100 nM), AP-1 inhibitor T-5224 (2 μM, 10 μM), or JNK activator anisomycin (0.5 μM, 1 μM) for 24 hours. These CD8^+^ T cells were then cocultured with MC38-OVA-GFP cells at an E-to-T ratio of 1:2. Frequencies of live GFP^+^ target cells are shown, by 1-way ANOVA analysis. (**H**) NC and JunD KO TCR_OT-I_ T cells were pretreated with decitabine, anti–PD-1, or the combination. Frequencies of live GFP^+^ MC38-OVA target cells after coculture with the indicated T cells at E-to-T ratio of 1:2 for 48 hours. 2-way ANOVA analysis. *P < 0.05; **P < 0.01; ***P < 0.001.

We noticed that the expression level of *Jund* was reduced in the P group compared with the C group but recovered in the DP group in all CD8^+^ T_ex_ clusters (Figure 8A and Supplemental Figure 14A). Analysis using human scRNA-Seq data from public data (23) also exhibited a decrease of *Jund* expression in CD8^+^ TILs following anti–PD-1-based immunotherapy in lung cancer patients (Supplemental Figure 14B). Moreover, immunofluorescence detection of tumor-infiltrated CD8^+^ T cells, as in the mouse model in Figure 3A, confirmed lower JunD protein levels in CD8^+^ TILs after anti–PD-1 treatment, which were upregulated after DP therapy (Supplemental Figure 14, C–F). In the in vitro tumor cell and T cell coculture system, TCR_OT-I_ T cells were sorted and consistent expression alteration of JunD was confirmed by quantitative real-time PCR assay (Supplemental Figure 14G). We then asked if decitabine priming could modulate the feature and activity of terminal T_ex_ after PD-1 blockade therapy.

T cell exhaustion is defined as limited proliferative ability, decreased production of effector genes, increased expression of inhibitory immune receptors and epigenetic alteration. Besides progenitor T_ex_, we also observed higher T cell activation scores with DP treatment compared with anti–PD-1 single-agent therapy in other CD8^+^ T_ex_ clusters (Figure 8B). Strikingly, the transcriptomes of CD8^+^ T_ex_ clusters in DP group revealed less exhaustion signatures compared with those in the anti–PD-1 group (Supplemental Figure 15, A and B). Moreover, with DP treatment, CD8^+^ naive T-differentiated terminal T_ex_, after persistent tumor antigen stimulation in vitro, produced more IFN-γ and TNF-α compared with anti–PD-1-treated terminal T_ex_ (Supplemental Figure 15C). Consistently, GSEA between adoptively transferred DP-treated CD8^+^ T cells and anti–PD-1-treated T cells revealed that DP-treated CD8^+^ T cells had higher expression of genes associated with memory and effector T cells, and lower levels of exhaustion genes (Figure 1I, Figure 8,C–E, Supplemental Figure 15D, and Supplemental Table 10). Epigenetic profiling also showed that DP-treated T cells displayed chromatin accessibility at some T cell activation genes associated with JunD binding (*Bcl2*, *Camk2d*, and *Stat4*) compared with anti–PD-1 monotherapy (Supplemental Figure 15, E–G). Collectively, besides activating progenitor T_ex_, DP treatment can also suppress the terminal differentiation of CD8^+^ T_ex_.

We next investigated whether AP-1/JunD signaling was involved in the activity of DP combination therapy on CD8^+^ T cells. First, we compared T cell activation score in CD8^+^ T cell clusters with different JunD levels. Notably, cells with higher *Jund* levels tended to have increased expression of genes associated with T cell activation, both from endogenous CD8^+^ TILs and transferred CD8^+^ T cells, suggesting the potential role of JunD in CD8^+^ T cells (Figure 8F and Supplemental Figure 15H). Secondly, we pretreated decitabine-primed CD8^+^ T cells with either inhibitor against AP-1 or upstream JNK1/2 and conducted the in vitro CD8^+^ TCR_OT-I_ cell-MC38-OVA coculture assay. Preventing JNK/AP-1 signaling in CD8^+^ T cells abolished DP treatment-induced T cell cytotoxicity (Figure 8G). Moreover, preincubation of CD8^+^ T cells with JNK activator anisomycin showed increased cytotoxicity and IFN-γ/TNF-α coproduction after PD-1 blockade (Figure 8G and Supplemental Figure 16A). Finally, to further validate the role of JunD, *Jund* was knocked out in TCR_OT-I_ T cells by CRISPR/Cas9 editing (Supplemental Figure 16, B and C). We observed that loss of JunD significantly repressed the proliferation ability of CD8^+^ T cells and disturbed the tumor-lysis capacity of CD8^+^ T cells following DP treatment (Figure 8H and Supplemental Figure 16, D–F). The frequency of IFN-γ^+^TNF-α^+^ cells was not markedly augmented in the DP group compared with anti–PD-1 treatment for JunD KO CD8^+^ T cells (Supplemental Figure 16G). Therefore, JunD downregulation after anti–PD-1 treatment could impair long-term T cell activity, and the enhanced antitumor strength of CD8^+^ T cells with DP combination was associated with JunD/AP-1 signaling.

Collectively, these data revealed crucial epigenetic and transcriptional changes in CD8^+^ T_ex_ following decitabine-plus-anti–PD-1 treatment. Epi-immunotherapy reprograms CD8^+^ T cells, promotes the activation and durable expansion of CD8^+^ progenitor T_ex_, and suppresses terminal differentiation. To investigate the clinical efficacy of DP therapy, we performed a clinical trial using low-dose decitabine and camrelizumab combination treatment in patients with solid tumors (ClinicalTrials.gov NCT02961101). Five patients with advanced gastrointestinal tumors (2 gastric cancers, 1 esophageal cancer, 1 colorectal cancer, and 1 breast cancer) who previously failed anti–PD-1 monotherapy completed in this study. Three patients acquired partial responses with the best reduction percentages in tumor burden being 80%, 90%, and 65%; 1 was evaluated as having stable disease and the other 1 had disease progression (Supplemental Figure 17 and Supplemental Table 11). These limited cases indicated that DP treatment had improved therapeutic outcomes in patients with advanced solid tumors that resisted anti–PD-1 monotherapy.

## Discussion

Diverse differentiation stages or subsets of T_ex_ possess distinct transcriptional profiles and epigenetic signatures, and PD-1/PD-L1 inhibitors mediated the expansion and transfer of progenitor T_ex_ into cytotoxic terminal T_ex_. We asked whether the addition of a DNA demethylation agent could maximize progenitor T_ex_ expansion and favor the sustained reinvigoration of T_ex_ treated by PD-1 blockade. Here, we show that low-dose combination therapy of decitabine plus anti–PD-1 markedly improved the magnitude and functionality of the clonally expanded progenitor T_ex_ subset and displayed robust antitumor potency.

Cancer-specific T cells are the desired ICB targets, along with a quantity of bystander TILs that recognize noncancer peptides infiltrated in tumors. As the cancer-recognizing TILs undergo numerous divisions upon TCR-dependent activation and acquire exhaustion phenotypes, the most frequent or highly expanded T_ex_ clones are identified as the cancer-specific T cells. Despite that a DNA demethylating agent can directly enhance activation and cytolytic capacity of CD8^+^ T cells, in this study, we observed that only a small proportion of CD8^+^ TILs belonged to tumor-specific clonal T cells after decitabine therapy; while the combination of decitabine and anti–PD-1 contributed to prominent clonal expansion of tumor-specific progenitor T_ex_. In addition, compared with anti–PD-1 monotherapy, decitabine plus anti–PD-1 resulted in elevated ratio and activation status of the highly expanded progenitor T_ex_ clonotypes and induced the most frequent progenitor T_ex_ clones. We also observed increased expressions of effector genes (*Tnf*, *Gzma*, and *Gzmd*) and key T cell activation TFs (*Nfatc1*, *Stat4*, *Runx1*, *Runx2*, and *Nfkb1*) in the medium expanded CD8^+^ T_ex_ after decitabine plus anti–PD-1 combination treatment, although it is still being investigated whether the active bystander TILs contributed to better tumor control with PD-1 blockade therapy. Another important issue was where the increased clonally expanding progenitor T_ex_ were derived from. First, the expanding progenitor T_ex_ might not be due to the reversible differentiation of terminal T_ex_ as DP therapy had minimal effects on the terminally exhausted T cells in vitro. Second, low-dose decitabine treatment broadened the TCR repertoire of CD8^+^ T cells, probably due to enhanced tumor immunogenicity. In combination with anti–PD-1, this might boost epitope spreading and, thus, the TCR diversity in the DP group was higher compared with the P group. The new expanding T_ex_ clones might come from the periphery, and further TCR-Seq in peripheral blood samples was needed. Third, it may arise from the expansion of preexisting progenitor T_ex_ in tumors.

The transition of progenitor T_ex_ into terminal T_ex_ was accompanied by wide transcriptional and epigenetic changes, during which dysregulation of TF nuclear factor of activated T cells (NFAT), AP-1, and increased expression of IRF4, NR4A, and TOX caused the imbalance between T cell activation and repression. AP-1 was a dimeric TF and the classic AP-1 heterodimer FOS-Jun induced IL-2 transcription as well as the inflammation memory-associated genes, while the AP-1-IRF4 complex drove the expression of exhaustion genes, and the terminal T_ex_ subset-specific open chromatin regions were enriched for interferon-related TF motifs (10, 24). Additionally, TCR activation following PD-1 inhibitors gave rise to partnerless NFAT lacking AP-1; NFAT interacted with NR4A and induced the transcription of exhaustion-associated genes (7, 9). In chimeric antigen receptor (CAR) T cells, overexpression of c-Jun enhanced cell expansion, increased functional activity, and declined terminal differentiation of CAR T cells (25). In our study, JunD expression and transcriptional activity in TILs was decreased after anti–PD-1 treatment, a similar alteration pattern was observed in scRNA data from patients with NSCLC. JunD controlled the expression of genes related to cell survival and metabolism and was essential in IL-7-induced proliferation of CD8^+^ T cells (26). Lower JunD levels in myeloid cells led to diminished binding of the JunD/FOS heterodimer to the *Tnf* promoter and thus decreased transactivation of the *Tnf* gene (27). Here, knockout of JunD in CD8^+^ T cells resulted in impaired proliferation and cytolysis activity, indicating the importance of JunD/AP-1 signaling in CD8^+^ T cells. Collectively, we proposed that deficiency in JunD-containing AP-1 complex formation might limit the self-renewal and effector function of T_ex_. Strikingly, in mouse tumor models, low-dose decitabine-primed CD8^+^ T_ex_ prevented the loss of JunD expression and decreased transcriptional activity following anti–PD-1 treatment, displaying stronger TCR-responsive and memory-like phenotypes, long-term proliferative capacity, and improved antitumor response. Further dynamic detection in tumor samples from patients in our clinical trials who received combination therapy with decitabine plus anti–PD-1 is particularly critical and is underway. Additionally, low-dose decitabine–modified TCR T cells or CAR T cells had superior tumor control, even in large tumor models, when combined with ICB, and will probably represent heightened clinical benefits.

The mechanistic insights into the epigenetic regulation of immune subpopulations, especially upon cancer immunotherapy, are complicated and multifaceted. Given that epigenetic modifications control immune cell differentiation, epigenetic interventions can modulate cell functions and reprogram cell commitment at earlier developmental stages as well as augment antitumor immunity (28). *DNMT3A* elicited the exhaustion-specific DNA methylation program, and conditional knockout of *Dnmt3a* in CD8^+^ effector T cells impacted T_ex_ differentiation state after viral infection (15). DNMT inhibitors directly enhanced the cytotoxic effects of CD8^+^ T cells and NK cells (16, 29). Consistently, our study revealed that DP therapy increased activation of progenitor CD8^+^ T_ex_. However, our study had several limitations. First, the epigenetic patterns of different CD8^+^ T_ex_ lineages upon DP therapy were not identified due to the lack of single cell ATAC-Seq. The chromatin accessibility alterations between distinct groups from our bulk ATAC-Seq might partially result from cell heterogeneity. The issue of why the absolute number of open chromatin regions was reduced after treatment with low-dose decitabine deserves further investigation. Additionally, although alterations of other immune populations in the tumor microenvironment were observed following DP therapy, such as CD4^+^ T cells (Supplemental Figure 18), the detailed regulation mechanism as well as the interplay between distinct immunocytes were undetermined. Future studies using single-cell multiomics sequencing — which enables joint profiling of chromatin accessibility, DNA methylation, and transcription in single cells, such as scNMT-Seq (30) — will better identify molecular mechanisms of the lost open-chromatin regions after decitabine treatment and the cooperation of DNA methylation and other epigenetic modifications on diverse immune cells treated by epi-immunotherapy.

In conclusion, our results show that decitabine plus anti–PD-1 treatment enhances antitumor response in multiple tumor models and significantly promotes the activation and expansion of CD8^+^ progenitor T_ex_. Notably, the AP-1/JunD signaling in CD8^+^ TILs was inactivated following PD-1 blockade therapy, while combination treatment with decitabine plus anti–PD-1 sustained the expression levels of JunD and target genes in CD8^+^ T cells from solid tumor models. Therefore, it is probable that patients with solid tumors who had decreased JunD expression in CD8^+^ TILs after anti–PD-1 monotherapy are the most likely to benefit from decitabine-plus-anti–PD-1 combination therapy.

## Methods

### Antibodies and reagents.

Anti–CD3ε-PerCP (100326), anti–CD8a-AF700 (100730), anti–CD8a-APC (100712), anti–PD-1-PE/DZ594 (109116), anti–Ki67-FITC (652410), anti–TCF1-PE (655208), anti–IFN-γ-BV421 (505830), anti–IFN-γ-PerCp/Cy5.5 (505822), anti–TNF-α-APC/Cy7 (506344), anti–TIM-3-PE/Cy7 (119716), anti–CD45-BV510 (103138), anti–Ly108 (Slamf6)-PE (134606), anti–PD-1-FITC (135214), anti–CD45.1-PE (110708), anti–CD45.2-AF488 (109816), purified anti-mouse CD3ε (100340), and purified anti-mouse CD28 (102116) were obtained from Biolegend. Anti–CD8-FITC (D271-4) and tetramer-SIINFEKL-APC (TS-5001-2c) were purchased from MBL. Cell Stimulation Cocktail Plus Protein Transport Inhibitors and FOXP3/TF Staining Buffer Set were bought from eBioscience. Naive CD8a^+^ T Cell Isolation and Tumor Dissociation Kits were obtained from Miltenyi Biotec.

### Cell lines and mouse models.

Murine colon carcinoma cell lines MC38-OVA and CT26 and T cell lymphoma cell line EG7-OVA were purchased from ATCC, and cells were cultured in RPMI 1640 supplemented with 10% FBS, 100 U/mL penicillin and 100 mg/mL streptomycin. C57BL/6J and Balb/c mice were obtained from SPF Biotechnology Co Ltd. C57BL/6J;CD45.1 mice were bought from Peking University Health Science Center Animal Science Department. OT-I mice were obtained from Jackson Laboratory. MC38-OVA cells (1.5 × 10^5^) and EG7-OVA cells (1 × 10^6^) were harvested and washed twice with PBS then injected s.c. into the right flank in 100 μL of PBS. When tumor volume was about 100 mm^3^, tumor-bearing mice were randomly assigned to receive PBS or decitabine (Sigma-Aldrich, 0.2 mg/kg/day) i.p. for 3 days. For the DP group, 2 days after decitabine treatment, anti–PD-1 antibody (Clone RMP1-14, Jiangsu Hengrui Pharmaceuticals Co Ltd, i.p. 200 μg per mouse) was administered every 3 days (2 or 4 doses). Tumor volume was estimated every other day and the tumor volume was calculated according to the formula (length × width^2^/2). For CD8^+^ or CD4^+^ T cell depletion, 200 μg anti-CD8 (Clone 2.43, BioXCell) or anti-CD4 (Clone GK1.5, BioXCell) was given i.p. twice weekly beginning 1 day prior to DP therapy.

### T cell isolation and in vitro treatment.

To isolate OVA-specific CD8^+^ T cells, spleens of OT-I transgenic mice (6–8 weeks old) were passed through a 70-μm nylon cell strainer (BD Falcon) and lymphocytes were isolated in mouse lymphocytes separation medium (Solarbio). Naive OVA-specific CD8^+^ T cells were purified using mouse naive CD8^+^ T Cell Isolation Kit according to the manufacturer’s instruction. Naive CD8^+^ T cells (> 90% purity) were seeded and activated in a 24-well plate bound with anti-CD3 (10 μg/mL) and anti-CD28 (10 μg/mL) antibodies, at a concentration of 3 × 10^6^ cells/mL, in RPMI 1640 medium supplemented with 10% FBS, 100 U/mL penicillin, 100 mg/mL streptomycin and recombinant IL-2 (200 U/mL) for 24 hours. After activation, CD8^+^ T cells were treated with PBS or 10 nM decitabine in a new plate. After 24-hour treatment, decitabine was removed with fresh medium, and CD8^+^ T cells were then treated with PBS or anti–PD-1 antibody (20 μg/mL) every 48 hours, and rIL-2 (200 U/mL) was added every other day. To isolate CD8^+^PD-1^+^Slamf6^+^TIM-3^–^ progenitor T_ex_ and CD8^+^PD-1^+^Slamf6^–^TIM-3^+^ terminal T_ex_, CD8^+^ T cells were collected and stained with CD8a, PD-1, Slamf6, and TIM-3 antibodies, followed by sorting on a Sony SH800S flow cytometry.

### In vitro killing assay.

MC38-OVA-GFP target cells were incubated with CD8^+^ T cells, progenitor, or terminal T_ex_ at the indicated E-to-T ratio in the presence of rIL-2 (200 U/mL). Initially, 1 × 10^4^ CD8^+^ T cells and 2 × 10^4^ MC38-OVA-GFP target cells (E-to-T=1:2) were mixed in 48-well flat-bottomed plate at day 0. At the indicated times of coincubation, CD8^+^ or GFP^+^ cell numbers were detected by flow cytometry.

### Mouse tumor tissue digestion and flow cytometry analysis.

To detect the phenotype and activity of TILs, tumor-bearing mice were sacrificed after the second dose of anti–PD-1. Tumor tissues were manually dissociated and minced into small pieces using scissors in 2 mL of cold RPMI 1640 medium without FBS. Shortly after, tumor pieces were digested enzymatically with Tumor Dissociation Kit and incubated at 37°C for 1 hour as described (11). After incubation, digested tumors were passed through a 70-μm nylon cell strainer and washed with cold PBS twice, then cells were suspended in RPMI 1640 medium without FBS. Single cell suspensions were used for flow cytometry. Surface staining was performed with the indicated antibodies for 15 minutes or Tetramer-SIINFEKL mAb for 45 minutes according to the manufacturer’s instructions. Intracellular proteins IFN-γ and TNF-α from TILs or T cells cocultured with tumor cells were detected after stimulation with cell stimulation cocktail plus protein transport inhibitors or Brefeldin A for 5 hours. Cells were first stained with surface antibodies, then fixed and permeabilized using Foxp3/TF Staining Buffer Set followed by intracellular antibody staining. After incubation, cells were washed and suspended in flow cytometry buffer and measured on DxFLEX (Beckman Coulter). Data analysis was performed using CytExpert and Kaluza software (Beckman Coulter).

### CyTOF Assay.

The tumor tissues were minced and dissociated into single cell suspensions using the Tissue Dissociation Kit. Cells were incubated with 0.25 μM cisplatin-194Pt at 4°C for 5 minutes, washed and incubated with block mix at 4°C for 20 minutes, followed by staining with a metal-conjugated surface antibody mix at 4°C for 30 minutes. Cells were then fixed and permeabilized with Foxp3/TF staining buffer and stained with DNA intercalator (0.25 μM iridium-191/193) at 4°C overnight. After washing, cells were incubated with metal-conjugated intracellular antibody mixture at 4°C for 30 minutes. The cells were then washed once in FACS and twice in ddH_2_O. Data were acquired using a CyTOF Helios mass cytometer (Fluidigm), normalized to EQ bead signal, debarcoded using a doublet filtering scheme, and analyzed using FlowJo v10.0.7. The metal-conjugated antibodies used in CyTOF assay are presented in Supplemental Table 1.

### Generation of JunD knockout TCR_OT-I_ T cells.

To generate JunD KO cells, the electroporation method was used. CRISPR/Cas9 gene editing was conducted by electroporation Cas9/gRNA (RNP) complex using 4D-Nucleofector System N (Lonza), Primary cell 4D-nucleofector kit (Lonza). Briefly, RNP containing 6 μg Cas9 protein and 6 μg sgRNA was precomplexed for 30 minutes at room temperature to create RNP complex as previously described (31). The mixture of RNP and activated TCR_OT-I_ T cells were transferred into the electroporation cuvette using the EO-115 program in 16-well cuvette strips. T cells were recovered in 200 μL preheated T cell medium and expanded as described above. Gene KO efficiency was detected using Tracking Indels by Decomposition, or TIDE. The JunD sgRNAs were sg1 CCGTCGGGGCGCAGCGCAGA, sg2 CGCTCGACGCACCCGCAGCC, sg3 GAGCGGCGGGATTGAAACCA, sg4 CGGGTAGAGGAACTGCGTAC, and sg5 GGATGGAAACGCCCTTCTAT, and sg2 was chosen in the functional experiments.

### T cell enrichment and scRNA-Seq.

To enrich the endogenous TILs, flow cytometry–sorting of live, CD45^+^CD3^+^ cells were performed on a BD Bioscience cell sorter. For the adoptive cell therapy assay, transferred live CD45.2^+^CD45.1^–^CD8^+^ T cells were sorted. Sorted cells were collected into cold PBS plus 2% FBS. The enriched T cells were immediately processed for scRNA-Seq library preparation. Cells were loaded between 10,000 and 15,000 cells /chip position using the 10 × Chromium Single Cell V(D)J Reagent Kit v1.1. The library was prepared according to the manufacture’s instructions.

### ScRNA-Seq data processing.

The scRNA-Seq reads were aligned to the mm10 mouse reference genome and quantified using cellranger count (10 × Genomics, v6.0.1). The scRNA-Seq quality control (QC) information is shown in Supplemental Table 12. The raw gene expression matrix from the cellranger pipeline was processed using the Seurat v4.0.6 (32). First, cells that had unique feature counts over 6,000 and less than 1,000 or had more than 5% mitochondrial counts were filtered, resulting in a count matrix of 17,885 cells and 18,227 genes. The IntegrateData function was used to correct for technical differences between data sets, accompanied by 2,000 highly variable features identification, data scaling, and linear dimensional reduction by principal component analysis (PCA). A K-nearest neighbor (KNN) graph was constructed based on the first 20 principals using the FindNeighbors function, followed by Louvain clustering using the FindClusters function at resolution 0.5 and a total of 13 clusters were identified. Finally, t-SNE was used to visualize the data sets (Supplemental Figure 8). The same pipeline was used on scRNA-Seq from the ACT assay.

### CD8^+^ T cell collection and clustering.

Since we focused on CD8^+^ cells, we selected cells in clusters 2, 5, and 7 (a total of 4,471 cells) that expressed Cd8a and Cd8b1 for further analysis (Supplemental Figure 8, A–D). We filtered 436 cells without paired TCR information. Then cells were reclustered and 9 subclusters were identified. Subclusters 7 and 8 were filtered out, because subcluster 7 cells expressed Cd4 and C8 had only 12 cells. Therefore, cells in clusters 0–6 (3,950 cells) were retained for further analysis (Figure 5B).

### Pseudotime and RNA velocity analysis.

Pseudotime was generated with Monocle v2.20.0 (33). The values specifying the mean-variance relationship were calculated by dispersionTable function, and high dispersion genes across cells with mean_expression ≥ 0.1 and dispersion_empirical ≥ 1 were selected for identifying cell subpopulations or ordering cells along a trajectory. The root of T_ex_ cell trajectory was defined as the location of progenitor T_ex_. The Destiny v3.4.0 (34) was used to visualize cells in a diffusion map. Velocyto.py v0.17.17 (35) run10 × was used to convert the bam files to loom files that contained the spliced, unspliced, and undefined matrix. The loom files were converted to Seurat object in Velocyto.R v0.6, which was also used to visualize the RNA velocity on t-SNE plot.

### Differential expression, GO, KEGG pathway, and GSEA.

To identify DEGs in clusters between 2 groups, we used the FindMarkers function in Seurat with Wilcoxon Rank Sum test as and Bonferroni’s correction. The thresholds for each set of DEGs and correction methods are shown in the figure legends. GO analysis was performed by clusterProfiler v4.0.5 (36) enrich GO function. KEGG pathway analysis was performed with Enrichr v3.0 (37) with databases=”KEGG_2019_Mouse”. The GSEA was performed by clusterProfiler v4.0.5 GSEA function. The average log_2_ (fold change) expression values were calculated by Seurat FindMarkers. Immunologic signature gene sets was obtained from MSigDB (https://www.gsea-msigdb.org/gsea/msigdb/).

### Gene regulatory network analysis.

The gene expression profiles of the CD8^+^ T cells were fed into pyscenic v0.11.2 (38). The grn, ctx, and aucell were used for derive coexpression modules from the expression matrix, find enriched motifs for a gene signature, and optionally prune targets from this signature and quantify the activity of gene signatures across single cells. In addition, SCENIC v1.2.4 R package was used for downstream analysis such as calculating the TF activity score and extracting regulatory network information. The regulatory network was visualized by Cytoscape v3.8.2 (39).

### ScTCR-Seq data processing.

TCR reads were aligned to the mm10 mouse reference genome and consensus TCR annotation was performed using cellranger vdj (10 × Genomics, v6.0.1). The scTCR-Seq QC information was shown in Supplemental Table 4. For each sample, the output file filtered_contig_annotations.csv, which contains TCRα- and β- chain CDR3 nucleotide sequences, was used for downstream analysis. The diversity estimation using hill number and D50 diversity index, and top clonal proportion were calculated by immunarch v0.6.8.

### Data availability.

Clinical study, ATAC-Seq data processing and analysis, WGBS data processing and analysis, and JunD ChIP-Seq data processing and analysis, and data availability are provided in Supplemental Methods.

### Statistics.

Experimental group assignment was determined by random designation. Data points represent biological replicates and are displayed as mean ± SEM. Statistical comparisons between 2 groups were analyzed using the 2-tailed Student’s t test or Wilcoxon test, and a 2-tailed *P* value < 0.05 was considered significant, as mentioned in the figure legends. ANOVA models were used to compare outcomes across multiple groups. The statistical relevance of survival was analyzed by the log-rank test. All statistical tests and correction methods are also shown in legends.

### Study approval.

All mice were housed under pathogen-free conditions in Chinese PLA General Hospital Laboratory Animal Centre (Beijing, China). All animal experiments and clinical studies were performed under protocols approved by Scientific Investigation Board of Chinese PLA General Hospital.

## Author contributions

JN, WH, and HC designed the experiments, interpreted the data, and composed the manuscript. XL conducted the in vitro and in vivo experiments. YL and XB analyzed the scRNA-Seq, ATAC-Seq and WGBS analysis. LD, YC, and XZ conducted parts of the mice experiment. CW and MC conducted the clinical trial. XL conducted the in vitro and in vivo mouse model experiments, while YL analyzed the scRNA-Seq data, determining order of co–first authorship.

## Supplementary Material

Supplemental data

Supplemental table 2

Supplemental table 3

Supplemental table 4

Supplemental table 5

Supplemental table 6

Supplemental table 7

Supplemental table 8

Supplemental table 9

Supplemental table 10

Supplemental table 12

## Version 1 02/28/2023

In-Press Preview

## Version 2 04/03/2023

Electronic publication
