# Supplementary material for: Decitabine priming increases anti–PD-1 antitumor efficacy by promoting CD8+ progenitor exhausted T cell expansion in tumor models
Source: J Clin Invest. 2023 Apr 3;133(7):e165673. doi: 10.1172/JCI165673 (PMC10065084; doi:10.1172/JCI165673)
Supplement: Supplemental data [file jci-133-165673-s107.pdf]

Supplemental information

Decitabine-priming increases anti-PD-1 antitumor efficacy by promoting CD8$^+$ progenitor exhausted T-cell expansion in tumor models

Xiang Li, Yaru Li, Liang Dong, Yixin Chang, Xingying Zhang, Chunmeng Wang, Meixia Chen, Xiaochen Bo, Hebing Chen, Weidong Han, Jing Nie

Contents

Supplemental Methods 3
Supplemental Figures 5
Figure S1. In vitro antitumor activity and DNA methylation analysis of decitabine-treated $CD8^+$ T-cells. 5
Figure S2. Phenotypes of endogenous immunocytes and transferred $CD8^+$ T-cells by CyTOF analysis. 7
Figure S3. Analysis of the transferred $CD8^+$ T-cells by scRNA-sequencing. 9
Figure S4. In vitro DP treatment on $CD8^+$ terminal T_{ex}. 11
Figure S5. Tumor microenvironment detection by CyTOF analysis after DP treatment. 13
Figure S6. The representative FACS plots on MC38-bearing mice model. 15
Figure S7. The effects of DP treatment on EG7-bearing and CT26-bearing mice models. 17
Figure S8. The expression profile of $CD3^+$ T-cells. 19
Figure S9. The quality control, cell distribution and expression level of $CD8^+$ T-cells. 21
Figure S10. The number, trajectory and RNA velocity of $CD8^+$ T-cells. 23
Figure S11. The paired TCR profiles of $CD8^+$ T-cells. 25
Figure S12. Integrated analysis of scRNA-seq and ATAC-seq. 27
Figure S13. Integrated analysis of scRNA-seq, ATAC-seq, and JunD ChIP-seq. 29
Figure S14. The expression level of JunD in this study and public data. 31
Figure S15. The transcriptional and epigenetic changes in $CD8^+$ T_{ex} following DP treatment. 33
Figure S16. Suppressed antitumor capacity of DP combination in JunD knockout $CD8^+$ T-cells. 35
Figure S17. Clinical trial of DP combination therapy in patients with advanced gastrointestinal tumors. 37
Figure S18. The effect of DP combination in $CD4^+$ T-cells. 39
Supplemental Tables 41
Table S1. Metal-conjugated antibodies used in CyTOF analysis. 41
Table S11. Patients baseline characteristics and clinical responses to decitabine-plus-camrelizumab combination therapy. 42

Supplemental Methods

Clinical study. To investigate the safety and efficacy of decitabine-plus-anti-PD-1 combination treatment, we conducted a phase II study at the Chinese People's Liberation Army General Hospital in patients with advanced solid tumors and lymphoma (NCT02961101). In this article, we report clinical results from patients with measurable histologically confirmed advanced solid tumors, who received decitabine (Jiangsu Chia Tai-Tianqing Pharmaceuticals Co. Ltd., 10 mg/d, days 1 to 5) plus anti-PD-1 antibody camrelizumab (Jiangsu Hengrui Pharmaceuticals Co. Ltd., 200 mg, day 8) combination therapy every 3 weeks until disease progression or unacceptable toxicity occurred, and appropriate chemotherapy (days 6 to 7) was used for patients with large tumor burdens. Patients provided written informed consent and study procedures were approved by the Institutional Review Board of the Chinese People's Liberation Army General Hospital (S2016-127-01). The primary efficacy end point was objective response rate (ORR), according to the Response Evaluation Criteria in Solid Tumors (RECIST1.1). Tumor assessment was performed at baseline and after every 6 weeks by CT/MRI. Adverse events were evaluated according to the NCI CTCAE v4.0.

ATAC-seq data processing and analysis. T-cells from the indicated conditions were collected and chromatin accessibility mapping performed by ATAC-seq, as described previously (40). The quality control assessment was performed by FastQC v0.11.9. Raw reads were trimmed using trim_galore v0.6.6 and aligned to the mm10 mouse reference genome using Bowtie2 v2.3.5.1. Duplicate reads were removed using picard MarkDuplicates v2.25.5 and only primary alignments with mapping quality greater than 30 were retained. Deeptools v3.5.1 computed the ATAC-seq signal in promoter regions ($\pm$ 1 kb around the transcription start site (TSS)) and then the pearson correlation among samples was calculated. ATAC-seq peaks were called using MACS2 v 2.2.7.1 on each individual sample. Peaks that present in both replicates are preserved for downstream analysis. If the peak in one sample does not overlap in another sample, then it is identified as "gain" peak, and vice versa as "loss" peak. Homer v4.11 findMotifsGenome.pl was used to find enriched motifs in "gain" or "loss" peaks.

WGBS data processing and analysis. The quality control assessment was performed by FastQC v0.11.9. Raw reads were trimmed using trim_galore v0.6.6 and aligned to the mm10 mouse reference genome using bsmap v2.90. Duplicate reads were removed using sambamba v.0.6.8. Differentially methylated regions (DMRs) between

samples were identified using DSS v2.40.0 (41) with delta = 0.1 and p.threshold = 0.05.

JunD ChIP-seq Data Processing and Analysis. JunD ChIP-seq data were from Gene Expression Omnibus database under accession codes GSE77857. Raw reads were aligned to the mm10 mouse reference genome using Bowtie2 v2.3.5.1. Duplicate reads were removed using picard MarkDuplicates v2.25.5 and only primary alignments with mapping quality greater than 30 were retained. ChIP-seq peaks were called using MACS2 v 2.2.7.1 on each individual sample. There were 342 JunD ChIP-seq peaks overlapping with DP-gained peaks (versus P group) and 326 genes were assigned to these peaks using clusterProfiler v4.0.5.

Data availability. The endogenous and transferred T cell scRNA-seq and ATAC-seq data are available in the Gene Expression Omnibus database under accession codes GSE209965.

References

40. Buenrostro JD, Giresi PG, Zaba LC, Chang HY, and Greenleaf WJ. Transposition of native chromatin for fast and sensitive epigenomic profiling of open chromatin, DNA-binding proteins and nucleosome position. Nature methods. 2013;10(12):1213-8.

41. Wu H, Xu TL, Feng H, Chen L, Li B, Yao B, et al. Detection of differentially methylated regions from whole-genome bisulfite sequencing data without replicates. Nucleic Acids Research. 2015;43(21).

Supplemental Figures

Figure S1. In vitro antitumor activity and DNA methylation analysis of decitabine-treated $CD8^+$ T-cells.

(**A**) Absolute number of GFP^+ target cells during the co-incubation of MC38-OVA-GFP and $CD8^+$ T-cells from OT-I mice at the indicated times. Results are pooled from 2 experiments with n=6 per group. The representative FACS plots for GFP^+ target cells and their total numbers are shown. Two-way ANOVA analysis. (**B**) Frequency of live GFP^+ MC38 cells after 4 days of co-culture with TCR_{OT-I} $CD8^+$ T-cells at the indicated E: T ratios (n=3). One-way ANOVA analysis. (**C-D**) Frequency of $Ki67^+$ cells (**C**) or IFN-γ^+TNF-α^+ cells (**D**) in $CD8^+$ T-cells during the co-incubation of MC38-OVA and TCR_{OT-I} $CD8^+$ T-cells at the indicated times, gated on $CD8^+$ cells (n=6). The representative FACS plots and frequencies are shown. (**E-G**) Splenocytes isolated from OT-I mice were stimulated with $OVA_{257-264}$ for 2 days with rIL-2, cells were cultured in medium containing rIL-2 for another 2 days. Cells were treated with decitabine, anti-PD-1, or combination and co-cultured with MC38-OVA-GFP or MC38-OVA cells as Figure 1A. Absolute number of $CD8^+$ cells (**E**) and frequency of live GFP^+ MC38 cells (**F**) after 2 days of co-culture at E: T ratio of 1:4 are shown (n=6). (**G**) Frequency of IFN-γ^+TNF-α^+ cells in $CD8^+$ T-cells. The representative FACS plots and frequencies are shown (n=3). (**H**) Violin plot with included boxplot showing that methylation level in promoter region (±1 kb around the transcription start site (TSS)); two-sided unpaired wilcox test. C group, PBS-treated control T-cells; D group, 10 nM decitabine-treated T-cells. (**I**) Heatmap showing methylation level in downregulated differentially methylated region (DMR) in D group versus C group. (**J**) Bar plot showing the distribution of downregulated DMR. (**K**) KEGG terms of genes whose promoter lied in downregulated DMR. Bar plots represent the mean ± SEM. * $p < 0.05$; ** $p < 0.01$; *** $p < 0.001$, by one-way ANOVA analysis.

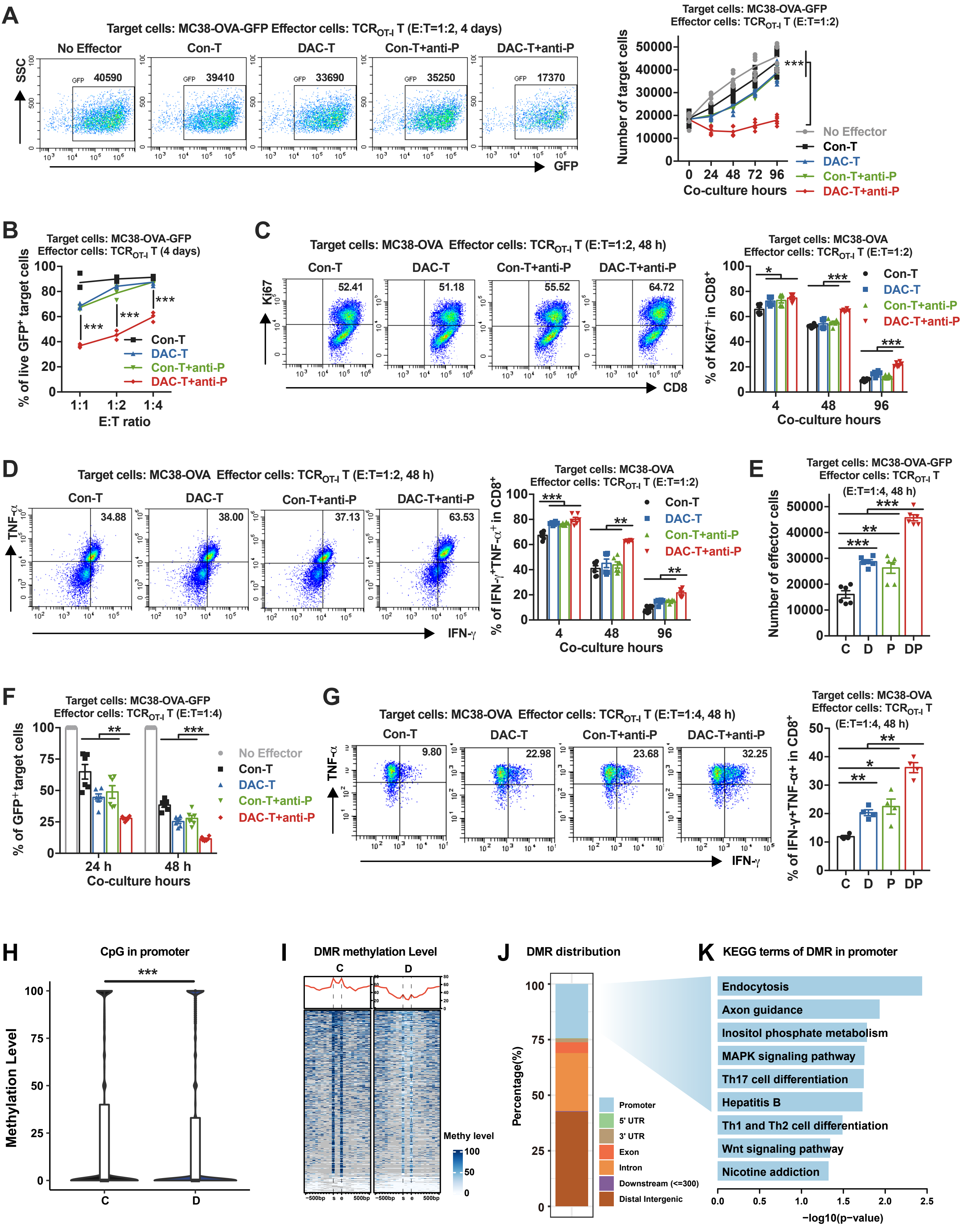

A
Target cells: MC38-OVA-GFP Effector cells: TCR$_{OT-I}$ T (E:T=1:2, 4 days)
No Effector
Con-T
DAC-T
Con-T+anti-P
DAC-T+anti-P
SSC
GFP
40590
39410
33690
35250
17370
Target cells: MC38-OVA-GFP
Effector cells: TCR$_{OT-I}$ T (E:T=1:2)
Number of target cells
Co-culture hours
***
No Effector
Con-T
DAC-T
Con-T+anti-P
DAC-T+anti-P
B
Target cells: MC38-OVA-GFP
Effector cells: TCR$_{OT-I}$ T (4 days)
% of live GFP$^+$ target cells
E:T ratio
1:1
1:2
1:4
***
Con-T
DAC-T
Con-T+anti-P
DAC-T+anti-P
C
Target cells: MC38-OVA Effector cells: TCR$_{OT-I}$ T (E:T=1:2, 48 h)
Con-T
DAC-T
Con-T+anti-P
DAC-T+anti-P
Ki67
CD8
52.41
51.18
55.52
64.72
Target cells: MC38-OVA
Effector cells: TCR$_{OT-I}$ T (E:T=1:2)
% of Ki67$^+$ in CD8$^+$
Co-culture hours
*
***
Con-T
DAC-T
Con-T+anti-P
DAC-T+anti-P
D
Target cells: MC38-OVA Effector cells: TCR$_{OT-I}$ T (E:T=1:2, 48 h)
Con-T
DAC-T
Con-T+anti-P
DAC-T+anti-P
TNF-α
IFN-γ
34.88
38.00
37.13
63.53
Target cells: MC38-OVA
Effector cells: TCR$_{OT-I}$ T (E:T=1:2)
% of IFN-γ$^+$TNF-α$^+$ in CD8$^+$
Co-culture hours
***
**
Con-T
DAC-T
Con-T+anti-P
DAC-T+anti-P
E
Target cells: MC38-OVA-GFP
Effector cells: TCR$_{OT-I}$ T
(E:T=1:4, 48 h)
Number of effector cells
***
**
C
D
P
DP
F
Target cells: MC38-OVA-GFP
Effector cells: TCR$_{OT-I}$ T (E:T=1:4)
% of GFP$^+$target cells
**
***
24 h
48 h
Co-culture hours
No Effector
Con-T
DAC-T
Con-T+anti-P
DAC-T+anti-P
G
Target cells: MC38-OVA Effector cells: TCR$_{OT-I}$ T (E:T=1:4, 48 h)
Con-T
DAC-T
Con-T+anti-P
DAC-T+anti-P
TNF-α
IFN-γ
9.80
22.98
23.68
32.25
Target cells: MC38-OVA
Effector cells: TCR$_{OT-I}$ T
(E:T=1:4, 48 h)
% of IFN-γ+TNF-α+ in CD8$^+$
**
*
C
D
P
DP
H
CpG in promoter
***
Methylation Level
C
D
I
DMR methylation Level
C
D
Methy level
-500bp
s
e
500bp
J
DMR distribution
Percentage(%)
Promoter
5' UTR
3' UTR
Exon
Intron
Downstream (<=300)
Distal Intergenic
K
KEGG terms of DMR in promoter
Endocytosis
Axon guidance
Inositol phosphate metabolism
MAPK signaling pathway
Th17 cell differentiation
Hepatitis B
Th1 and Th2 cell differentiation
Wnt signaling pathway
Nicotine addiction
−log10(p−value)

Figure S2. Phenotypes of endogenous immunocytes and transferred $CD8^+$ T-cells by CyTOF analysis.

(**A**) CyTOF mapping of tumor-infiltrated $CD45^+$ cells. On the left is a t-distributed stochastic neighbor embedding (t-SNE) visualization of the annotated immunocytes. On the right is a bar plot showing the proportions of immune subsets of each group. (**B**) Heatmap showing expression of exhaustion genes in transferred $CD45.2^+CD8^+$ T-cells. (**C**) t-SNE plots for marker gene expression of T cell phenotypes.

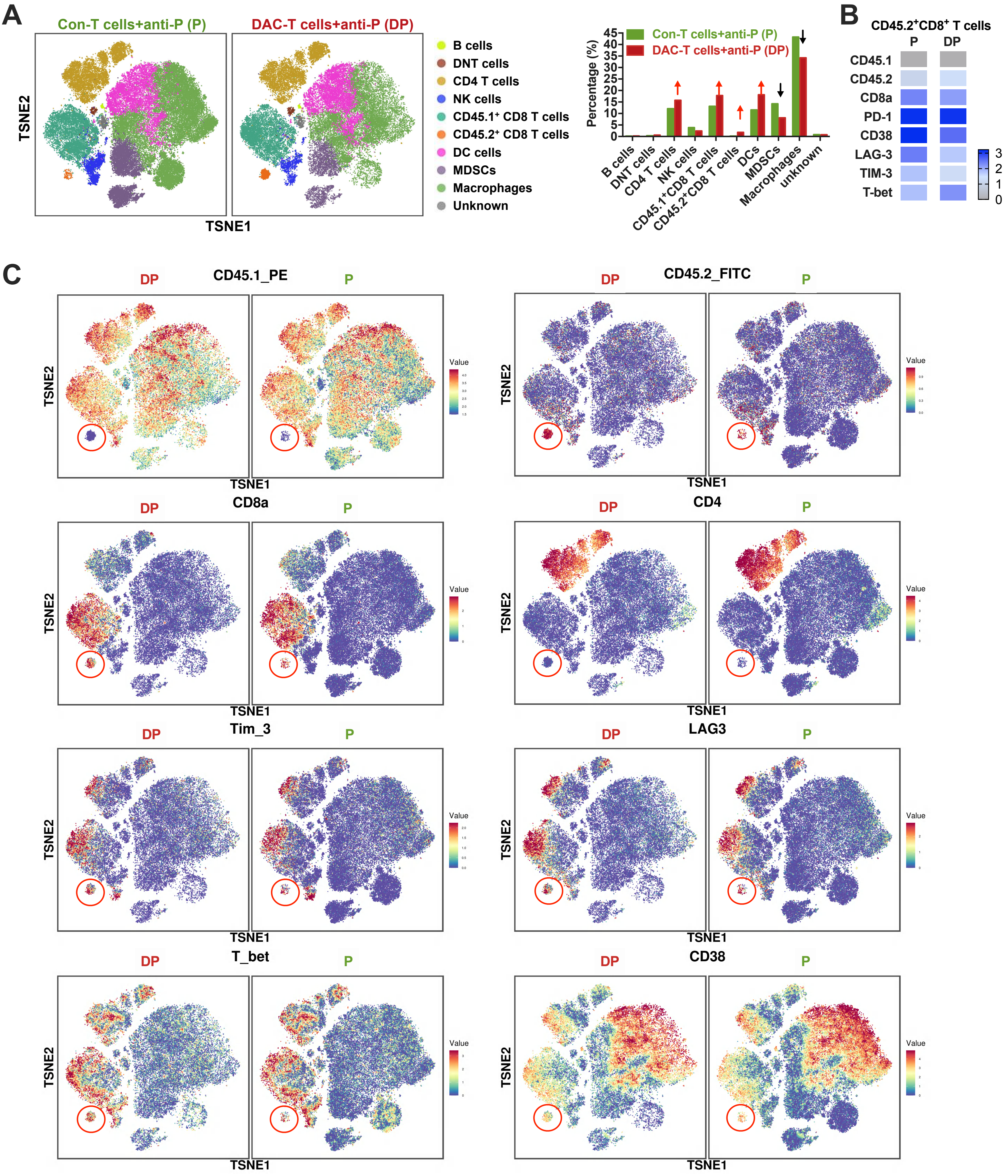

A
Con-T cells+anti-P (P)
DAC-T cells+anti-P (DP)
TSNE2
TSNE1
B cells
DNT cells
CD4 T cells
NK cells
CD45.1⁺ CD8 T cells
CD45.2⁺ CD8 T cells
DC cells
MDSCs
Macrophages
Unknown
Percentage (%)
Con-T cells+anti-P (P)
DAC-T cells+anti-P (DP)
B cells
DNT cells
CD4 T cells
NK cells
CD45.1⁺CD8 T cells
CD45.2⁺CD8 T cells
DCs
MDSCs
Macrophages
unknown
B
CD45.2⁺CD8⁺ T cells
P
DP
CD45.1
CD45.2
CD8a
PD-1
CD38
LAG-3
TIM-3
T-bet
C
CD45.1_PE
CD45.2_FITC
CD8a
CD4
Tim_3
LAG3
T_bet
CD38
DP
P
Value

Figure S3. Analysis of the transferred CD8$^+$ T-cells by scRNA-sequencing.

(**A**) t-SNE plot of the transferred CD45.2$^+$ CD8$^+$ TCR$_{OT-1}$ T-cells colored by normalized expression of each gene. (**B-C**) t-SNE plot of the transferred CD45.2$^+$ CD8$^+$ T-cells. Each dot indicates a single cell, colored by clonotype (**B**) and cell cycle (**C**). (**D-E**) t-SNE plot of the transferred CD45.2$^+$ CD8$^+$ T-cells separated by sample. Each dot indicates a single cell, colored by cluster (**D**) and group (**E**) respectively. (**F**) Bar plot showing the subtype proportion of CD8$^+$ T-cells per group. Fisher exact test, *** $p < 0.001$. (**G**) Volcano plot showing DEGs for proliferating T-cells in anti-PD-1 (P) group versus control (C) group. Genes with p.adjust < 0.05 (two-sided unpaired wilcox test, Bonferroni correction adjusted) and absolute log2 (fold change) ⩾ 0.2 are identified as DEGs. In addition, genes with p.adjust < 0.05 and absolute log2 (fold change) ⩾ 0.5 are labeled. (**H**) Gene Ontology (GO) analysis of the upregulated genes (P vs C) in proliferating T-cells. Selected GO terms with Benjamini-Hochberg adjusted p values <0.05. (**I**) GO analysis of the upregulated genes (DP vs P) in proliferating T-cells. Selected GO terms with Benjamini-Hochberg adjusted p values < 0.05. (**J**) Volcano plots showing DEGs for non-proliferating T cells in decitabine-plus-anti-PD-1 (DP) group versus anti-PD-1 (P) group. Genes with p.adjust < 0.05 (two-sided unpaired wilcox test, Bonferroni correction adjusted) and absolute log2 (fold change) ⩾ 0.2 are identified as DEGs. In addition, genes with p.adjust < 0.05 and absolute log2 (fold change) ⩾ 0.5 are labeled. (**K**) Venn plot showing the DEGs of upregulated genes in proliferating T-cells and non-proliferating T-cells respectively.

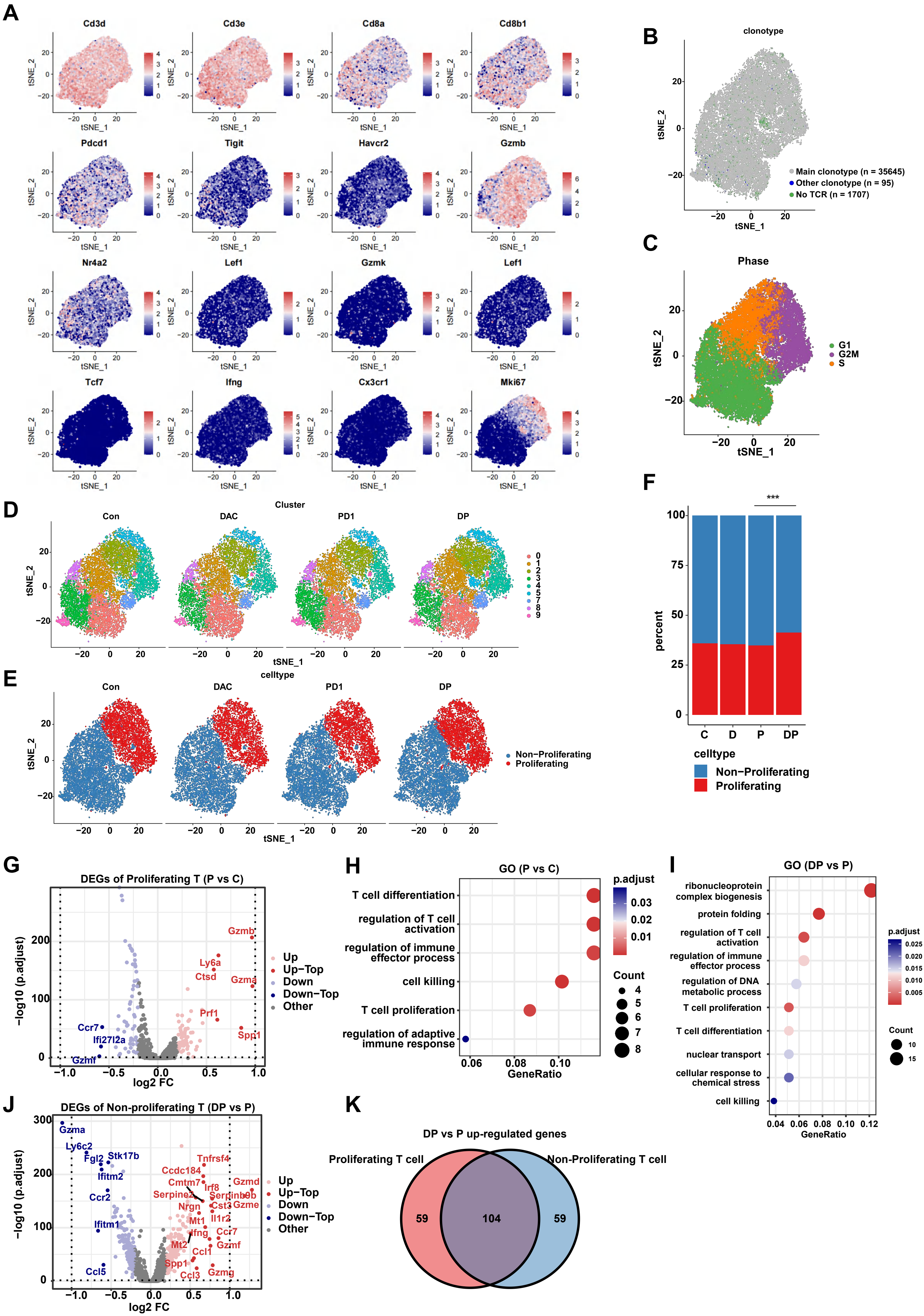
A
Cd3d
Cd3e
Cd8a
Cd8b1
Pdcd1
Tigit
Havcr2
Gzmb
Nr4a2
Lef1
Gzmk
Lef1
Tcf7
Ifng
Cx3cr1
Mki67
tSNE_1
tSNE_2
B
clonotype
Main clonotype (n = 35645)
Other clonotype (n = 95)
No TCR (n = 1707)
C
Phase
G1
G2M
S
D
Cluster
Con
DAC
PD1
DP
0
1
2
3
4
5
7
8
9
E
celltype
Non−Proliferating
Proliferating
F
***
percent
C
D
P
DP
celltype
Non−Proliferating
Proliferating
G
DEGs of Proliferating T (P vs C)
−log10 (p.adjust)
log2 FC
Gzmb
Ly6a
Ctsd
Gzma
Prf1
Spp1
Ccr7
Ifi27l2a
Gzmf
Up
Up−Top
Down
Down−Top
Other
H
GO (P vs C)
T cell differentiation
regulation of T cell activation
regulation of immune effector process
cell killing
T cell proliferation
regulation of adaptive immune response
GeneRatio
p.adjust
Count
I
GO (DP vs P)
ribonucleoprotein complex biogenesis
protein folding
regulation of T cell activation
regulation of immune effector process
regulation of DNA metabolic process
T cell proliferation
T cell differentiation
nuclear transport
cellular response to chemical stress
cell killing
GeneRatio
p.adjust
Count
J
DEGs of Non-proliferating T (DP vs P)
−log10 (p.adjust)
log2 FC
Gzma
Ly6c2
Fgl2
Stk17b
Ifitm2
Ccr2
Ifitm1
Ccl5
Tnfrsf4
Ccdc184
Cmtm7
Irf8
Gzmd
Serpine2
Serpinb9b
Nrgn
Cst3
Gzme
Mt1
Il1r2
Ifng
Ccr7
Mt2
Gzmf
Ccl1
Spp1
Ccl3
Gzmg
Up
Up−Top
Down
Down−Top
Other
K
DP vs P up-regulated genes
Proliferating T cell
Non-Proliferating T cell
59
104
59

Figure S4. In vitro DP treatment on CD8$^+$ terminal T$_{ex}$.

(**A**) Absolute number of CD8$^+$ T-cells co-cultured with MC38-OVA-GFP cells at E: T of 1:2 for the indicated times (n=3). The representative FACS plots for CD8$^+$ effector T-cells with their total numbers are shown. Two-way ANOVA analysis. (**B**) Representative FACS plots for Ki67$^+$ cells and their frequencies in CD8$^+$ TCR$_{OT\text{-}1}$ T cells with MC38-OVA cells at E: T ratio of 1:2. (**C-D**) Frequency of TCF-1$^+$TIM-3$^-$PD-1$^+$ progenitor T$_{ex}$ (**C**) and TCF-1$^-$TIM-3$^+$PD-1$^+$ terminal T$_{ex}$ (**D**) at the indicated times of TCR$_{OT\text{-}I}$ T-cells co-culture with MC38-OVA cells. Two-way ANOVA analysis. (**E**) Experimental timeline (left) and frequency of Ki67$^+$ cells (right) in CD8$^+$ T cells at 2 day-co-culture of differentiated CD8$^+$ T-cells with MC38-OVA cells at E: T ratio of 1:2. One-way ANOVA analysis. (**F-G**) Absolute numbers of target cells were calculated during co-culture with Slamf6$^+$TIM-3$^-$ progenitor T$_{ex}$ (**F**) or Slamf6$^-$TIM-3$^+$ terminal T$_{ex}$ (**G**) at the indicated times (n=6). The representative FACS plots for Slamf6$^+$TIM-3$^-$PD-1$^+$CD8$^+$ effector cells and GFP$^+$ target cells as well as their total numbers are shown. Two-way ANOVA analysis. * $p < 0.05$; ** $p < 0.01$; *** $p < 0.001$.

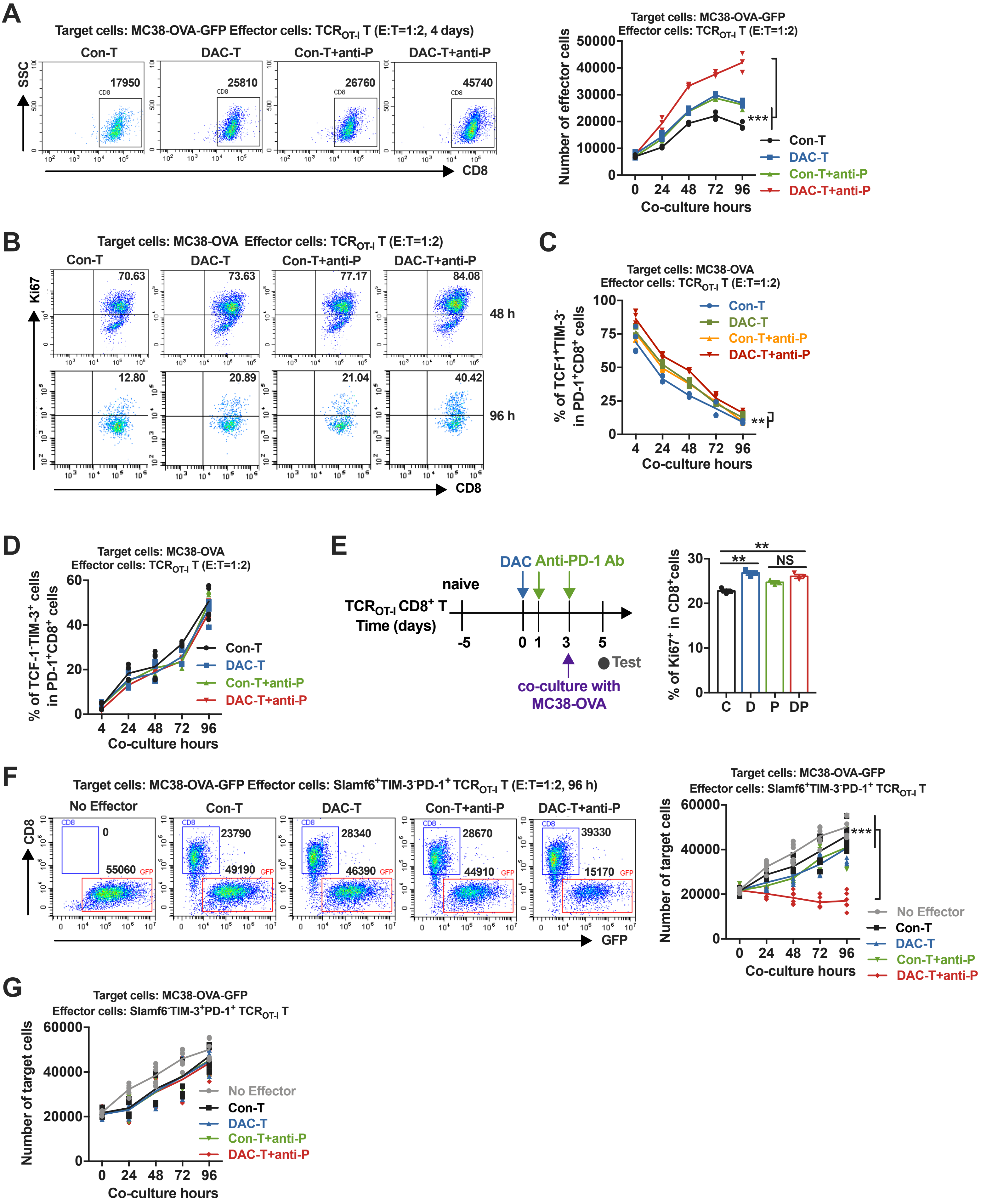
A
Target cells: MC38-OVA-GFP Effector cells: TCROT-I T (E:T=1:2, 4 days)
Con-T
DAC-T
Con-T+anti-P
DAC-T+anti-P
SSC
CD8
17950
25810
26760
45740
Target cells: MC38-OVA-GFP
Effector cells: TCROT-I T (E:T=1:2)
Number of effector cells
Co-culture hours
***
Con-T
DAC-T
Con-T+anti-P
DAC-T+anti-P
B
Target cells: MC38-OVA Effector cells: TCROT-I T (E:T=1:2)
Ki67
CD8
70.63
73.63
77.17
84.08
48 h
12.80
20.89
21.04
40.42
96 h
C
Target cells: MC38-OVA
Effector cells: TCROT-I T (E:T=1:2)
% of TCF1+TIM-3- in PD-1+CD8+ cells
Co-culture hours
**
D
Target cells: MC38-OVA
Effector cells: TCROT-I T (E:T=1:2)
% of TCF-1-TIM-3+ cells in PD-1+CD8+ cells
Co-culture hours
E
naive
TCROT-I CD8+ T
Time (days)
DAC
Anti-PD-1 Ab
Test
co-culture with
MC38-OVA
% of Ki67+ in CD8+cells
**
**
NS
C
D
P
DP
F
Target cells: MC38-OVA-GFP Effector cells: Slamf6+TIM-3-PD-1+ TCROT-I T (E:T=1:2, 96 h)
No Effector
Con-T
DAC-T
Con-T+anti-P
DAC-T+anti-P
CD8
GFP
0
55060
23790
49190
28340
46390
28670
44910
39330
15170
Target cells: MC38-OVA-GFP
Effector cells: Slamf6+TIM-3-PD-1+ TCROT-I T
Number of target cells
Co-culture hours
***
No Effector
G
Target cells: MC38-OVA-GFP
Effector cells: Slamf6-TIM-3+PD-1+ TCROT-I T
Number of target cells
Co-culture hours

Figure S5. Tumor microenvironment detection by CyTOF analysis after DP treatment.

(**A**) Tumor-free, decitabine-plus-anti-PD-1-treated mice (n=8) were rechallenged with 3×10^5 MC38-OVA cells, 2 times the original number of tumor cells, on day 60 at least 1 month after complete elimination of primary tumors. (**B**) Balb/c mice were transplanted with 1×10^6 CT26 cells, treated with PBS (C group; black), or decitabine alone (0.2 mg/kg per mouse, days 7-9; D group, blue), or anti-PD-1 antibody alone (200 μg per mouse, days 12, 15, 18, 21; P group, green) or decitabine plus anti-PD-1 (DP group, red) as indicated. The average tumor growth curves of each treatment group are shown. * $p < 0.05$ by two-way ANOVA analysis. (**C**) CyTOF mapping of tumor-infiltrated $CD45^+$ cells. Shown is t-SNE visualization, colored by treatment group. (**D**) t-SNE plot for marker gene expression of distinct immune cell phenotypes. (**E**) Heatmap showing marker gene expression values. (**F-G**) IFN-γ, IL-6 and TNF-α secretion in response to OVA presentation by BMDCs pretreated with decitabine (0, 1nM, 10nM, 100nM) to $CD4^+$ (**F**) and $CD8^+$ (**G**) T-cells.

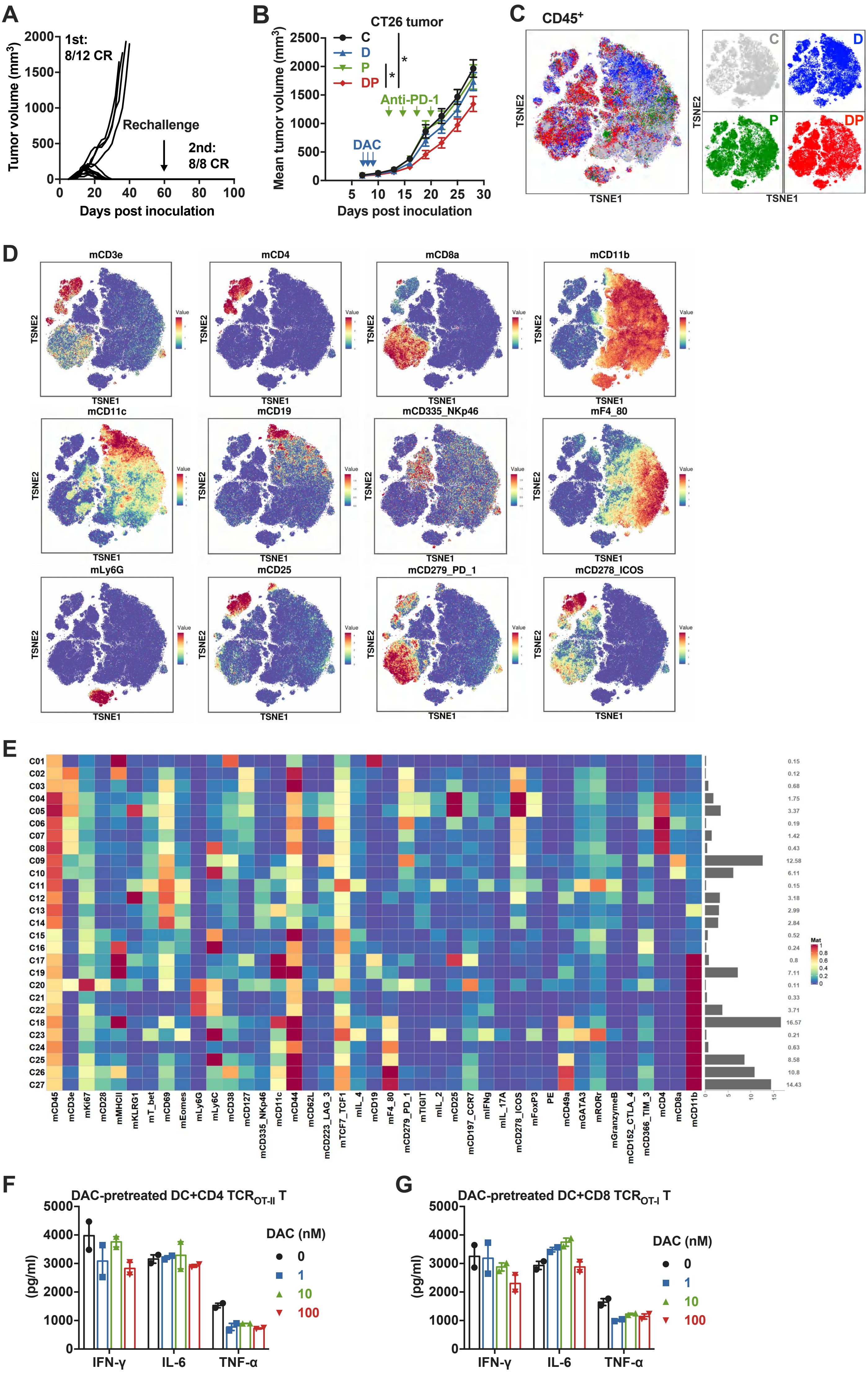

A
Tumor volume (mm3)
1st: 8/12 CR
Rechallenge
2nd: 8/8 CR
Days post inoculation
B
CT26 tumor
Mean tumor volume (mm3)
C
D
P
DP
Anti-PD-1
DAC
Days post inoculation
C
CD45+
TSNE1
TSNE2
D
mCD3e
mCD4
mCD8a
mCD11b
mCD11c
mCD19
mCD335_NKp46
mF4_80
mLy6G
mCD25
mCD279_PD_1
mCD278_ICOS
E
Mat
F
DAC-pretreated DC+CD4 TCROT-II T
(pg/ml)
DAC (nM)
IFN-γ
IL-6
TNF-α
G
DAC-pretreated DC+CD8 TCROT-I T

Figure S6. The representative FACS plots on MC38-bearing mice model.

(**A**) Representative FACS plots for $Ki67^+$ cells in $CD8^+$ TILs from MC38-OVA tumors. (**B**) Percentage of PD-1^+ cells in $CD8^+$ TILs from MC38-OVA tumors (n=7). One-way ANOVA analysis. (**C**) Representative FACS plots for PD-1^+TIM-3^+ and PD-1^+TIM-3^- $CD8^+$ TILs from MC38-OVA tumors and the frequencies are also shown. (**D**) Frequency of PD-1^{hi}TIM-3^+LAG-3^+ cells in $CD8^+$PD-1^+ cells from MC38-OVA tumors (n=4). One-way ANOVA analysis. (**E**) Representative FACS plots for TCF-1^+TIM-3^- and TCF-1^-TIM-3^+ $CD8^+$ TILs from MC38-OVA tumors and the frequencies are also shown. (**F**) Representative FACS plots for $Tetramer^+$ $CD8^+$ TILs from MC38-OVA tumors and the total numbers per 1×10^6 are also shown.

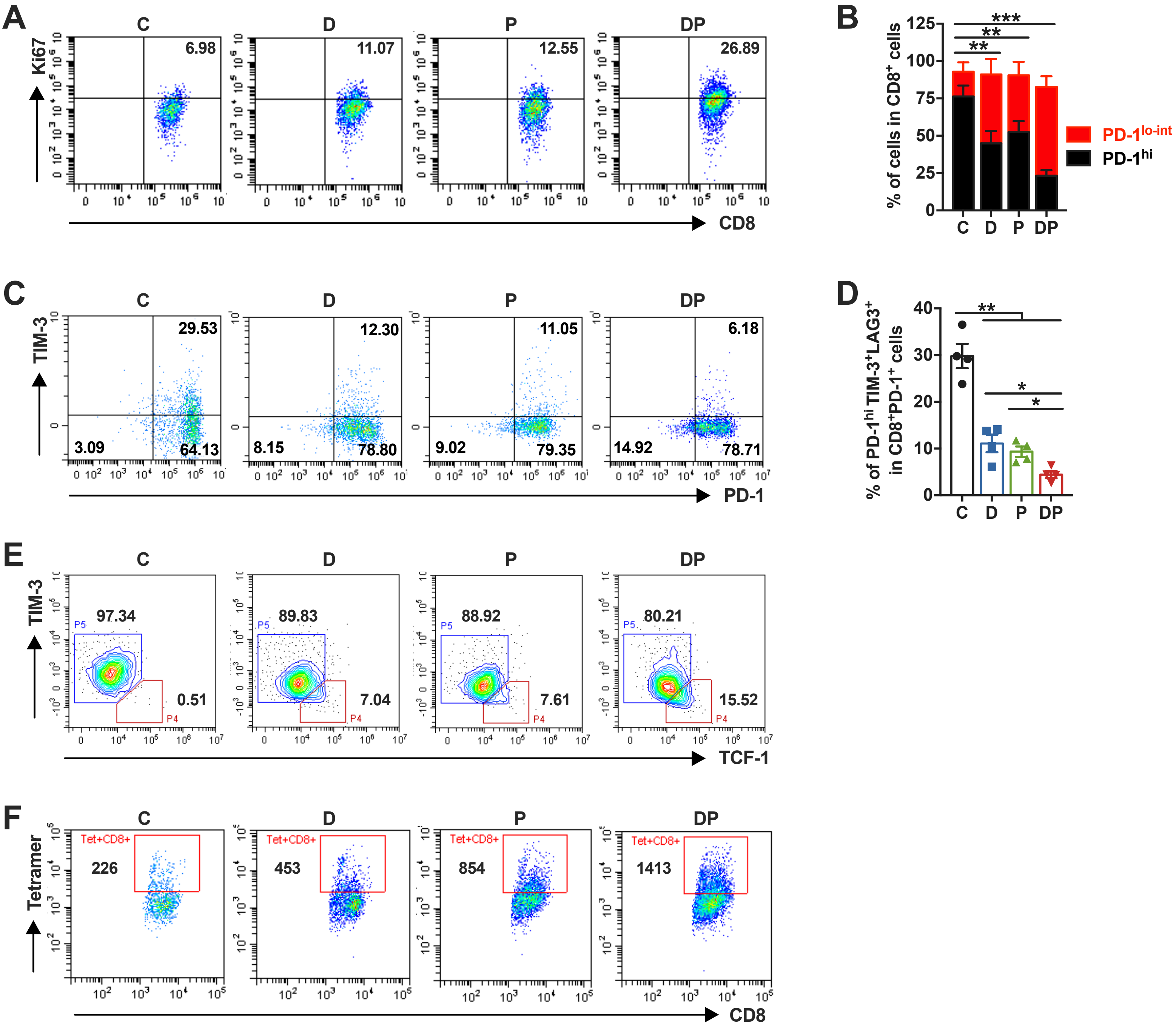

A
C
D
P
DP
Ki67
CD8
6.98
11.07
12.55
26.89
B
% of cells in CD8+ cells
PD-1lo-int
PD-1hi
***
**
**
C
TIM-3
PD-1
29.53
12.30
11.05
6.18
3.09
64.13
8.15
78.80
9.02
79.35
14.92
78.71
D
% of PD-1hi TIM-3+LAG3+ in CD8+PD-1+ cells
E
TIM-3
TCF-1
97.34
0.51
89.83
7.04
88.92
7.61
80.21
15.52
P5
P4
F
Tetramer
CD8
Tet+CD8+
226
453
854
1413

Figure S7. The effects of DP treatment on EG7-bearing and CT26-bearing mice models.

(**A-F**) C57BL/6J mice were implanted with 1×10^6 EG7-OVA cells, treated with PBS (C group; black), or decitabine alone (D group, blue), or anti-PD-1 antibody alone (P group, green) or decitabine plus anti-PD-1 (DP group, red) (n=3). (**A-B**) Absolute numbers of $CD8^+$ cells (**A**), $PD\text{-}1^+TIM\text{-}3^+$ and $PD\text{-}1^+TIM\text{-}3^-$ cells (**B**) per 10^6 total cells from EG7-OVA tumors. (**C**) Absolute number of tumor-infiltrated $tetramer^+CD8^+$ cells per 10^6 total cells from EG7-OVA tumors. The representative FACS plots for $tetramer^+$ $CD8^+$ T-cells and the total number per 10^6 cells are shown. (**D**) Absolute numbers of tumor-infiltrated $tetramer^+CD8^+PD\text{-}1^+TIM\text{-}3^+$ and $tetramer^+CD8^+PD\text{-}1^+TIM\text{-}3^-$ cells per 10^6 total cells from EG7-OVA tumors. (**E-F**) Absolute numbers of $PD\text{-}1^+IFN\text{-}\gamma^+TNF\text{-}\alpha^+$ in $CD8^+$ TILs (**E**) and $tetramer^+CD8^+$ TILs (**F**) per 10^6 total cells from EG7-OVA tumors. (**G-J**) Balb/c mice were implanted with 1×10^6 CT26 cells, treated with PBS (C group; black), or decitabine alone (D group, blue), or anti-PD-1 antibody alone (P group, green) or decitabine plus anti-PD-1 (DP group, red) (n=3). (**G**) Frequency of $CD8^+$ cells in $CD45^+$ cells from CT26 tumors. Frequency of $Ki67^+$ (**H**), $PD\text{-}1^+TIM\text{-}3^+$ and $PD\text{-}1^+TIM\text{-}3^-$ (**I**), and $IFN\text{-}\gamma^+$ (**J**) cells in $CD8^+PD\text{-}1^+$ TILs from CT26 tumors. Bar plots represent the mean ± SEM. * $p < 0.05$; ** $p < 0.01$; *** $p < 0.001$, by one-way ANOVA analysis.

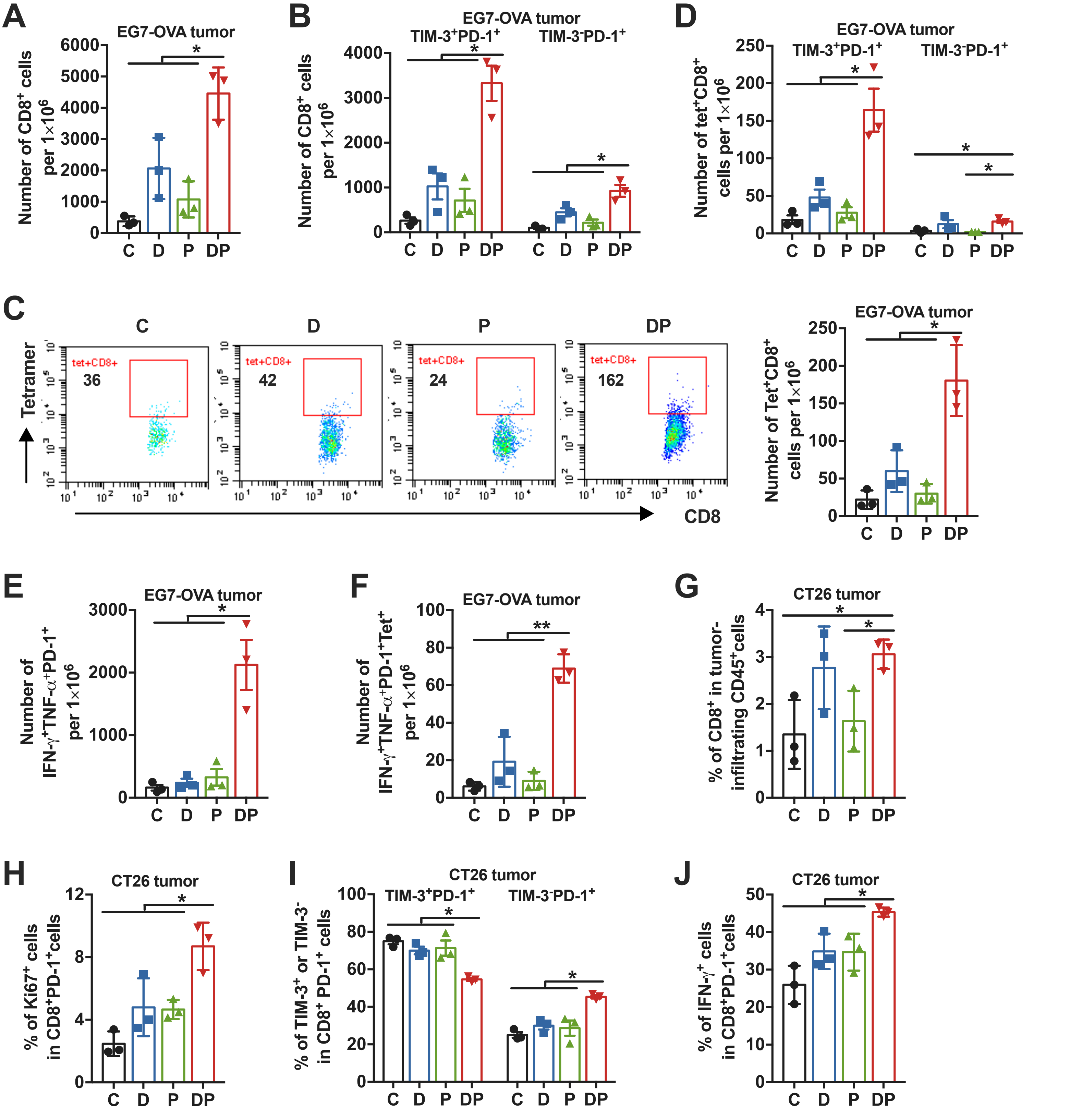
A
EG7-OVA tumor
Number of CD8+ cells per 1×10^6
C D P DP
B
EG7-OVA tumor
TIM-3+PD-1+
TIM-3-PD-1+
Number of CD8+ cells per 1×10^6
D
EG7-OVA tumor
Number of tet+CD8+ cells per 1×10^6
C
C D P DP
Tetramer
tet+CD8+
36
42
24
162
CD8
Number of Tet+CD8+ cells per 1×10^6
E
EG7-OVA tumor
Number of IFN-γ+TNF-α+PD-1+ per 1×10^6
F
EG7-OVA tumor
Number of IFN-γ+TNF-α+PD-1+Tet+ per 1×10^6
G
CT26 tumor
% of CD8+ in tumor-infiltrating CD45+cells
H
CT26 tumor
% of Ki67+ cells in CD8+PD-1+cells
I
CT26 tumor
TIM-3+PD-1+
TIM-3-PD-1+
% of TIM-3+ or TIM-3- in CD8+ PD-1+ cells
J
CT26 tumor
% of IFN-γ+ cells in CD8+PD-1+cells

Figure S8. The expression profile of CD3$^+$ T-cells.

(**A-B**) t-SNE plot of CD3$^+$ T-cells. Each dot indicates a single cell, colored by cluster (**A**), and sample (**B**). (**C**) Dot plot showing expression of selected marker genes per cluster. The size of the dot encoded the percentage of cells within a cell type in which that marker was detected, and its color encoded the average expression level. (**D**) t-SNE plot of CD3$^+$ T-cells colored by normalized expression of Cd3d, Cd8a, Cd8b1.

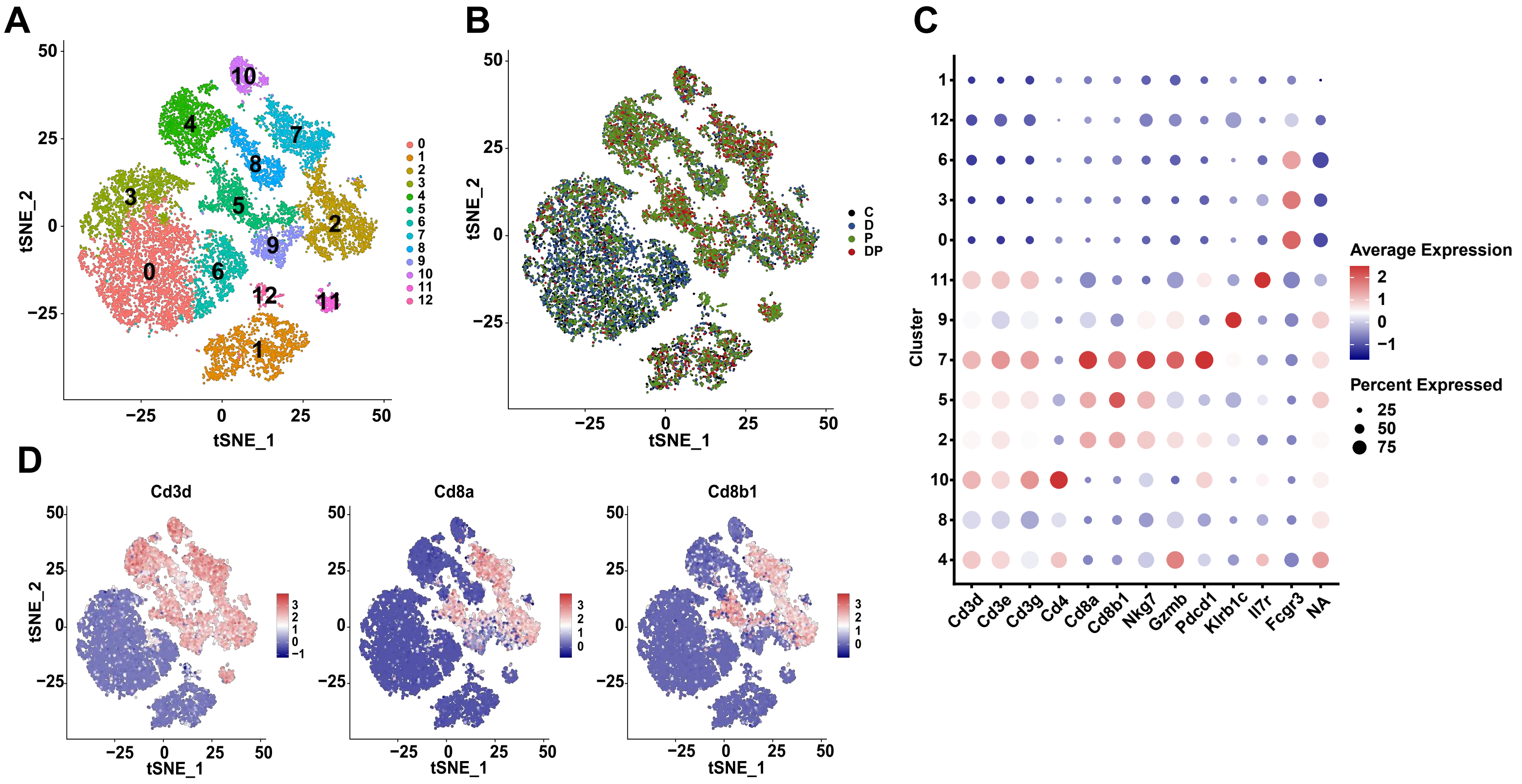
A
B
C
D
tSNE_1
tSNE_2
C
D
P
DP
Average Expression
Percent Expressed
Cluster
Cd3d
Cd3e
Cd3g
Cd4
Cd8a
Cd8b1
Nkg7
Gzmb
Pdcd1
Klrb1c
Il7r
Fcgr3
NA
Cd3d
Cd8a
Cd8b1

Figure S9. The quality control, cell distribution and expression level of $CD8^+$ T-cells.

(**A**) Quality control of scRNA-seq of $CD8^+$ T-cells. Violin plot showing the number of feature, count, and percent of mitochondrial counts in $CD8^+$ T-cells respectively. (**B-C**) t-SNE plot of $CD8^+$ T-cells. Each dot indicates a single cell, colored by sample (**B**), and phase (**C**). (**D**) t-SNE plot of $CD8^+$ T-cells, separated by sample. Each dot indicates a single cell, colored by cluster. (**E**) t-SNE plot of $CD8^+$ T-cells colored by normalized expression of marker genes.

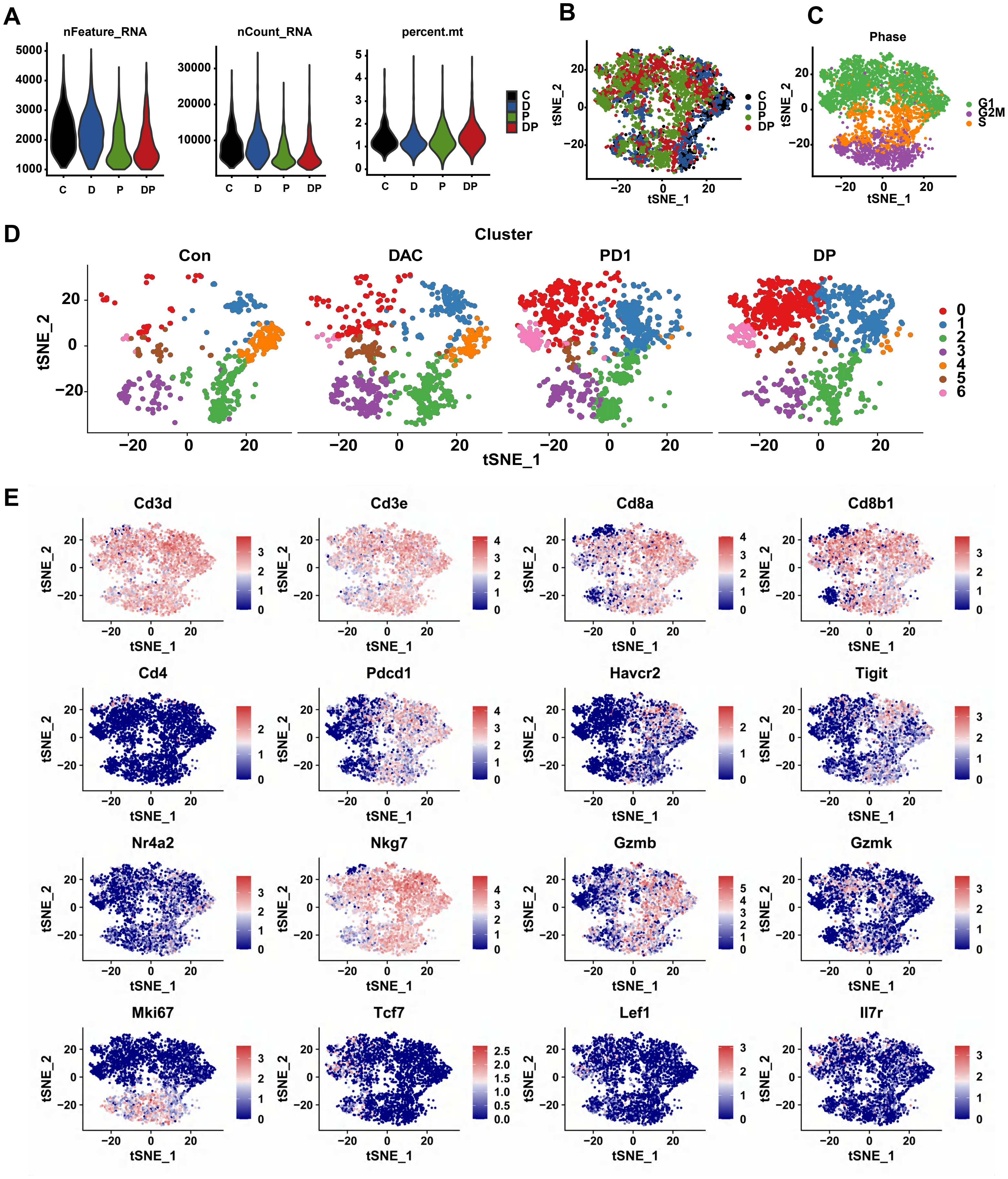
A
nFeature_RNA
nCount_RNA
percent.mt
C
D
P
DP
B
tSNE_1
tSNE_2
C
Phase
G1
G2M
S
D
Cluster
Con
DAC
PD1
DP
0
1
2
3
4
5
6
E
Cd3d
Cd3e
Cd8a
Cd8b1
Cd4
Pdcd1
Havcr2
Tigit
Nr4a2
Nkg7
Gzmb
Gzmk
Mki67
Tcf7
Lef1
Il7r

Figure S10. The number, trajectory and RNA velocity of $CD8^+$ T-cells.

(**A**) Bar plot showing the subtype cell number of $CD8^+$ T-cells per group. (**B**) Monocle trajectories of exhausted T-cells (C0, 1, 2 and 4) colored by cluster and separated by sample. (**C**) Diffusion Map visualizing differentiation of $CD8^+$ T-cells. Each dot for a single cell, colored by cluster. (**D-G**) Velocity analysis of $CD8^+$ T-cells in C group (**D**), DAC group (**E**), anti-PD-1 group (**F**) and decitabine-plus-anti-PD-1 group (G) revealing the origin and inter-relationship of $CD8^+$ T cell subpopulations. Velocity fields were projected onto the diffusion map.

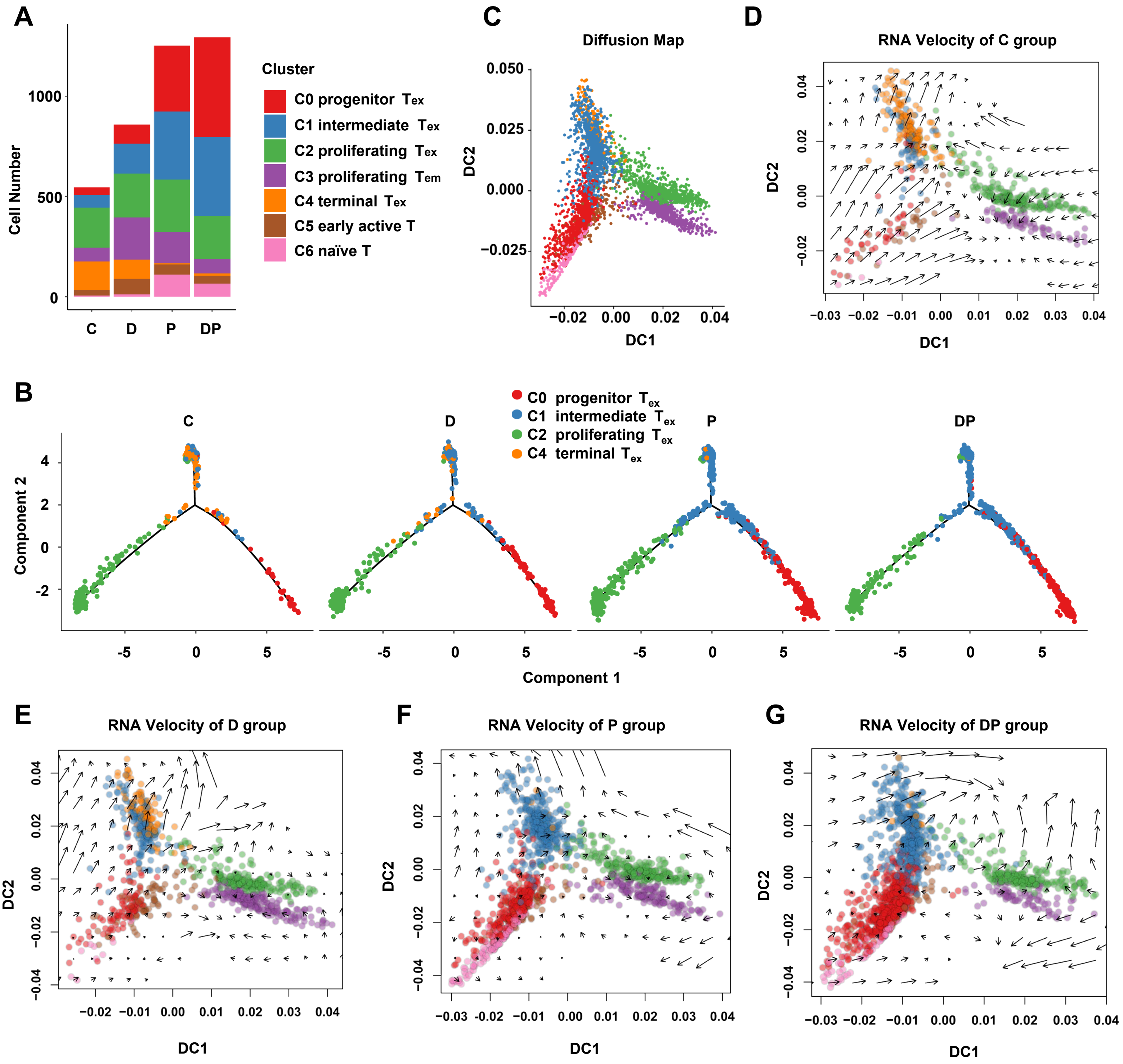
A
Cell Number
0
500
1000
C
D
P
DP
Cluster
C0 progenitor Tex
C1 intermediate Tex
C2 proliferating Tex
C3 proliferating Tem
C4 terminal Tex
C5 early active T
C6 naïve T
C
Diffusion Map
DC2
DC1
D
RNA Velocity of C group
B
C0 progenitor Tex
C1 intermediate Tex
C2 proliferating Tex
C4 terminal Tex
C
D
P
DP
Component 2
Component 1
E
RNA Velocity of D group
F
RNA Velocity of P group
G
RNA Velocity of DP group

Figure S11. The paired TCR profiles of $CD8^+$ T-cells.

(**A**) Bar plot showing the D50 diversity index, the number of clonotypes occupying the 50% repertoires. (**B**) The cell number of each cluster stratified by clone size. (**C**) The clonotype percentage of each group stratified by clone size. (**D**) The clonotype number of each group stratified by clone size. (**E**) The cell percentage of each group stratified by clone size. (**F**) The cell number of each group stratified by clone size. (**G**) The subtype cell percentage of expanding $CD8^+$ T-cells (cells with clone size ⩾ 2) per group. (**H**) Dot plot showing GO terms of upregulated genes of highly clonally expanded cells (cells with clone size ⩾ 10) between DP group versus P group. The size of the dot encoded the size of geneset in each GO term, and its color encoded the Benjamini-Hochberg adjusted p values. (**I**) Summary proportion of clonotypes with specific indices. (**J**) The cell number of top 50 clonotype of progenitor T_{ex} (C0), colored by group.

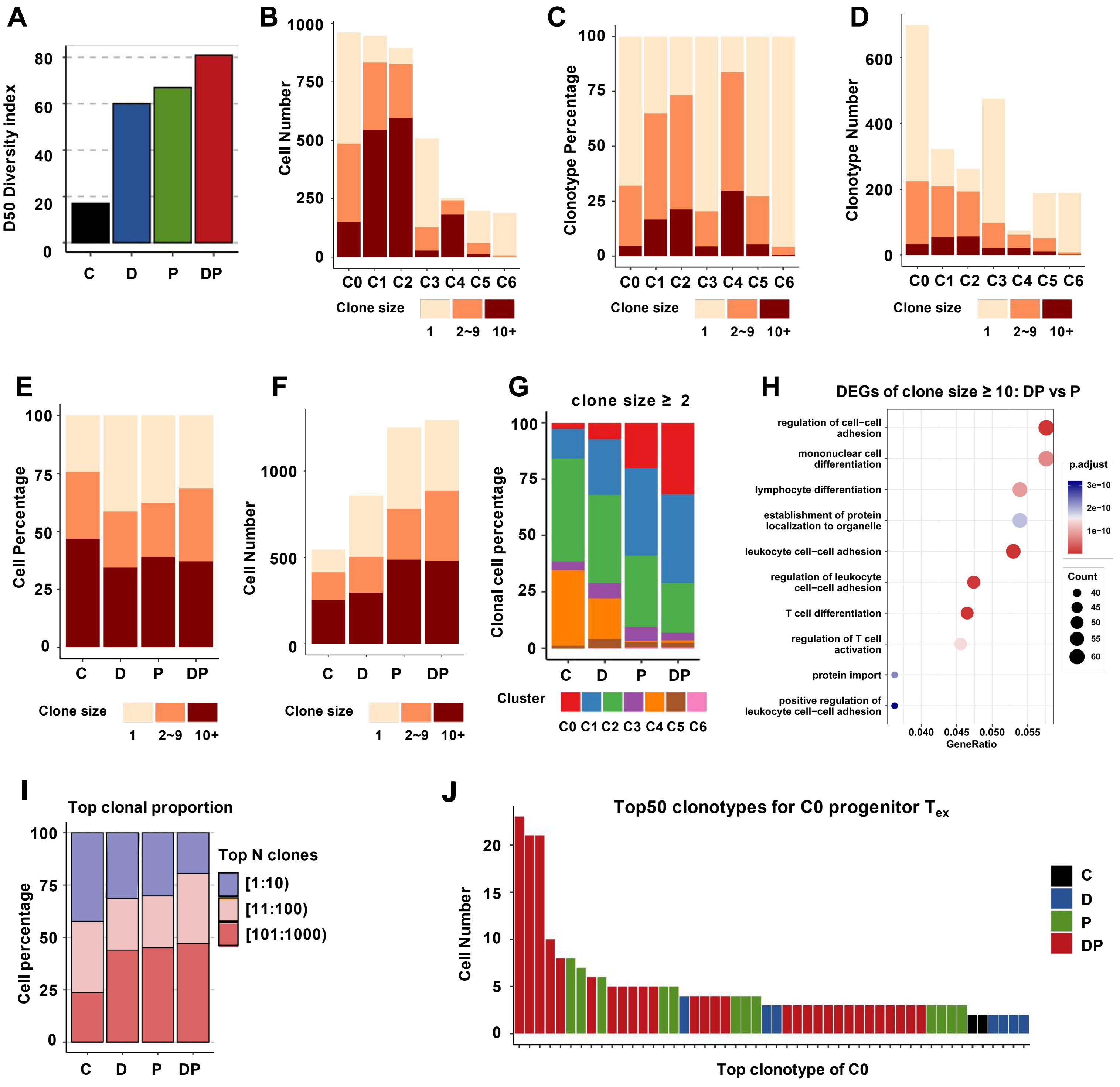
A
D50 Diversity index
0
20
40
60
80
C
D
P
DP
B
Cell Number
0
250
500
750
1000
C0 C1 C2 C3 C4 C5 C6
Clone size
1
2~9
10+
C
Clonotype Percentage
0
25
50
75
100
C0 C1 C2 C3 C4 C5 C6
Clone size
1
2~9
10+
D
Clonotype Number
0
200
400
600
C0 C1 C2 C3 C4 C5 C6
Clone size
1
2~9
10+
E
Cell Percentage
0
25
50
75
100
C
D
P
DP
Clone size
1
2~9
10+
F
Cell Number
0
500
1000
C
D
P
DP
Clone size
1
2~9
10+
G
clone size ≥ 2
Clonal cell percentage
0
25
50
75
100
C
D
P
DP
Cluster
C0 C1 C2 C3 C4 C5 C6
H
DEGs of clone size ≥ 10: DP vs P
regulation of cell−cell adhesion
mononuclear cell differentiation
lymphocyte differentiation
establishment of protein localization to organelle
leukocyte cell−cell adhesion
regulation of leukocyte cell−cell adhesion
T cell differentiation
regulation of T cell activation
protein import
positive regulation of leukocyte cell−cell adhesion
0.040
0.045
0.050
0.055
GeneRatio
p.adjust
3e−10
2e−10
1e−10
Count
40
45
50
55
60
I
Top clonal proportion
Cell percentage
0
25
50
75
100
C
D
P
DP
Top N clones
[1:10)
[11:100)
[101:1000)
J
Top50 clonotypes for C0 progenitor Tex
Cell Number
0
5
10
15
20
Top clonotype of C0
C
D
P
DP

Figure S12. Integrated analysis of scRNA-seq and ATAC-seq.

(**A**) Dot plot showing KEGG terms of upregulated genes of each module as in shown in Figure 7C. The size of the dot encodes the ratio of genes in each KEGG terms, and its color encodes the Benjamini-Hochberg adjusted p values. (**B**) Heatmap of enriched TF recorded in TARGET database across upregulated genes as shown in Fig.7C, calculated by metascape, colored by p-values. (**C**) Heatmap showing the Pearson correlation between ATAC-seq samples. (**D**) Venn plot showing the number of ATAC-seq peaks in each sample. (**E**) Boxplot showing the width of changed peak (DP vs P), two-sided unpaired t test. (**F**) Boxplot showing the ATAC-seq signal within all peaks (72100 peaks in three samples) in three groups, two-sided paired t test. (**G-H**) The enriched motifs in gained (**G**) and lost (**H**) peaks between DP versus P group. Motifs of TFs with Benjamini adjusted P-value less than 0.05 were colored and important TFs are labeled. FC (fold change) represents the ratio of the percentage of gained peaks with motif and the percentage of background peaks with motif. (**I**) Volcano plot showing the enriched motifs in gained and loss peaks between P versus C group. Motifs of TFs with Benjamini adjusted p-value less than 0.05 are colored and important TFs are labeled. FC (fold change) represents the ratio of the percentage of gained peaks with motif and the percentage of background peaks with motif. (**J**) Violin plot showing the expression level between P group and DP group in $CD8^+$ T-cells of some genes related to transcription factors in (**H**). Each dot represents a cell, two-sided unpaired wilcox test.

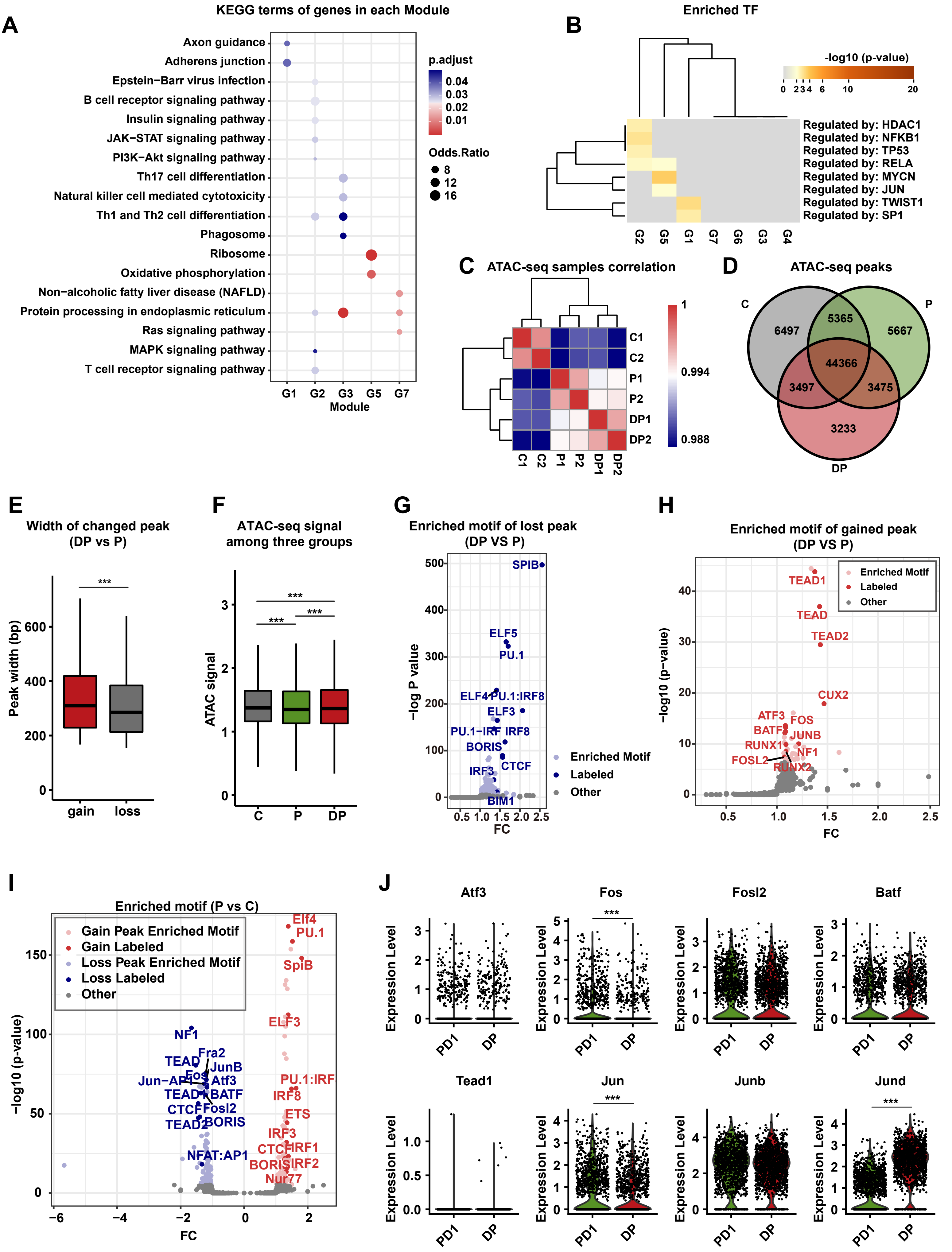

A
KEGG terms of genes in each Module
Axon guidance
Adherens junction
Epstein−Barr virus infection
B cell receptor signaling pathway
Insulin signaling pathway
JAK−STAT signaling pathway
PI3K−Akt signaling pathway
Th17 cell differentiation
Natural killer cell mediated cytotoxicity
Th1 and Th2 cell differentiation
Phagosome
Ribosome
Oxidative phosphorylation
Non−alcoholic fatty liver disease (NAFLD)
Protein processing in endoplasmic reticulum
Ras signaling pathway
MAPK signaling pathway
T cell receptor signaling pathway
G1 G2 G3 G5 G7
Module
p.adjust
0.04 0.03 0.02 0.01
Odds.Ratio
8 12 16
B
Enriched TF
-log10 (p-value)
0 2 3 4 6 10 20
Regulated by: HDAC1
Regulated by: NFKB1
Regulated by: TP53
Regulated by: RELA
Regulated by: MYCN
Regulated by: JUN
Regulated by: TWIST1
Regulated by: SP1
G2 G5 G1 G7 G6 G3 G4
C
ATAC-seq samples correlation
C1 C2 P1 P2 DP1 DP2
1 0.994 0.988
D
ATAC-seq peaks
C P DP
6497 5365 5667 44366 3497 3475 3233
E
Width of changed peak (DP vs P)
Peak width (bp)
gain loss
***
F
ATAC-seq signal among three groups
ATAC signal
C P DP
***
G
Enriched motif of lost peak (DP VS P)
−log P value
FC
SPIB ELF5 PU.1 ELF4 PU.1:IRF8 ELF3 PU.1−IRF IRF8 BORIS IRF3 CTCF BIM1
Enriched Motif
Labeled
Other
H
Enriched motif of gained peak (DP VS P)
−log10 (p−value)
FC
TEAD1 TEAD TEAD2 CUX2 ATF3 FOS BATF JUNB RUNX1 NF1 FOSL2 RUNX2
Enriched Motif
Labeled
Other
I
Enriched motif (P vs C)
Gain Peak Enriched Motif
Gain Labeled
Loss Peak Enriched Motif
Loss Labeled
Other
−log10 (p-value)
FC
Elf4 PU.1 SpiB ELF3 PU.1:IRF IRF8 ETS IRF3 CTCF IRF1 BORIS IRF2 Nur77
NF1 TEAD Fra2 JunB Fos Jun−AP1 Atf3 TEAD4 BATF CTCF Fosl2 TEAD2 BORIS NFAT:AP1
J
Atf3 Fos Fosl2 Batf
Tead1 Jun Junb Jund
Expression Level
PD1 DP
***

Figure S13. Integrated analysis of scRNA-seq, ATAC-seq, and JunD ChIP-seq.

(**A**) Venn plot showing the number of ATAC-seq peaks in our samples and JunD ChIP-seq. (**B**) Boxplot showing the ATAC-seq signal of ATAC-seq peaks with and without JunD binding in P and DP group, two-sided paired t test. (**C**) GO terms of DP gained peak (versus P group) related genes. (**D**) Heatmap showing the scaled activity of TFs calculated by SCENIC in T_{ex} cells of each group. (**E**) Integrated transcriptional regulatory network inferred by SCENIC showing the target genes of TF JunD. Dot size represents the importance of target genes. Color represents the average log2 FC between P versus C group.

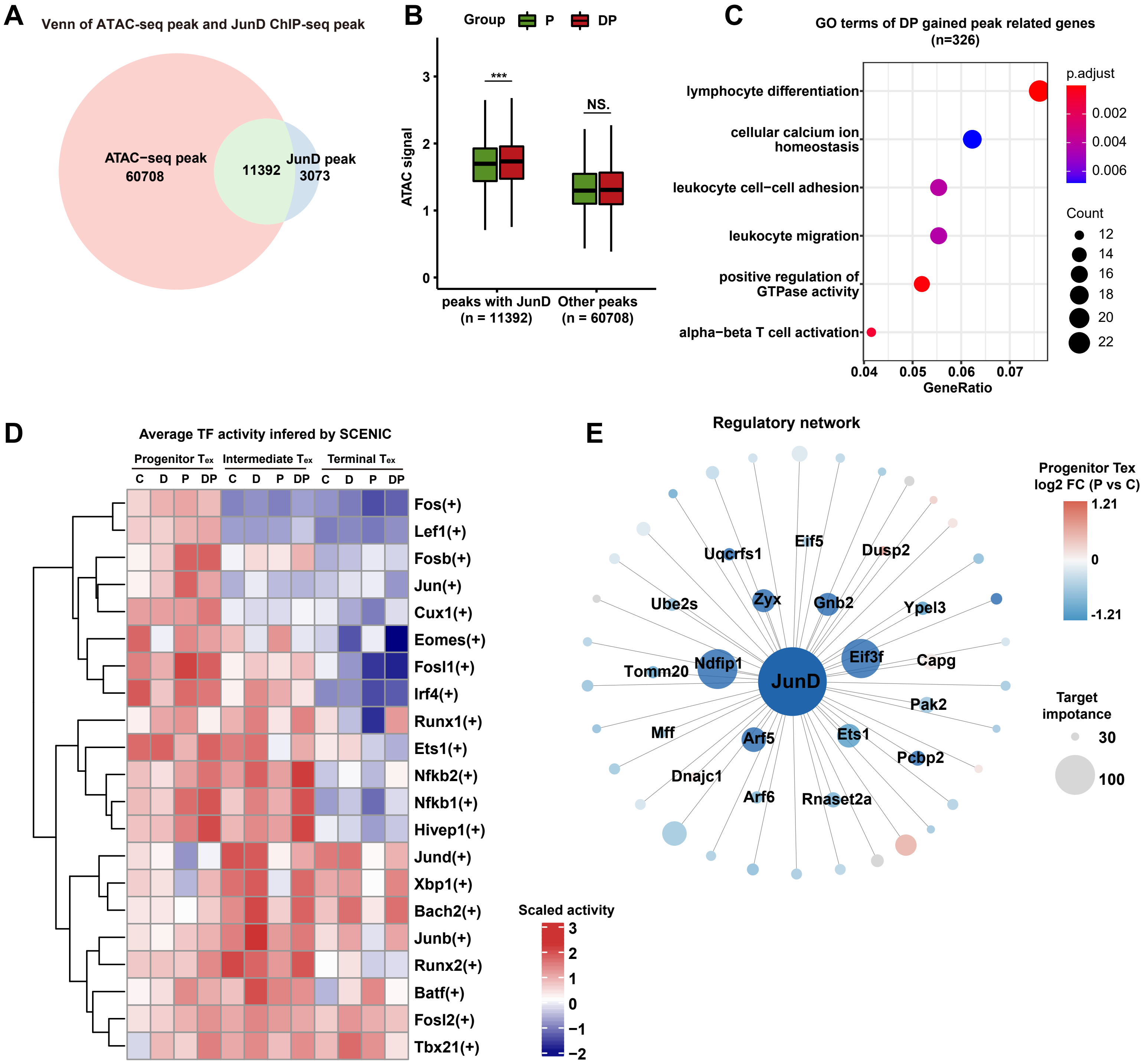

A
Venn of ATAC-seq peak and JunD ChIP-seq peak
ATAC−seq peak
60708
11392
JunD peak
3073
B
Group
P
DP
ATAC signal
***
NS.
peaks with JunD
(n = 11392)
Other peaks
(n = 60708)
C
GO terms of DP gained peak related genes
(n=326)
lymphocyte differentiation
cellular calcium ion homeostasis
leukocyte cell−cell adhesion
leukocyte migration
positive regulation of GTPase activity
alpha−beta T cell activation
GeneRatio
p.adjust
Count
D
Average TF activity infered by SCENIC
Progenitor Tex
Intermediate Tex
Terminal Tex
C D P DP C D P DP C D P DP
Fos(+)
Lef1(+)
Fosb(+)
Jun(+)
Cux1(+)
Eomes(+)
Fosl1(+)
Irf4(+)
Runx1(+)
Ets1(+)
Nfkb2(+)
Nfkb1(+)
Hivep1(+)
Jund(+)
Xbp1(+)
Bach2(+)
Junb(+)
Runx2(+)
Batf(+)
Fosl2(+)
Tbx21(+)
Scaled activity
E
Regulatory network
JunD
Uqcrfs1
Eif5
Dusp2
Ube2s
Zyx
Gnb2
Ypel3
Tomm20
Ndfip1
Eif3f
Capg
Pak2
Mff
Arf5
Ets1
Pcbp2
Dnajc1
Arf6
Rnaset2a
Progenitor Tex
log2 FC (P vs C)
1.21
0
-1.21
Target
impotance
30
100

Figure S14. The expression level of JunD in this study and public data.

(**A**) Violin plot showing the expression level of JunD in T_{ex} before and after imputation with scImpute. (**B**) Boxplot showing the expression level of JunD in $CD8^+$ TILs from patients with lung cancers before (pre) and after (post) combination therapy of PD-1 blockade with chemotherapy. The expression data is from Liu et al (http://nsclcpd1.cancer-pku.cn/); two-sided unpaired wilcox test. (**C**) The representative images of immunostaining results for CD8 (green) to highlight $CD8^+$ T-cells and JunD (red) in tumor samples from MC38-OVA-bearing mice in the indicated groups. DAPI (blue) is also shown as nuclear staining. (**D**) The percentage of tumor-infiltrated $CD8^+$ T-cells as in C. (**E**) The percentage of $JunD^+$ cells as in tumor-infiltrated $CD8^+$ T-cells as in C. (**F**) The Mean Fluorescence Intensity (MFI) of JunD in tumor-infiltrated $CD8^+$ T-cells as in C. (**G**) $CD8^+$ $TCR_{OT\text{-}I}$ T-cells were treated with PBS (C), decitabine (D), anti-PD-1 (P) or the combination (DP), and co-cultured with MC38-OVA cells for 3 days at E: T ratio of 1:2. Then $CD8^+$ T-cells were sorted and JunD mRNA levels were detected by qPCR. Bar plots represent the mean ± SEM. * $p < 0.05$; ** $p < 0.01$; *** $p < 0.001$, by one-way ANOVA analysis.

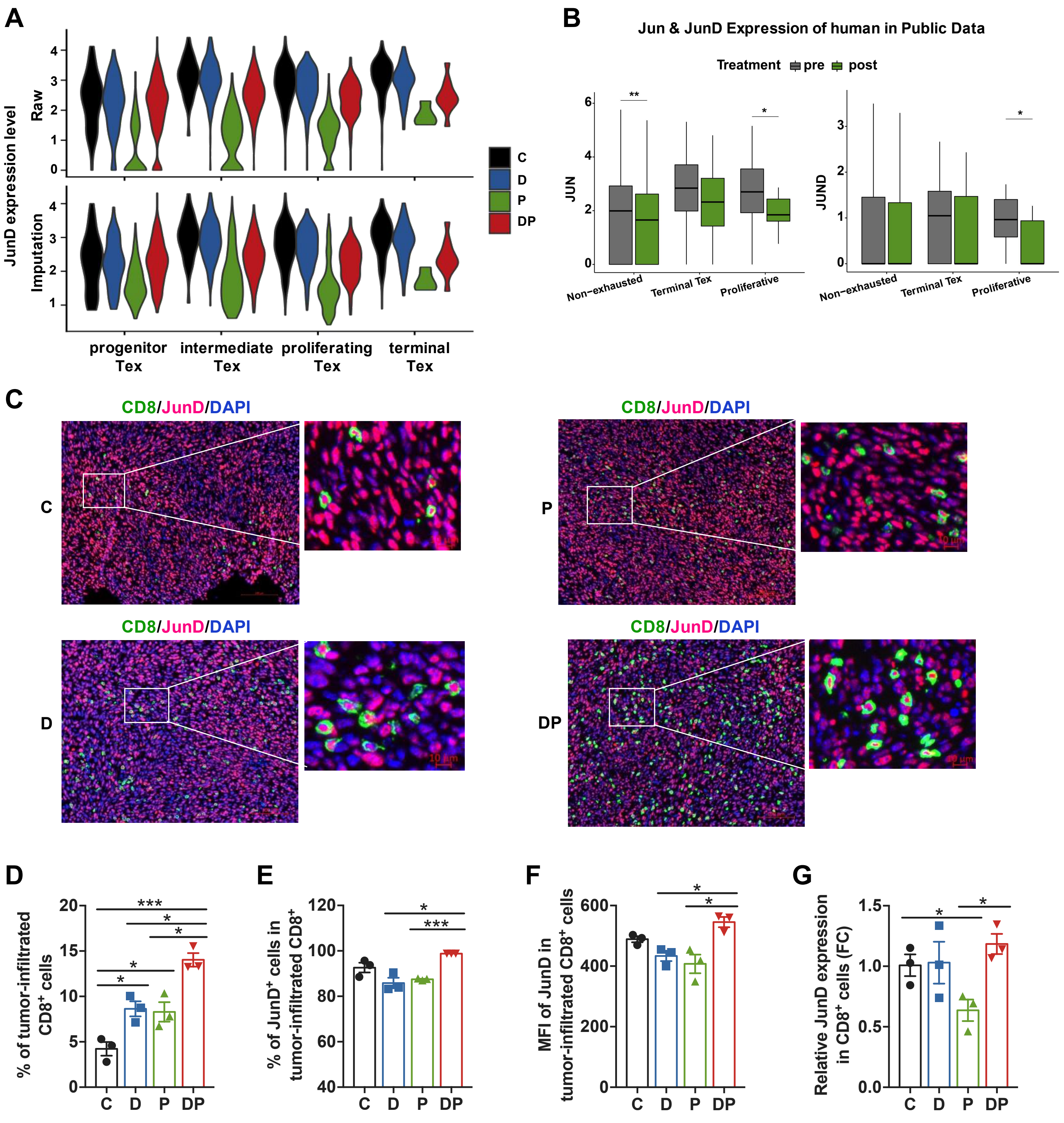

A
JunD expression level
Raw
Imputation
C
D
P
DP
progenitor Tex
intermediate Tex
proliferating Tex
terminal Tex
B
Jun & JunD Expression of human in Public Data
Treatment pre post
JUN
JUND
Non-exhausted
Terminal Tex
Proliferative
C
CD8/JunD/DAPI
C
P
D
DP
D
% of tumor-infiltrated CD8+ cells
E
% of JunD+ cells in tumor-infiltrated CD8+
F
MFI of JunD in tumor-infiltrated CD8+ cells
G
Relative JunD expression in CD8+ cells (FC)

Figure S15. The transcriptional and epigenetic changes in $CD8^+$ T_{ex} following DP treatment.

(**A**) Bar plot showing the normalized enrichment score (NES) from ranked list of genes expressed in proliferating T-cells from in DP group versus P group, calculated from gene set enrichment analysis (GSEA). Gene signatures are from immunologic signature gene sets of MSigDB. (**B**) GSEA of the indicated signature (GSE41867) from the ranked list of genes in terminal T_{ex} (C4). FDR, false discovery rate. (**C**) Frequency of IFN-γ^+TNF-α^+ cells in Slamf6$^-$TIM-3$^+$PD-1$^+$ terminal T_{ex} which was differentiated from Slamf6$^+$TIM-3$^-$PD-1$^+$ progenitor T_{ex} after co-culture with MC38-OVA cells at the indicated times. One-way ANOVA analysis. (**D**) GSEA of the indicated signature (GSE20754) from the ranked list of genes from ACT model in DP group versus P group. (**E-G**) ATAC-seq tracks of $CD3^+$ T-cells per group at the *Bcl-2*, *Camk2d* and *Stat4* loci (n=2 per group). The ChIP-seq peaks of $CD8^+$ T-cells are from Pauken et al. (**H**) Boxplot showing T cell activation score of proliferating T-cells from ACT model in cells with different JunD levels. The cells are divided into 4 groups according to the expression level of JunD with quartile intervals. JunD level group 1, 2, 3, and 4 represent a quarter of cells with JunD expression levels from low to high, respectively. One-way ANOVA analysis. * $p < 0.05$; ** $p < 0.01$; *** $p < 0.001$.

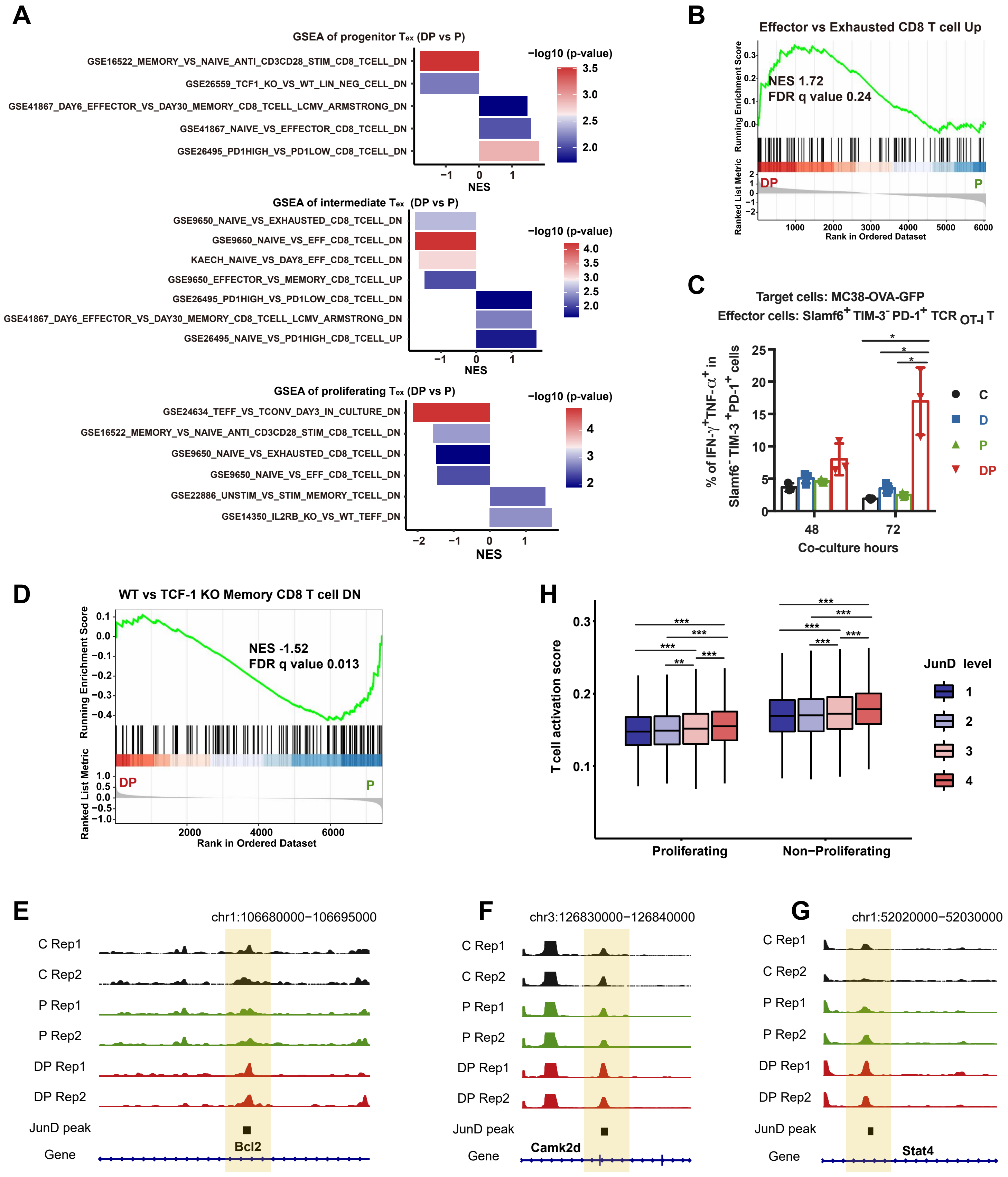
A
GSEA of progenitor Tex (DP vs P)
GSE16522_MEMORY_VS_NAIVE_ANTI_CD3CD28_STIM_CD8_TCELL_DN
GSE26559_TCF1_KO_VS_WT_LIN_NEG_CELL_DN
GSE41867_DAY6_EFFECTOR_VS_DAY30_MEMORY_CD8_TCELL_LCMV_ARMSTRONG_DN
GSE41867_NAIVE_VS_EFFECTOR_CD8_TCELL_DN
GSE26495_PD1HIGH_VS_PD1LOW_CD8_TCELL_DN
−log10 (p-value)
NES
GSEA of intermediate Tex (DP vs P)
GSE9650_NAIVE_VS_EXHAUSTED_CD8_TCELL_DN
GSE9650_NAIVE_VS_EFF_CD8_TCELL_DN
KAECH_NAIVE_VS_DAY8_EFF_CD8_TCELL_DN
GSE9650_EFFECTOR_VS_MEMORY_CD8_TCELL_UP
GSE26495_PD1HIGH_VS_PD1LOW_CD8_TCELL_DN
GSE41867_DAY6_EFFECTOR_VS_DAY30_MEMORY_CD8_TCELL_LCMV_ARMSTRONG_DN
GSE26495_NAIVE_VS_PD1HIGH_CD8_TCELL_UP
GSEA of proliferating Tex (DP vs P)
GSE24634_TEFF_VS_TCONV_DAY3_IN_CULTURE_DN
GSE16522_MEMORY_VS_NAIVE_ANTI_CD3CD28_STIM_CD8_TCELL_DN
GSE9650_NAIVE_VS_EXHAUSTED_CD8_TCELL_DN
GSE9650_NAIVE_VS_EFF_CD8_TCELL_DN
GSE22886_UNSTIM_VS_STIM_MEMORY_TCELL_DN
GSE14350_IL2RB_KO_VS_WT_TEFF_DN
B
Effector vs Exhausted CD8 T cell Up
NES 1.72
FDR q value 0.24
Running Enrichment Score
Ranked List Metric
DP
P
Rank in Ordered Dataset
C
Target cells: MC38-OVA-GFP
Effector cells: Slamf6+ TIM-3- PD-1+ TCR OT-I T
% of IFN-γ+TNF-α+ in Slamf6- TIM-3+PD-1+ cells
C
D
P
DP
48
72
Co-culture hours
D
WT vs TCF-1 KO Memory CD8 T cell DN
NES -1.52
FDR q value 0.013
Running Enrichment Score
Ranked List Metric
DP
P
Rank in Ordered Dataset
H
T cell activation score
JunD level
Proliferating
Non−Proliferating
E
chr1:106680000−106695000
C Rep1
C Rep2
P Rep1
P Rep2
DP Rep1
DP Rep2
JunD peak
Gene
Bcl2
F
chr3:126830000−126840000
Camk2d
G
chr1:52020000−52030000
Stat4

Figure S16. Suppressed antitumor capacity of DP combination in JunD knockout $CD8^+$ T-cells.

(**A**) Purified naïve $CD8^+$ T-cells from $TCR_{OT\text{-}I}$ mice were activated, treated with PBS (C), anti-PD-1 antibody (P) or decitabine-plus-anti-PD-1 (DP) as shown. Before co-cultured with MC38-OVA cells, the indicated T-cells were incubated with JNK activator Anisomycin (0.5 μM, 1 μM) for 24 hours. These $CD8^+$ T-cells were then co-cultured with MC38-OVA cells at E: T ratio of 1:2. Frequency of IFN-γ^+TNF-α^+ T-cells at 2 days of co-culture by flow cytometry analysis. One-way ANOVA analysis. (**B**) *JunD* editing efficiency in $TCR_{OT\text{-}I}$ T-cells with different sgRNAs as evaluated by TIDE. (**C**) Western Blot analysis of JunD expression in $CD8^+$ $TCR_{OT\text{-}I}$ T-cells. JunD sgRNA #2 was used in the following function experiments (D-G). (**D**) Frequency and representative FACS plots for $Ki67^+$ cells in NC and JunD KO $TCR_{OT\text{-}I}$ T-cells. Student's t-test. (**E-G**) NC and JunD KO $TCR_{OT\text{-}I}$ T-cells were pretreated with PBS (Con), 10 nM decitabine (DAC), anti-PD-1 or the combination, and co-cultured with MC38-OVA-GFP cells at E: T ratio of 1:2. (**E**) Frequency of live GFP^+ MC38-OVA target cells after co-culture with the indicated T-cells for 4 hours. One-way ANOVA analysis. (**F**) Absolute number of GFP^+ MC38-OVA target cells after co-culture with the indicated T-cells for 48 hours. One-way ANOVA analysis. (**G**) Frequency of IFN-γ^+TNF-α^+ cells in $CD8^+$ T-cells after co-culture with the indicated T-cells for 48 hours. Two-way ANOVA analysis. * $p < 0.05$; ** $p < 0.01$; *** $p < 0.001$.

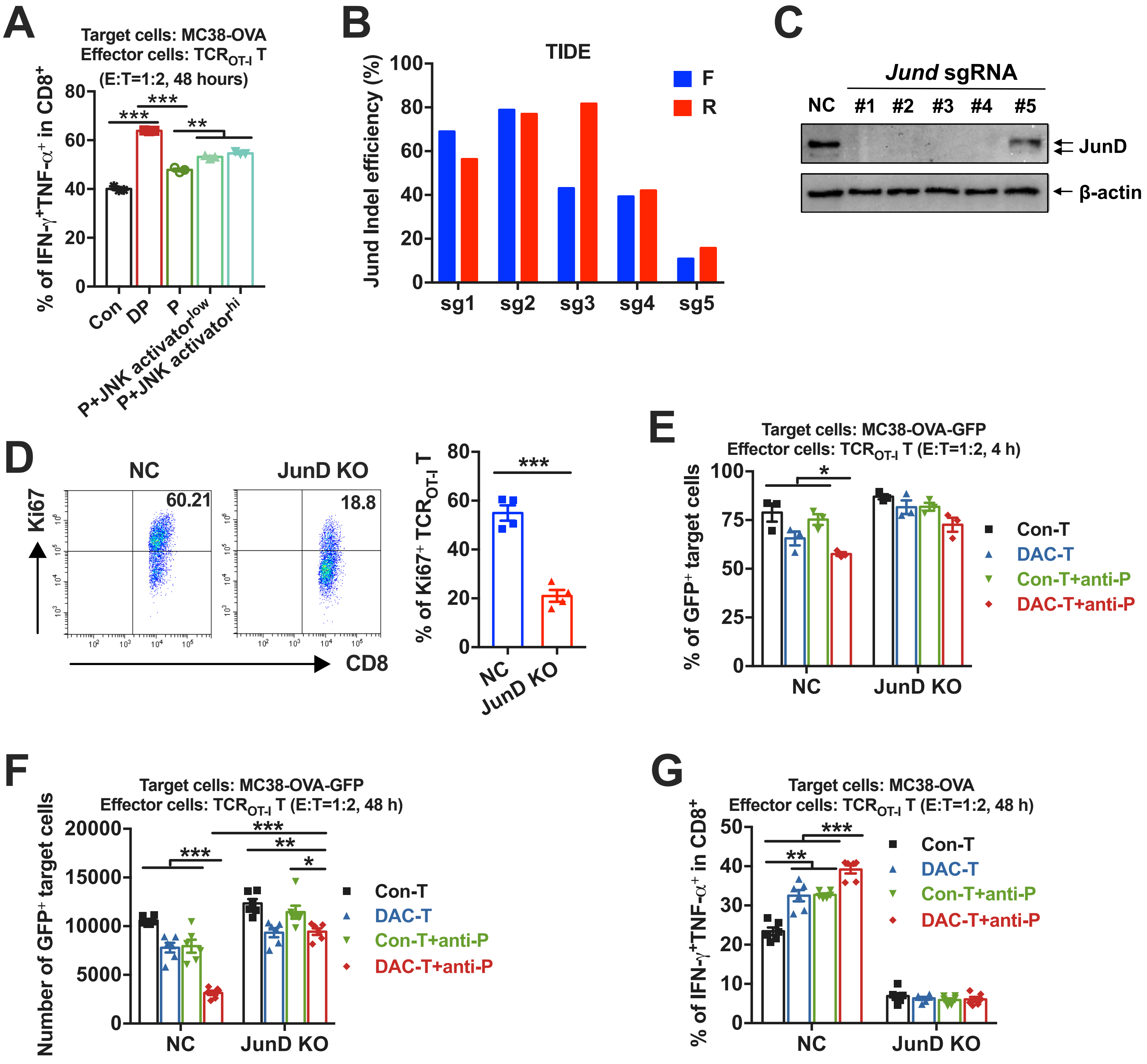
A
Target cells: MC38-OVA
Effector cells: TCR$_{OT-I}$ T
(E:T=1:2, 48 hours)
% of IFN-γ$^+$TNF-α$^+$ in CD8$^+$
Con
DP
P
P+JNK activator^{low}
P+JNK activator^{hi}
B
TIDE
Jund Indel efficiency (%)
F
R
sg1
sg2
sg3
sg4
sg5
C
Jund sgRNA
NC
#1
#2
#3
#4
#5
JunD
β-actin
D
NC
JunD KO
Ki67
CD8
60.21
18.8
% of Ki67$^+$ TCR$_{OT-I}$ T
E
Target cells: MC38-OVA-GFP
Effector cells: TCR$_{OT-I}$ T (E:T=1:2, 4 h)
% of GFP$^+$ target cells
Con-T
DAC-T
Con-T+anti-P
DAC-T+anti-P
F
Target cells: MC38-OVA-GFP
Effector cells: TCR$_{OT-I}$ T (E:T=1:2, 48 h)
Number of GFP$^+$ target cells
G
Target cells: MC38-OVA
Effector cells: TCR$_{OT-I}$ T (E:T=1:2, 48 h)
% of IFN-γ$^+$TNF-α$^+$ in CD8$^+$

Figure S17. Clinical trial of DP combination therapy in patients with advanced gastrointestinal tumors.

(**A**) Best change from baseline in tumor burden per patient. (**B**) The PET-CT scans of the representative patient during treatment. Patient 1 had a metastatic gastric cancer (top) and disease progressed after two cycles of anti-PD-1 monotherapy (middle), and acquired a partial response after two cycles of decitabine-plus-anti-PD-1 antibody camrelizumab (bottom) in our clinical trial.

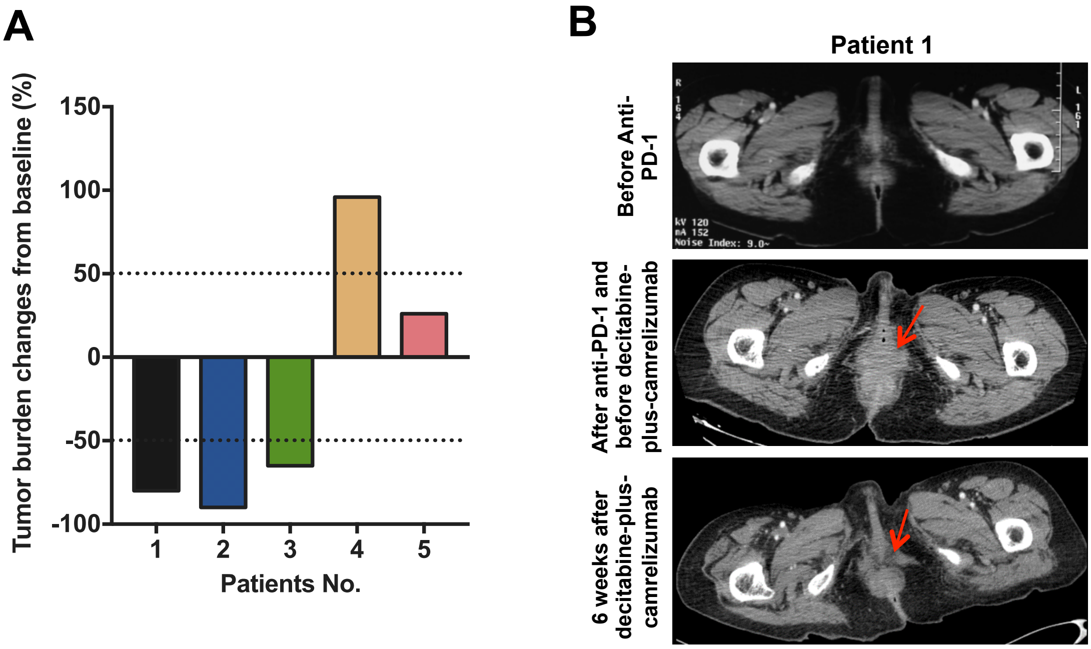
A
Tumor burden changes from baseline (%)
150
100
50
0
-50
-100
1
2
3
4
5
Patients No.
B
Patient 1
Before Anti-PD-1
After anti-PD-1 and before decitabine-plus-camrelizumab
6 weeks after decitabine-plus-camrelizumab

Figure S18. The effect of DP combination in $CD4^+$ T-cells.

(**A-E**) C57BL/6J mice were transplanted with 1.5×10^5 MC38-OVA cells, treated with PBS (C), or decitabine alone (D), or anti-PD-1 antibody alone (P) or decitabine-plus-anti-PD-1 combination (DP) as previously reported. (**A**) Frequency of $CD4^+$ TILs among $CD45^+$ cells. (**B**) Absolute number of $CD4^+$ TILs per 1×10^6 total cells. (**C**) Frequency of $Ki67^+$ cells in $CD4^+$ TILs. (**D**) Frequency of IFN-γ^+ Th1 subset, IL-4^+ Th2 subset, IL-17^+ Th17 subset and $Foxp3^+CD25^+$ Treg cells in total $CD4^+$ TILs. (**E**) Frequency of IFN-γ^+TNF-α^+ cells in $CD4^+$PD-1^+ cells. Bar plots represent the mean ± SEM. * $p < 0.05$; ** $p < 0.01$; *** $p < 0.001$, by one-way ANOVA analysis.

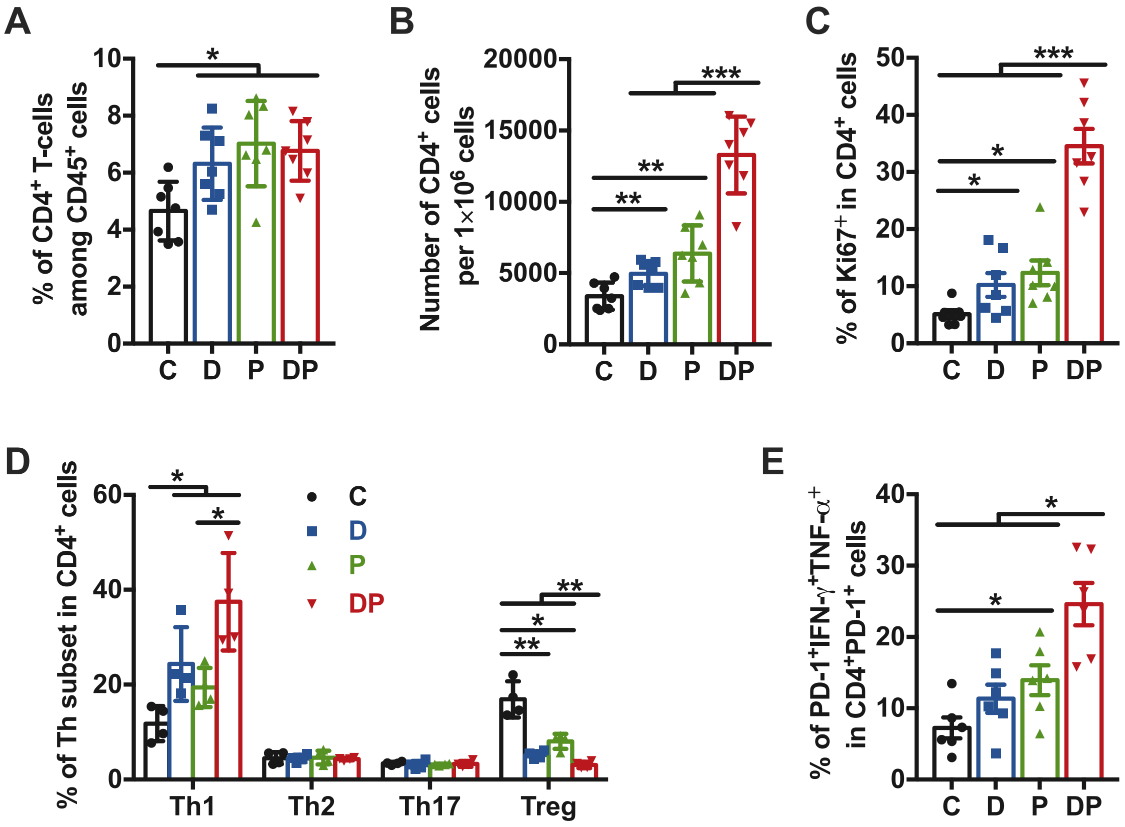
A
% of CD4+ T-cells among CD45+ cells
C D P DP
*
B
Number of CD4+ cells per 1×10^6 cells
C D P DP
** ** ***
C
% of Ki67+ in CD4+ cells
C D P DP
* * ***
D
% of Th subset in CD4+ cells
Th1 Th2 Th17 Treg
C D P DP
* * ** * **
E
% of PD-1+IFN-γ+TNF-α+ in CD4+PD-1+ cells
C D P DP
* *

Supplemental Tables

Table S1. Metal-conjugated antibodies used in CyTOF analysis.

Metal	Antibody names	Clone	Company
115ln	CD3	145-2C11	Biolegend
141Pr	CD28	37.51	Biolegend
142Nd	MHCII	M5/114.15.2	BioXcell
143Nd	KLRG1	2F1	Invitrogen
144Nd	CD185 (CXCR5)	L138D7	Biolegend
145Nd	CD152 (CTLA4)	UC10-4B9	Biolegend
146Nd	Eomes	DAN11MAG	Invitrogen
147Sm	CD86	GL-1	Biolegend
148Nd	Ly6C	HK1.4	Biolegend
149Sm	CD38	90	Biolegend
150Nd	CD49b	DX5	Biolegend
151Eu	CD44-h/m	IM7	Biolegend
152Sm	CD11c	N418	Biolegend
153Eu	CD62L	MEL-14	Biolegend
154Sm	Ki67	SolA15	Invitrogen
155Gd	CD223 (LAG-3)	C9B7W	Biolegend
156Gd	TCF-1/TCF-7	812145	RD
157Gd	TIGIT (VSTM3)	2190A	RD
158Gd	CD19	6D5	Biolegend
159Tb	F4/80	C1: A3-1	Bio-Rad
160Gd	CD206	C068C2	Biolegend
161Dy	CD279 (PD-1)	29F.1A12	Biolegend
162Dy	CD183 (CXCR3)	CXCR3-173	Biolegend
163Dy	CD25	3C7	Biolegend
164Dy	CD197 (CCR7)	4B12	Biolegend
165Ho	CD278 (ICOS)	C398.4A	Biolegend
166Er	Ly6G	IA8	Biolegend
168Er	FoxP3	FJK-16s	Invitrogen
169Tm	PE	PE001	Biolegend
170Er	T-bet	4B10	Biolegend
171Yb	CD69	H1.2F3	Biolegend
172Yb	FITC	FIT-22	Biolegend
173Yb	Granzyme B	GB11	Invitrogen
174Yb	IFN-γ	XMG1.2	Biolegend
175Lu	CD196 (CCR6)	29-2L17	Biolegend
176Yb	CD366 (TIM-3)	RMT 3-23	Biolegend
197Au	CD4	RM4-5	Biolegend
198pt	CD8a	53-6.7	Biolegend
209Bi	CD11b	M1/70	PLT

Table S11. Patients baseline characteristics and clinical responses to decitabine-plus-camrelizumab combination therapy.

Patient No.	Age (years)	Sex	ECOG	Diagnosis /Stage	Prior Treatment			Decitabine-plus-anti-PD-1 camrelizumab-plus-PT* combination	
					Chemotherapy	Anti-PD-1 antibody (cycles)	Best clinical response to anti-PD-1	Treatment cycles	Best clinical response
1	57	F	2	GC /metastatic	XELOX×8; Decitabine+FOLFOX×4; Decitabine+FOLFIRI	Yes (2)	PD	2	PR
2	54	M	1	EC /metastatic	Decitabine+PT×2	Yes (3)	PD	4	PR
3	47	M	2	GC /metastatic	XELOX×8; DOS×4; Decitabine+FOLFIRI;	Yes (4)	PD	3	PR
4	58	M	2	CRC /metastatic	XELOX×9; FOLFIRI×2	Yes (2)	PD	2	PD
5	48	F	2	BC /metastatic	CTX+EPI×4; PTX×4; Decitabine+PT×8; GEX+DDP×4	Yes (2)	PD	2	SD

* PT regimen (paclitaxel-albumin 180-220 mg/m^2 on day 6 and cisplatin 60-70 mg/m^2 on days 6-7).

M, male; F, female; GC, gastric cancer; EC, esophageal cancer; CRC, colorectal cancer; BC, breast cancer; PR, partial response; SD, stable disease; PD, progressive disease.

Table S2. The differentially methylated region (DMR), related genes and KEGG terms between D versus C group.

Table S3. The DEGs, related GO and GSEA results of scRNA-seq from ACT assays.

Table S4. TCR information of $CD8^+$ T-cells.

Table S5. The DEGs and related GO results of highly clonally expanded $CD8^+$ T-cells.

Table S6. The DEGs, related GO and KEGG results of progenitor T_{ex}.

Table S7. ATAC-seq peaks information, genes assigned to changed peaks and enriched TF of changed peaks.

Table S8. JunD ChIP-seq peaks information, related genes and GO results.

Table S9. Averaged TF activity and transcriptional regulatory network inferred by SCENIC.

Table S10. The GSEA results of DP group versus P group.

Table S12. The quality control (QC) information of scRNA-seq and scTCR-seq.

These tables are uploaded in Supplementary table.zip.
